# Conservation of intron and intein insertion sites: implications for life histories of parasitic genetic elements

**DOI:** 10.1186/1471-2148-9-303

**Published:** 2009-12-31

**Authors:** Kristen S Swithers, Alireza G Senejani, Gregory P Fournier, J Peter Gogarten

**Affiliations:** 1 Department of Molecular and Cell Biology University of Connecticut, 91 North Eagleville Road Storrs CT 06269-3125 USA; 2 Current address: Departments of Therapeutic Radiology and Genetics, Yale University School of Medicine, PO Box 208040 New Haven, CT 06520-8040 USA

## Abstract

**Background:**

Inteins and introns are genetic elements that are removed from proteins and RNA after translation or transcription, respectively. Previous studies have suggested that these genetic elements are found in conserved parts of the host protein. To our knowledge this type of analysis has not been done for group II introns residing within a gene. Here we provide quantitative statistical support from an analyses of proteins that host inteins, group I introns, group II introns and spliceosomal introns across all three domains of life.

**Results:**

To determine whether or not inteins, group I, group II, and spliceosomal introns are found preferentially in conserved regions of their respective host protein, conservation profiles were generated and intein and intron positions were mapped to the profiles. Fisher's combined probability test was used to determine the significance of the distribution of insertion sites across the conservation profile for each protein. For a subset of studied proteins, the conservation profile and insertion positions were mapped to protein structures to determine if the insertion sites correlate to regions of functional activity. All inteins and most group I introns were found to be preferentially located within conserved regions; in contrast, a bacterial intein-like protein, group II and spliceosomal introns did not show a preference for conserved sites.

**Conclusions:**

These findings demonstrate that inteins and group I introns are found preferentially in conserved regions of their respective host proteins. Homing endonucleases are often located within inteins and group I introns and these may facilitate mobility to conserved regions. Insertion at these conserved positions decreases the chance of elimination, and slows deletion of the elements, since removal of the elements has to be precise as not to disrupt the function of the protein. Furthermore, functional constrains on the targeted site make it more difficult for hosts to evolve immunity to the homing endonuclease. Therefore, these elements will better survive and propagate as molecular parasites in conserved sites. In contrast, spliceosomal introns and group II introns do not show significant preference for conserved sites and appear to have adopted a different strategy to evade loss.

## Background

Inteins are intervening polypeptide sequences that are translated as part of a protein [1-4], and are removed in the maturation of the final protein product. Some inteins contain a homing endonuclease [5] that has a large specific recognition site (12-40 base pairs). The intein-encoding DNA is inserted in frame within a host gene; after translation the intein catalyzes its own excision resulting in removal of the intein (internal protein) and splicing of the extein (external protein, the mature active host protein) (see [6] for detailed review) [2,7,8]. Comparative analyses have shown that all inteins are homologs; however, their sequences are so divergent that phylogenetic analyses of inteins inserted into different host proteins remains largely unresolved [4,9]. Inteins that are found in different insertion sites of the same host protein are not necessarily closely related to each other, and often highly divergent. However, inteins inserted into the same site in orthologous proteins are closely related to each other and share a common ancestor, but their molecular phylogeny does not always reflect the history of the host protein or of the host organism [4,10,11], indicating transfer of the intein between divergent hosts.

Introns are defined as non-coding regions of a gene that are excised during post-transcriptional processing. Since their discovery in 1977 [12] three major groups of introns have been identified: group I, group II, and spliceosomal introns. Group I and group II introns have distinct structures that facilitate their self-splicing activity (see [13,14] for detailed review), and they often encode an open reading frame (ORF) or contain an internal ORF [15-17]. The internal ORF of the group I introns encodes a homing endonuclease and the ORF of the group II introns encodes proteins with one to four of the following functionally defined domains: reverse transcriptase (RT), maturase, DNA-binding protein, and endonuclease [17,18]. These proteins serve two functions for the intron: assisting in splicing and folding, and allowing the intron to act as a mobile element and invade intron-free alleles via retrohoming or retrotransposition [13,17-19].

Endonucleases [4] provide mobility to some inteins and introns, through a process called "homing" [15,16,20]. These endonucleases are known as homing endonucleases (HE). The HEs initiate homing by cleaving the HE free allele. Similar to traditional restriction endonucleases, the HE makes a double strand break, and the HE containing element is copied during repair into the intein/intron free allele [15]. The HE recognition site is accessible in the host gene when flanking regions of the intron or intein integration sites are joined. Presence of the HE containing element makes the allele resistant to HE digestion. Free-standing HE genes also function as molecular parasites/symbionts [21-24], and can provide mobility for neighbouring HE-less group I introns through a collaborative homing mechanism [23].

Previous work found inteins and group I introns in conserved parts of their host genes [4,25-27], while group II introns were shown not to target conserved genes [28,29]. Here we provide quantitative support and extend previous analyses to other proteins that host inteins, group I, group II and spliceosomal introns across all three domains of life.

## Results

### Where are inteins and spliceosomal introns located in their host sequence and structure?

Our earlier analyses of three host proteins, ATPase catalytic subunit, replication factor C (RFC), and cell division control protein 21 (CDC21), confirmed the notion that inteins appear at highly conserved sites within their host proteins [4]. Since publication of [4] the number of inteins discovered in these three proteins has increased substantially, including some found in new insertion sites.

**Figure 1:**
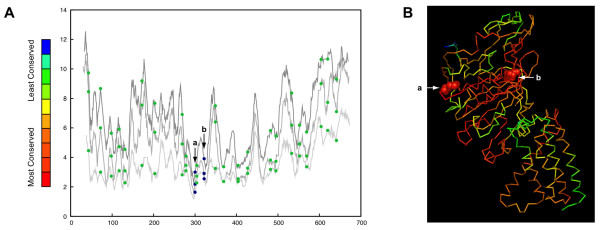
**Positions of inteins and introns along the protein sequence (panel A) and in the structure of vacuolar/archaeal ATPase catalytic subunit (panel B)**. Panel A shows the conservation profiles of subunit A of the V/A ATPase and the beta subunit of the F-type ATPase. The abscissa shows the amino acid position along the alignment; the ordinate designates the number of different amino acids present in that position averaged over a window of size 11. The dark grey line is the conservation profile of all three domains. The medium gray line is the conservation profile of Eukaryotes and Archaea. The light gray line is the conservation profile of the Archaea. The positions of inteins are indicated as blue dots with arrows. Positions of spliceosomal introns from are indicated as green dots without arrows. **Panel B **shows the structure of ATPase catalytic subunit A structure from *Pyrococcus horikoshii *OT3 (PDB ID: 1VDZ[68]) colored according to sequence conservation. The arrows indicate "a" and "b" intein insertion sites. "b" is the archaeal intein insertion site between Lys240 and Thr241 (both amino acids are shown with space-filled model) and "a" is the eukaryotic intein insertion site between Gly260 and Cys261 (space-filled model).

The vacuolar ATPase catalytic subunit hosts both inteins and spliceosomal introns in two intein insertion sites, "a" and "b". We find that these insertion sites are among the most conserved sites in the protein (*p *= 0.0099). The intein database InBase [30] lists 27 inteins in insertion site "a" and seven in insertion site "b" (Figure 1). Inteins in insertion site "a" are found in members of the Saccharomycetales and inteins in insertion site "b" are found in two orders of euryarchaeotes (Thermoplasmatales and Thermococcales) (Figure 1). The archaeal inteins are located 20 amino acids downstream of where the yeast inteins are located. The spliceosomal introns in this protein are not restricted to conserved sites (*p *= 0.3909). Inteins in positions "a" and "b" are mapped to the structure of the ATPase catalytic subunit of *Pyrococcus horikoshii *OT3 (Figure 1B). This mapping shows that both inteins are located in the conserved catalytic binding site of the subunit [31], suggesting that the presence of the intein prior to removal would disrupt catalytic activity of the subunit.

**Figure 2:**
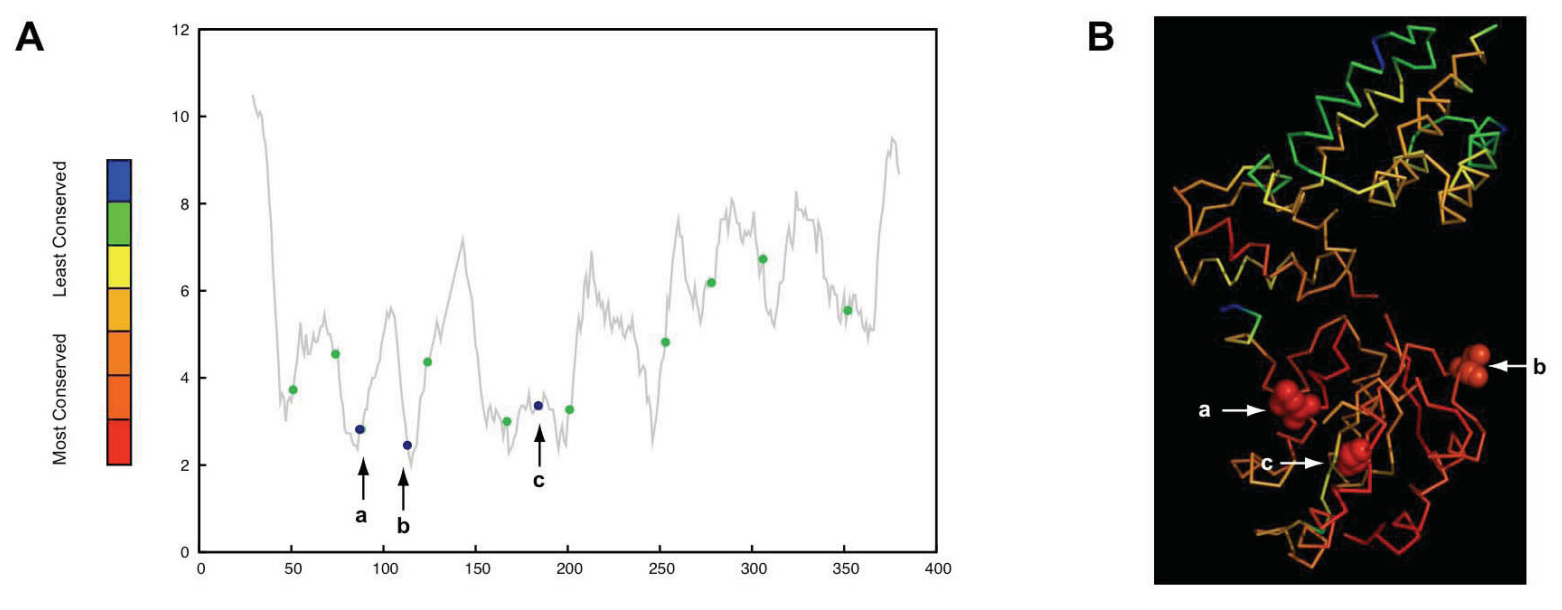
**Positions of inteins and spliceosomal introns along the protein sequence (panel A) and in the structure of the replication factor C (panel B)**. Panel A shows the conservation profile of the RFC protein (see figure 1 for details). The positions of inteins are indicated as blue dots with arrows and positions of spliceosomal introns are green dots without arrows. Panel B shows the structure of the *Archaeoglobus fulgidus *replication factor C (PDB ID: 2CHV[69]) colored according to sequence conservation. The arrows indicate intein insertion site "a" between Lys51 and THR52, "b" between Ala76 and Ser77, and "c" between Ser138 and Cys139 (all of these six amino acids are shown as space-filled model). All three intein insertion sites are conserved within the host protein.

The replication factor C (RFC) is less than 300 amino acids long, but accommodates inteins in three different sites (a-c) and spliceosomal introns in 10 different sites (Figure 2). InBase [30] reports 10 inteins located in these insertion sites; six in insertion site "a", two in insertion site "b" and two in insertion site "c" (Figure 2). The three insertions sites are among the most conserved parts of the host protein (*p *= 0.0209); and mapping of these sites on the structure of a RFC confirms that these sites are in conserved and centrally located regions of the protein. The 10 spliceosomal introns are not found to be in conserved sites of the protein (*p *= 0.2404).

**Figure 3:**
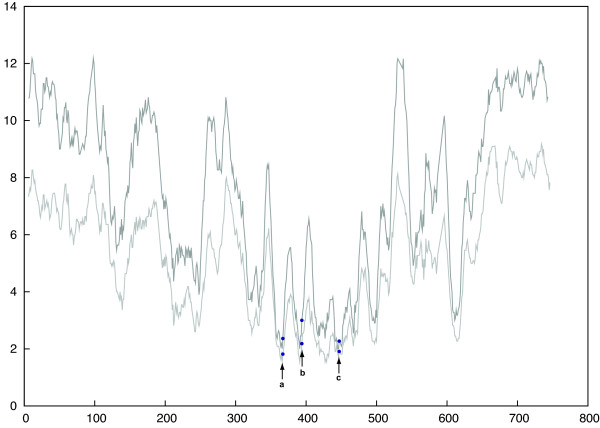
**Positions of inteins along the protein sequence of cell division control protein 21**. Dark gray indicates the conservation profile of all domains. Light gray indicates the conservation profile of Eukaryotes only. Inteins are found in three sites "a-c", shown with arrows pointing at blue dots.

The intein database currently list 16 inteins located in the CDC21 protein from nine various archaeal species. These inteins are found in three different sites: six are found in location "a", five in location "b", and three in location "c". Similar to the inteins found in the ATPase catalytic subunit and RFC, these inteins are found in highly conserved sites of the CDC21 protein (*p *= 0.0022) (Figure 3).

In addition, we analyzed all proteins that were reported in InBase [30] as containing an intein for conservation of the insertion site. With one exception all of these additional 30 proteins harbor their inteins in conserved regions of the respective host protein. The fillamentous hemmagglutinin protein was annotated by InBase as being an allele for an intein; however, this element has been shown to be a bacterial intein-like protein domain (BIL) [32]. BILs are found in non-conserved regions of hypervariable proteins and our analysis supports this notion as the BIL was not found to be in a conserved site. (See additional file 1 for profiles and *p*-values.) Using Fisher's combined probability method to calculate the overall significance level for inteins (omitting the BIL) inserting into conserved sites is *p *< 0.0001 (in calculating the combined probability, *p*-values for individual proteins *p *< 0.01 were considered as equal to 0.01).

### Where are group I and group II introns located in their host sequence and structure?

**Figure 4:**
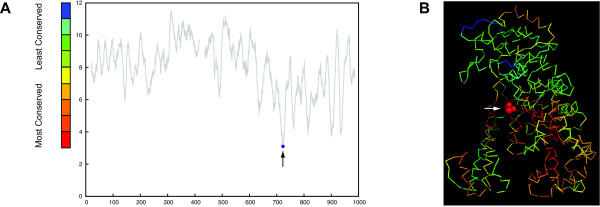
**Group I intron location along the protein sequence (panel A) and in the structure of the DNA polymerase I (panel B)**. Panel A shows conservation profile of DNA polymerase I (see figure one for details). Panel B shows the structure of the DNA polymerase I (Klenow Fragment) from Escherichia coli (PDB ID: 1KLN[70]) colored according to site conservation. The arrow indicates intron insertion site, of the intron found in homologous protein in Bacillus subtilis phage SPO1, which is between Asn675 and Leu676 (space-filled model).

We analyzed intron insertion positions for DNA polymerase I and cytochrome C oxidase subunit I. Analysis of group I introns containing HE ORFs indicated that, similar to inteins, these elements tend to target conserved sites of their host protein. The DNA polymerase I of Bacillus phage SPO1 contains a group I intron [33], which is found in a conserved site of the host protein (*p *= 0.022) (Figure 4).

The cytochrome C oxidase subunit I (*cox1*) gene found in the mitochondria of eukaryotes accommodates self-splicing group I and group II introns in more than forty different sites (See additional file 2 for intron insertion sites.). The number of introns found in different species varies. The *cox1 *gene of *Podospora anserine*, an ascomycete fungus, is more than 24 kb long and harbours 14 group I and two group II introns [34]. In *Saccharomyces cerevisiae *different strains host a total of six group I and three group II introns [18,35,36]. Similar to inteins, group I introns appear to target more conserved sites (*p *= 0.0003), and similar to spliceosomal introns, group II introns were found not to have a significant preference for conserved sites (*p *= 0.4176).

A linear regression analysis was performed to determine if there is a correlation between the number of fungal species that harbour the introns in a particular site and the conservation of the intron insertion site. A weak negative correlation between site conservation and number of species with group I introns is found at each site (*p *= 0.0609), while there is no correlation between site conservation and number of species with group II introns at each site (*p *= 0.2907). These findings suggest that for fungi, either group I introns tend to target conserved DNA sites more frequently, or group I introns survive in conserved sites for longer periods of time, while group II intron targeting is not strongly influenced by site conservation.

An additional eight proteins which host group I and group II introns were analyzed. These represent all protein families reported in the comparative RNA web site database [37] as containing group I and group II introns. These proteins followed a similar pattern where the group I introns showed a strong preference for conserved sites and the group II introns did not. Two exceptions were Chlorophyll alpha apoprotein A2 and NADH dehydrogenase subunit 3 where neither intron type showed a preference for conserved sites (See additional file 1 for *p*-values and profiles.).

**Figure 5:**
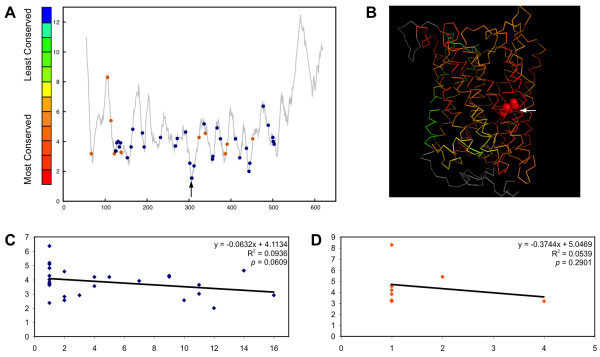
**Positions of introns along the protein sequence (panel A) and in the structure of the Cytochrome C Oxidase Subunit I (panel B)**. Panel A shows the conservation profile of the cytochrome C oxidase subunit I (see figure 1 for details). Blue dots indicate group I intron positions and orange dots represent group II intron positions. Panel B displays the structure of the *Paracoccus denitrificans *cytochrome C oxidase subunit I (PDB ID: 1QLE[71]) colored according to site conservation. The arrow points to the intron insertion site of the intron found in the homologous protein in some vascular plants, protist, fungi, green algae, liverworts, and amoeba. The insertion site is between Gly275 and His276 (space-filled model). Panel C shows the relationship between the number of group I introns found at each position and the site conservation. Panel D shows the relationship between the number of group II introns found at each position and the site conservation.

One of the group I introns in the *cox1 *gene is present in mitochondrial genomes of several vascular plants [38,39]. This intron, which encodes a homing endonuclease, seems to have been recently acquired via horizontal transfer from a fungal donor [38]. Our analyses showed that this intron is found in the most conserved site of the host protein (figure 5). The conservation of this site likely has played a crucial role in the successful transfer, allowing for HE target site recognition thereby facilitating the transfer of the intron between these distantly related organisms. In addition to vascular plants and fungi, many other eukaryotes host this intron, including the green algae *Marchantia, Chara*, and *Prototheca*, the liverworts *Pellia*, the soil-living amoeba *Dictyostelium*, and the single-celled protist *Monosiga *(see additional File 2).

The combined significance levels for group I and group II introns targeting conserved sites is *p *< 0.0001 and *p *= 0.49, respectively.

## Discussion

Introns have played a role in gene and genome evolution [40]; most or all of them may be later invaders of the genes in which they are currently located [41]. Interesting questions remain regarding the origin and evolution of introns, including: (1) How often did they arise? (2) How are they transmitted between divergent species? (3) How has their activity been maintained by natural selection? [16,17,20,42-44].

Regardless of their origin, many of the extant introns and inteins, especially the ones utilizing homing for survival, can be considered molecular parasites [20,45,46]. Some of these molecular parasites have acquired additional roles that are adaptive to the host [20,45,46]. While we discuss HE genes and self-splicing elements as molecular parasites that have their own life-cycles, this does not negate the fact that many of these elements adapted to play a beneficial or at least a necessary role for their host [47], and these roles may play a role in the long term survival of these elements [20].

In accordance with other studies [4,25,26], we find that inteins and group I introns are found in conserved regions of their respective host proteins. This same trend was also seen for 37 other protein families that host inteins and group I introns (see additional file 1). We also find that group I introns within highly conserved positions are more likely to be found in a broader range of species of fungi. Group II introns, BILs and spliceosomal introns do not reveal a significant bias toward conserved sites.

The splicing elements utilize a small portion of the flanking extein/exon region for splicing. Inteins are inserted before one of the following amino acids in the host protein: C, S, or T [6]. The amino acid is required to complete the self-splicing reaction. For the group I intron an internal guide sequence binds to 6 - 12 nucleotides of the exon. This internal guide sequence is not conserved between different group I introns and exact base pairing between the guide sequence and exon is not required [48]. Similarly, in the case of the group II introns two exon binding sites of the intron interact with six or more nucleotides each at the 5' and 3' flanking exon [13,49]. For both group I and group II introns this base pairing between intron internal sequence and the flanking exon restricts the possible sites in which an individual intron can retain its splicing activity. However, this does not restrict the locations of the introns as a group, because most mutations in the exon binding sites change the site specificity without impacting catalytic activity. Therefore, the splicing mechanism can be ruled out as a reason group I introns are found in conserved sites at the amino acid level. Exon splicing enhancer and silencer nucleotide motifs have been characterized surrounding individual spliceosomal introns [50,51]. In the case of spliceosomal introns these requirements for exon sequence motifs surrounding the intron have not led to a detectable preference of insertion sites that are conserved at the amino acid level.

Parasitic elements have likely evolved two different strategies to propagate and survive. Inteins and group I introns utilize homing endonucleases to target conserved sites. Conserved sites tend to be in functionally important; consequently, precise excision of the intein or group I intron is required to maintain functionality of the protein. Furthermore, the functional importance of the residues limits the range of substitutions that can modify the target sequence so that it no longer is recognized by the homing endonuclease. Although group I intron and intein insertions are targeted to a DNA sequence, this is merely a proxy for sequence conservation at the protein level, upon which purifying selection can act. Targeting conserved sites will also facilitate transfer of the intein or group I intron to new intron-less alleles, as they will likely contain near identical amino acid sequences in these regions. In contrast, group II introns use a more random and less specific retrohoming or retrotransposition mechanism (see [52] for review). This suggests an alternative strategy evolved by these elements, relying on frequent propagation to outpace more rapid loss. If group II introns are the ancestor of spliceosomal introns [28,53], it would not be unexpected to find a similar site preference for group II and spliceosomal introns.

Both of these strategies have successfully ensured the survival and propagation of inteins, and group I and group II introns [15,18,20]. These genetic elements can be considered molecular parasites that have their own life cycle, only occasionally evolving functions that contribute to the fitness of the host organism, or that increase the complexity of the host in an irreversible manner, without necessarily increasing the host's fitness [47,54].

In most eukaryotes the *coxI *gene (encoding cytochrome C oxidase subunit I) is found in the mitochondrion, and hosts many introns representing both group I and group II introns. Several studies provide evidence for horizontal gene transfer of this element between distantly related groups of eukaryotes, confirming an intron homing model of evolution [38,55,56].

As shown in figure 5, the *cox1 *gene introns were found in both conserved and variable regions of the host; however, introns that are found in numerous species are more often found in highly conserved sites. The only group I intron found in the *cox1 *gene of several vascular plants [39] is inserted in the most conserved site of the host protein. The intron present in plants was reportedly acquired from a fungal donor [38]. As the conservation of the insertion site is the key for homing process, the observed preference for conserved sites may reflect the mode of propagation of the group I introns.

It is possible that inteins and group I introns target all sites but only the ones that end up in a conserved site are retained. The two exceptional proteins in this study, NADH dehydrogenase subunit 3 and Chlorophyll alpha apoprotein A2, may be an example of this. This mechanism would result in a preference for conserved sites even in the absence of a site specific homing mechanism. Such independent preference for conserved sites may have caused splicing elements (introns and inteins) and homing endonucleases to target the same sites, resulting in the fusion of these elements [57]. Free-standing HE genes are found in intergenic regions, their survival is dependent on the homing cycle [23,24] and they might be more easily eliminated as compared to HE associated with self-splicing elements since their removal does not need to be precise. Upon fixation of the HE in the population, homing is no longer possible, because the HE is already present in all target sites. If the HE has not acquired another function that can create a selection pressure to maintain the HE gene, it is likely to decay and be lost [20].

The fact that BILs do not show a conserved site preference [58] suggests that the conserved site preference is associated with the life-cycle of the homing endonuclease containing molecular parasite. This life cycle was first formulated as a homing cycle involving movement of the molecular parasite across population or species boundaries [4,59], but it has been suggested that this life cycle, with its succession of empty target sites, sites invaded by a molecular parasite with functioning homing endonuclease, sites containing a dysfunctional homing endonuclease, can operate within in spatially distributed population [20], and for some values of fitness reduction of individuals carrying molecular parasites may also operate continuously in homogenous well mixed populations [60]. As discussed above, both the invasion phase and the deletion phase of life cycle may cause a conserved site preference. The targeting of a conserved site will make it more difficult for the host protein to evolve immunity towards the homing endonuclease, and it will make deletion of the molecular parasite more difficult.

The rate and tendencies to gain and lose introns vary considerably between lineages of eukaryotes [42,43], with the number of spliceosomal introns per gene and the degree of sequence conservation at spliceosomal intron boundaries also varying greatly [61]. Spliceosomal introns can be gained via homologous recombination with intron-containing genes unrelated to the conservation of the site, and through retrohoming [42,62,63]. The intron removal processes also appears to be unrelated to the sequence conservation at the insertion sites. The best characterized model for intron loss in multicellular eukaryotes is via homologous recombination between intron containing genes and spliced cDNAs produced by reverse transcription [42,62,63].

## Conclusions

We have provided statistical support for the notion that inteins and group I introns target conserved protein sites for survival. This may also provide evidence for the homing cycle that describes the life cycle of these two molecular parasites. Furthermore, our findings suggest that group II and spliceosomal introns persist in their host genes using a different evolutionary strategy.

## Methods

### Construction of the Conservation Profiles

To calculate conservation profiles along a protein sequence, BLASTP [64] was used to detect homologous protein sequences from the NCBI protein database. Each dataset was aligned using the CLUSTALW program version 1.83 [65], and inspected for alignment accuracy.

To construct archaeal/vacuolar-type ATPase catalytic subunit A protein sequence alignments sequences from 29 Eukaryotic species, 13 Archaeal species, and four Bacterial species were aligned. Ten Archaeal species and fifteen Eukaryotic species were aligned to construct the Replication factor C protein sequence alignments. Nine Archaeal species and 21 Eukaryotic species were aligned to construct the cell division control protein 21 protein sequence alignment. Sequences from two Eukaryotic species, 18 Bacterial species and four phage sequences were used to construct the DNA polymerase I protein sequence alignments. 21 Eukaryotic species and nine Archaeal species were use for the Cytochrome C Oxidase Subunit I protein alignment. All other proteins found in the InBase database [30] (data retrieved November 2009) that host inteins were also analyzed. And all other proteins found in the comparative RNA web site database [37] (data retrieved November 2009) were analyzed for group I introns and group II introns positions. (See additional file 3 for accession numbers.)

The conservation profiles were calculated from the protein alignments using an in house PERL script (Olga Zhaxybayeva, Dalhousie University, see additional file 4). This program calculates the number of substitutions over a sliding window of 11 aligned positions and the window is moved through the alignment one position at a time. Inteins, introns, and sites where more than 50% of the sequences had a gap inserted into the alignment are omitted in the calculations. The lower the conservation score the more conserved the position is and the higher the conservation score the less conserved the positions is. These conservation scores for each protein were mapped on the protein structures using MacPyMOL [66].

### Statistical Analysis

For each protein, n individual intron/intein insertions were given a probability score (*p*) based on the probability of a random position within the conservation profile containing an equal or greater conservation score than that at the position of the intron/intein insertion. If the protein contained more than one of each parasitic element these probabilities were then combined for each protein using Fisher's combined probability test [67], resulting in an overall probability (p*) of intron/intein insertions being randomly distributed within each conservation profile. To show that no one kingdom overwhelmingly contributed to the significance of p* each kingdom was removed from the alignment and a new conservation profile was made and the Fisher's combined probability test was performed (see Table 1). To test the applicability of Fisher's combined probability test we calculated the combined probability for the insertion of the two inteins in VMA intein dataset by calculating the sum of the conservation scores for all possible window pairs. The probability of the sum of two randomly chosen windows having a sum conservation score greater than the two intein sites was *p *= 0.0063, compared to *p *= 0.0099 as determined by the Fisher's combined test statistic. This shows that the Fisher's combined test provides a conservative measure of significance for these analyses.

**Table 1:** Statistical Support for Inteins and Introns Targeting Conserved Sequences.

		Intein	Group I Intron	Group II Intron	Spliceosomal Intron
**VMC**	All	0.0099			0.3909
	Eukaryote	0.0541			0.3303
	Archaea	0.0213			0.599
	Bacteria	0.0117			0.1899

**RFC**	All	0.0209			0.2404
	Eukaryote	0.0028			0.2024
	Archaea	0.0551			0.246

**CDC21**	All	0.0022			
	Eukaryote	0.0036			
	Archaea	0.002			

**POL**	All	0.0011			
	Bacteria	0.0070			
	Phage	0.0011			

**COX**	All		0.0003	0.4176	
	Eukaryote		<0.0001	0.4597	
	Archaea		<0.0001	0.7597	
	Bacteria		0.0048	0.3372	

*p*-values determined by Fisher's combined probability test. The rows giving different taxonomic ranges indicate the origin of the sequences that were used to calculate the conservation profiles.

Simple linear regression was used to correlate site conservation with intron/intein penetrance (the number of species infected with a specific element). Significance was then calculated using standard methods based upon the resulting correlation coefficient (*r*), and the degrees of freedom in the sample (*n*-1).

## Authors' contributions

JPG conceived and supervised the study. KSS and AGS generated the conservation profiles and wrote the manuscript. KSS generated the protein mapping and performed all analyses reported in additional file 4. GPF performed the statistical analyses and wrote the methods for the analyses. All authors read and approved the final manuscript.

## Supplementary Material

Additional file 1 **Table of *p*-values and alignment profiles for additional 37 intein, group I or group II host proteins**. Arrows point to intron or intein positions. Blue dots indicate intein positions, green dots group I intron positions, and orange dots group II intron positions. Click here for file

Additional file 2 **List of Introns found in Cytochrome C oxidase subunit I**. The list contains all introns from species for which at least one of their *cox1 *gene introns were BLAST hits when introns from *Podospora anserina *(X55026) and *Saccharomyces cerevisiae *(V00694) *cox1 *genes were used as query sequences. Click here for file

Additional file 3 **Accession numbers for sequences used in protein alignment**. Lists of accession numbers for each protein used for each conservation profile. Click here for file

Additional file 4 **Perl scripts used to calculate the conservation profiles**. Perl scripts used to calculate conservation profiles. Click here for file
