# Supplementary material for: Conservation of intron and intein insertion sites: implications for life histories of parasitic genetic elements
Source: BMC Evol Biol. 2009 Dec 31;9:303. doi: 10.1186/1471-2148-9-303 (PMC2814812; doi:10.1186/1471-2148-9-303)

Protein	Mobile element	Number Positions	*p*-value
ClpP protease	Intein	1	0.0015
DNA gyrase	Intein	2	0.077*
Dna polymerase A	Intein	2	0.0113*
DNA polymerase III	Intein	2	0.0434*
DNA primase/helicase	Intein	1	0.0282
DnaB Helicase	Intein	3	0.0008*
DNA-directed DNA polymerase	Intein	4	<0.0001*
DNA-directed RNA polymerase subunit B	Intein	2	0.0035*
DNA-directed RNA polymerase subunit A	Intein	1	0.0056
FeS assembly protein	Intein	1	0.1745
Filamentous hemagglutinin	Intein	1	0.7893
Glutamate synthase	Intein	1	0.0733
gp51	Intein	1	0.0026
Hypothetical protein UPF0027	Intein	1	0.0998
KlbA	Intein	1	0.0513
Large helicase related protein	Intein	1	0.0041
Lon, ATP-dependent protease LA	Intein	1	0.0613
MupF	Intein	1	0.0014
Phage terminase-like protein	Intein	1	0.0003
Phosphoenolpyruvate synthase	Intein	1	0.0061
pre-mRNA splicing factor PRP8	Intein	1	0.0395
Recombinase A	Intein	3	0.0338*
Reverse gyrase/topoisomerase I	Intein	1	0.1131
Ribonucleotide reductase, Class I + II	Intein	7	<0.0001*
	Group I Intron	1	0.0256
	Group II Intron	3	0.7407*
Ribonucleotide reductase, Anaerobic	Intein	3	<0.0001*
RNA terminal phosphate cyclase	Intein	1	0.1054
SNF2/Rad54 helicase	Intein	1	0.0198
Threonyl-tRNA synthetase	Intein	1	0.0067
Translation initiation factor IF2	Intein	1	0.0127
UDP-glucose 6-dehydrogenase	Intein	1	<0.0001
NADH dehydrogenase subunit 1	Group I Intron	6	0.0979*
	Group II Intron	10	0.9381*
NADH dehydrogenase subunit 2	Group II Intron	1	0.239
NADH dehydrogenase subunit 3	Group I Intron	1	0.7846
	Group II Intron	1	0.2886
NADH dehydrogenase subunit 5	Group I Intron	7	0.0042*
	Group II Intron	2	0.1627*
Chloroplast psbA	Group I Intron	7	0.0111*
Chlorophyll alpha apoprotein A2	Group I Intron	1	0.4477
Chlorophyll a binding protein	Group I Intron	2	0.074*

* *p*-values determined by Fisher's combined probability method

ClpP Protease

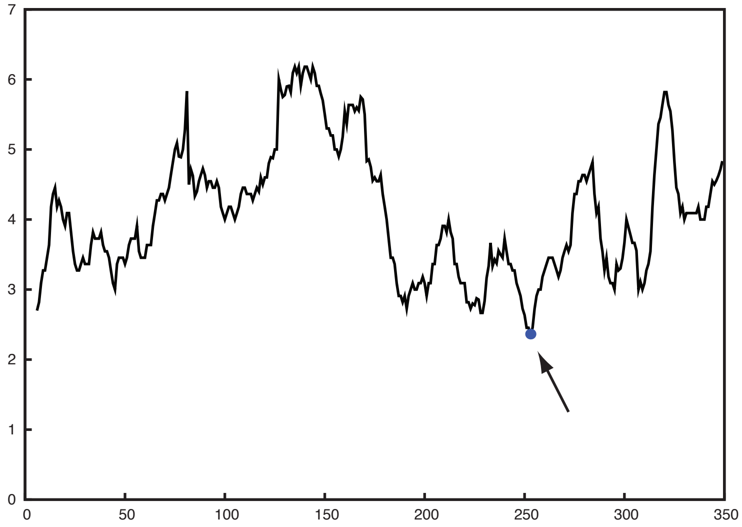

DNA Gyrase

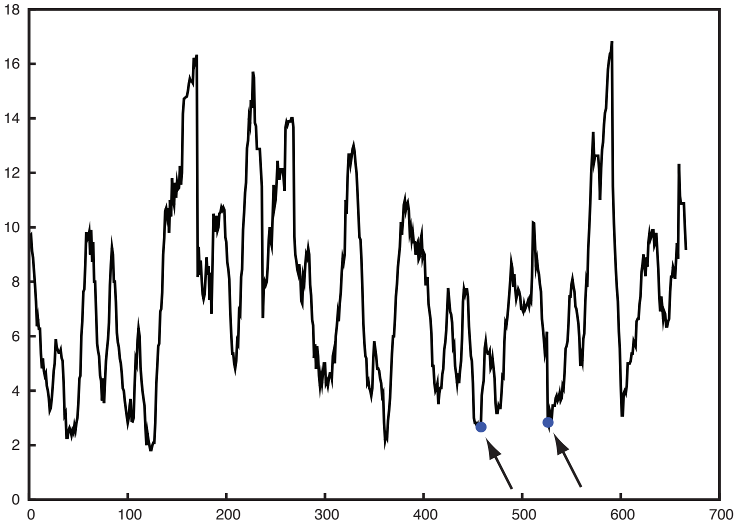

DNA polymerase A

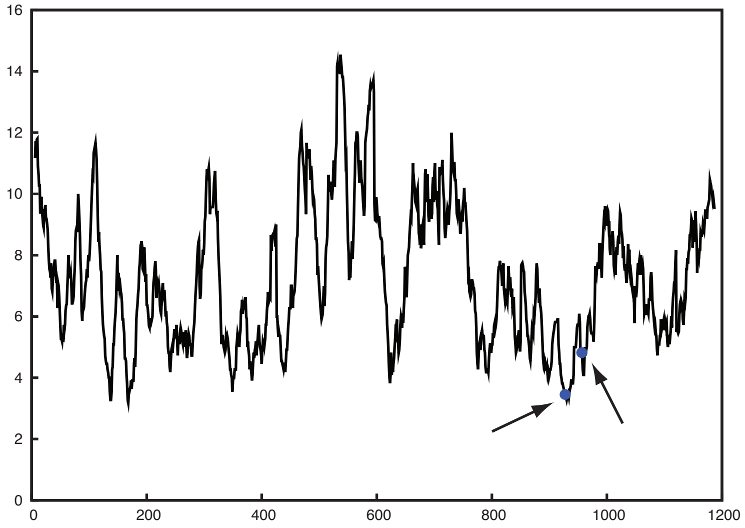

DNA Polymerase III

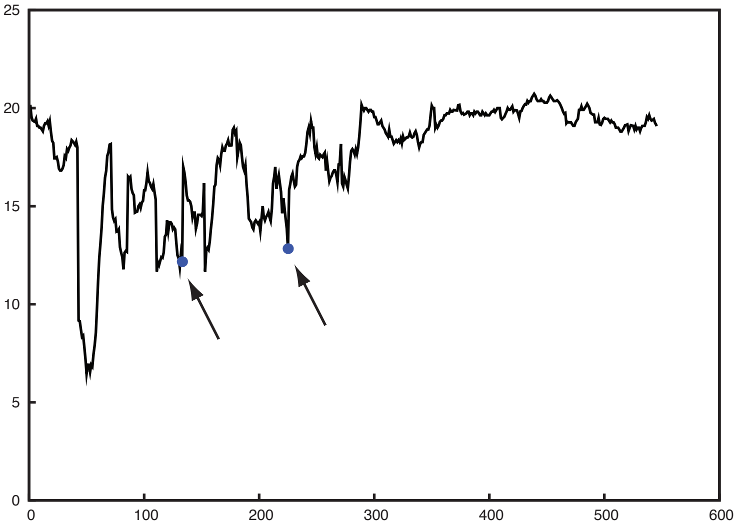

DNA primase/helicase

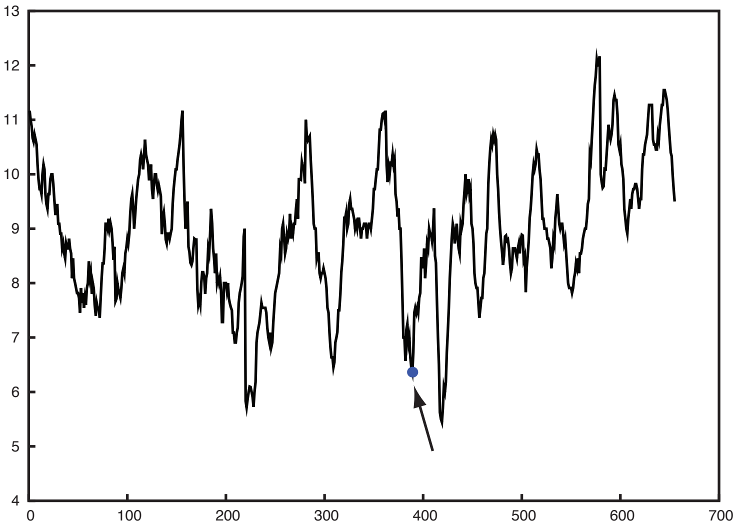

DnaB helicase

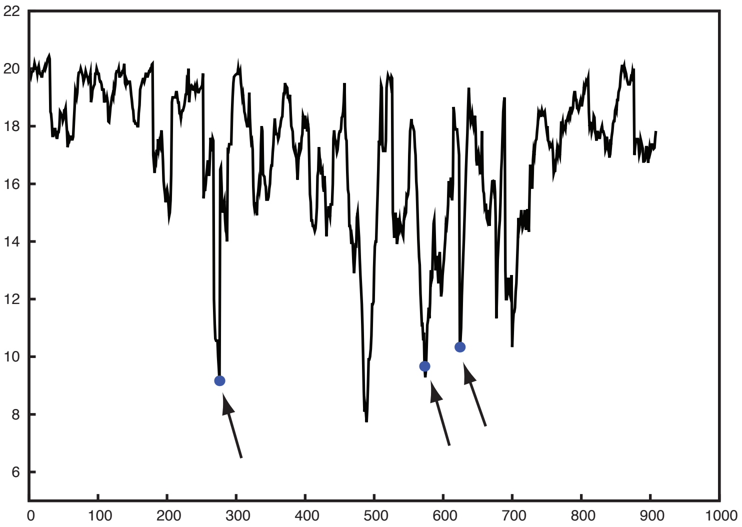

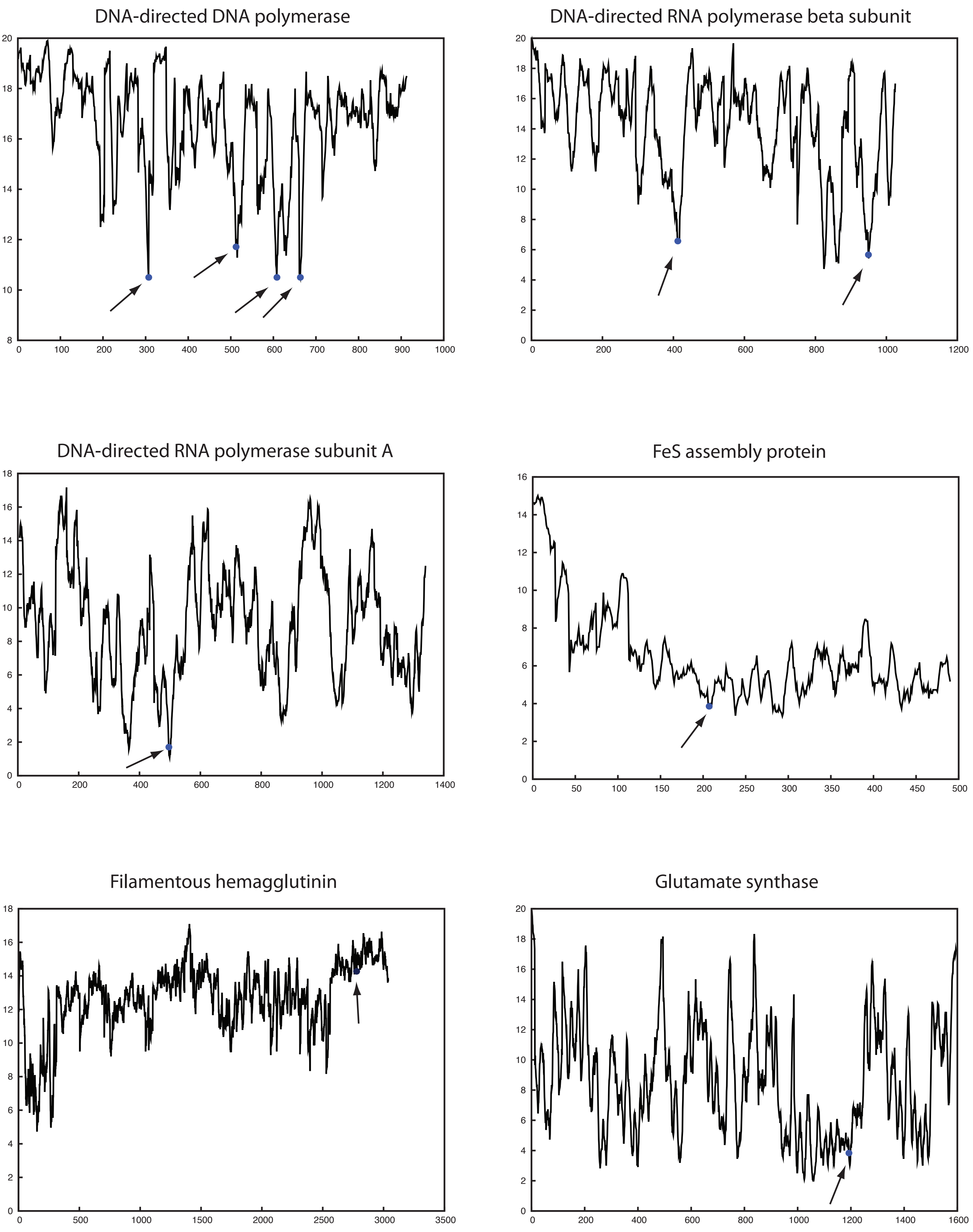
DNA-directed DNA polymerase
DNA-directed RNA polymerase beta subunit
DNA-directed RNA polymerase subunit A
FeS assembly protein
Filamentous hemagglutinin
Glutamate synthase

gp51

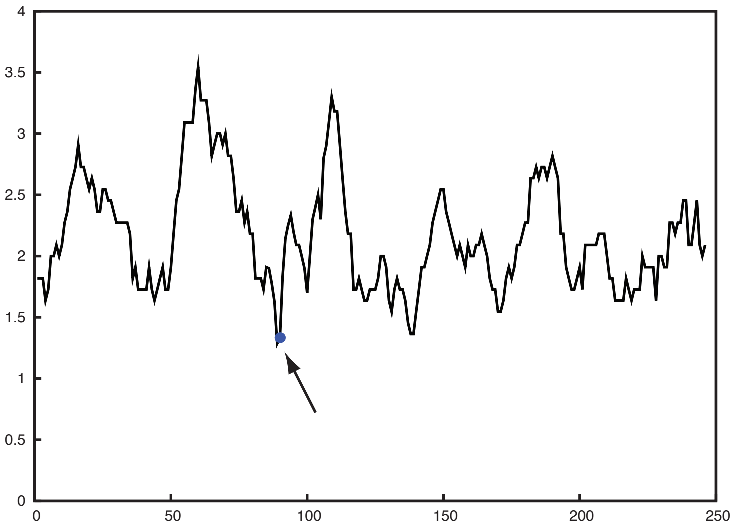

Hypothetical protein UPF0027

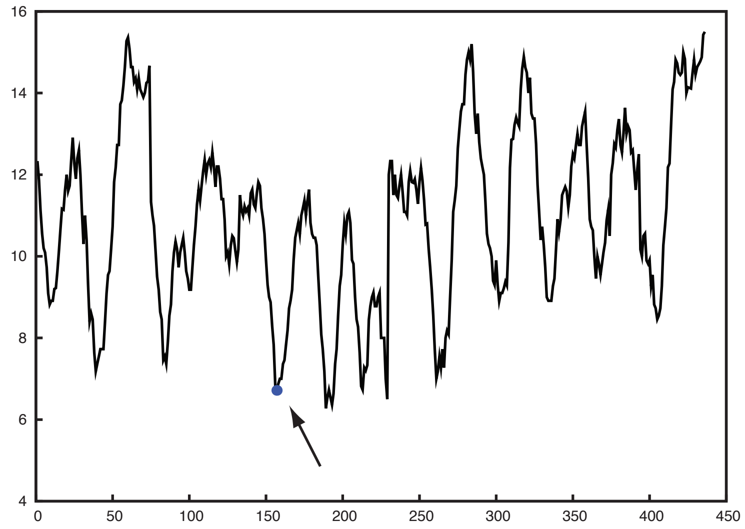

KlbA

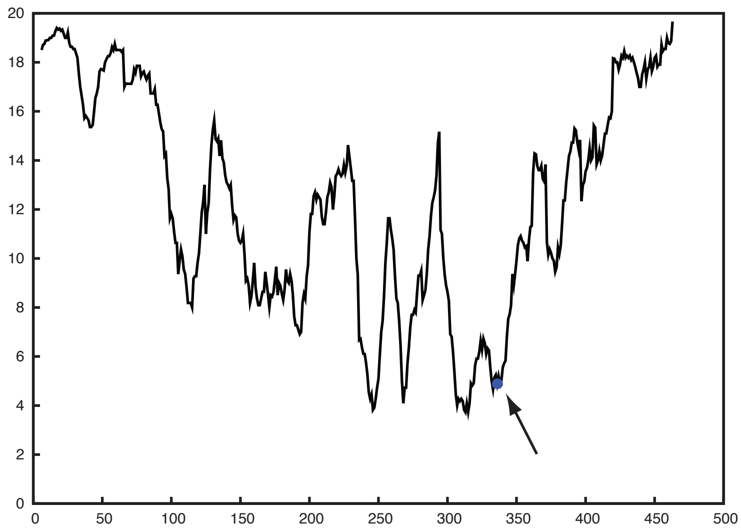

Large Helicase Related Protein

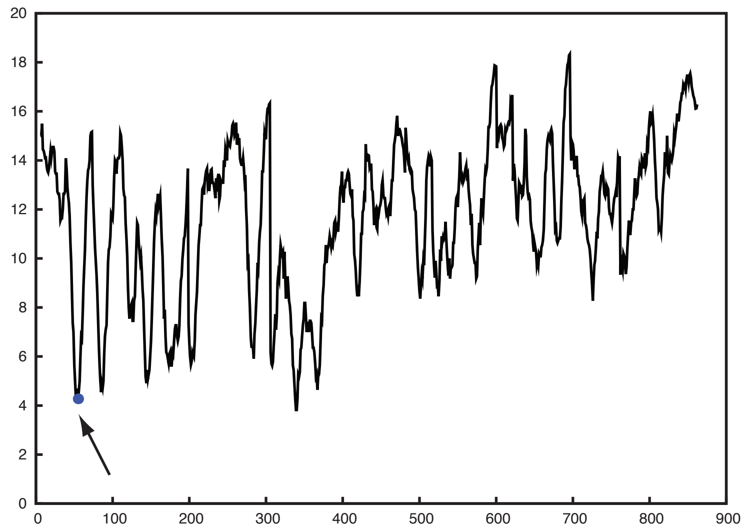

Lon, ATP-dependent protease LA

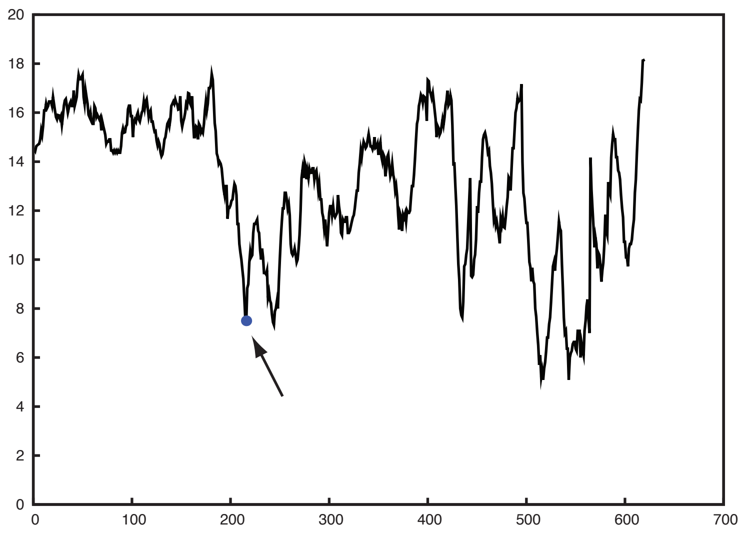

MupF

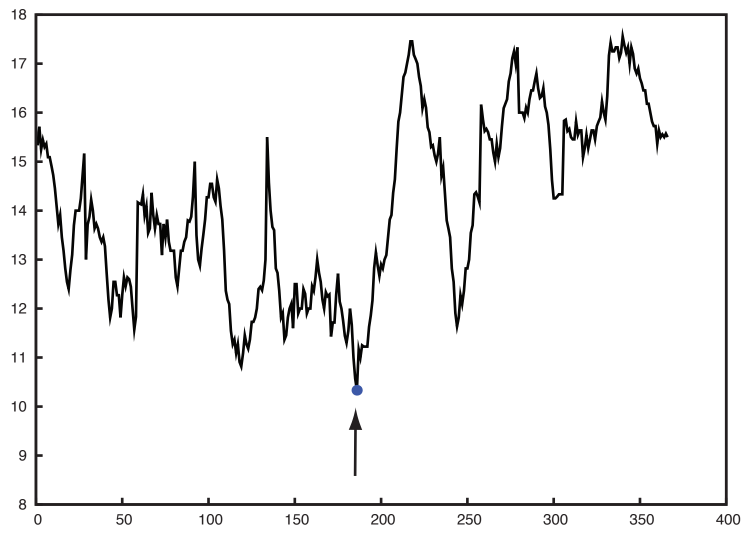

Phage terminase-like protein

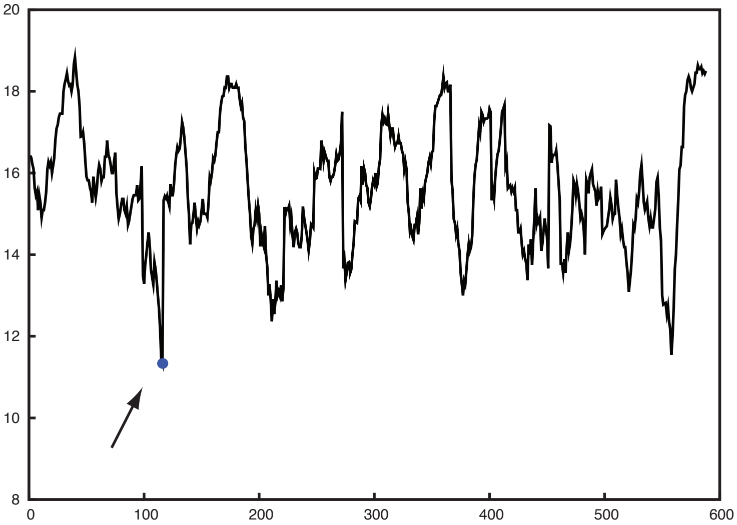

Phosphoenolpyruvate Synthase

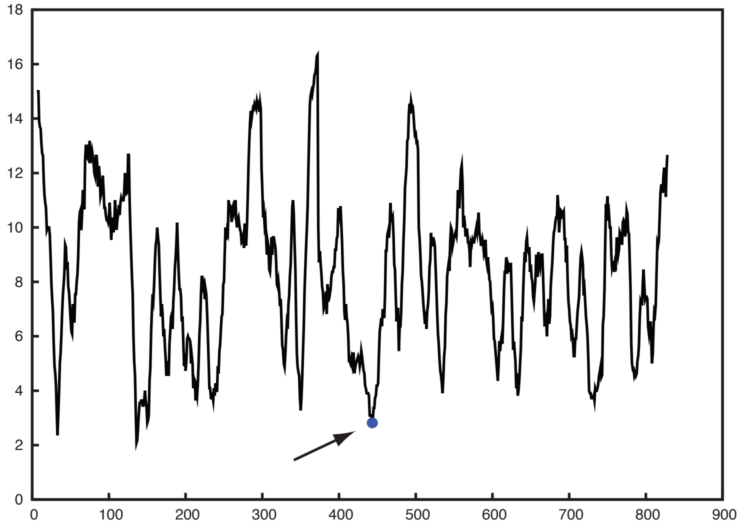

Pre-mRNA Splicing Factor PRP8

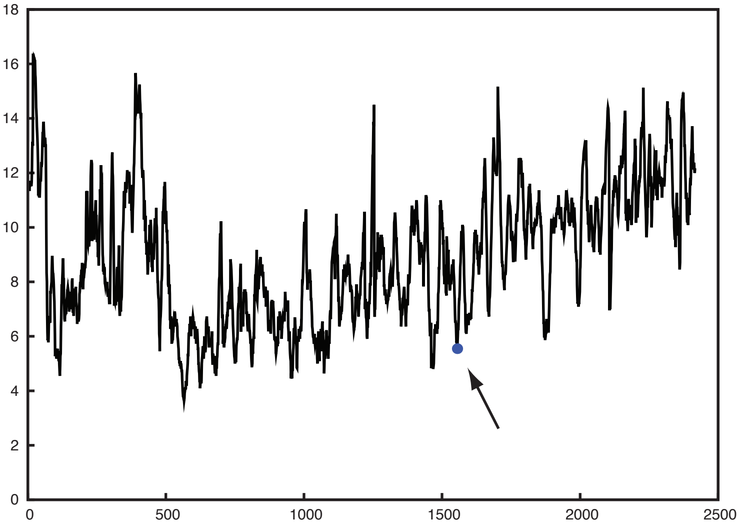

Recombinase A

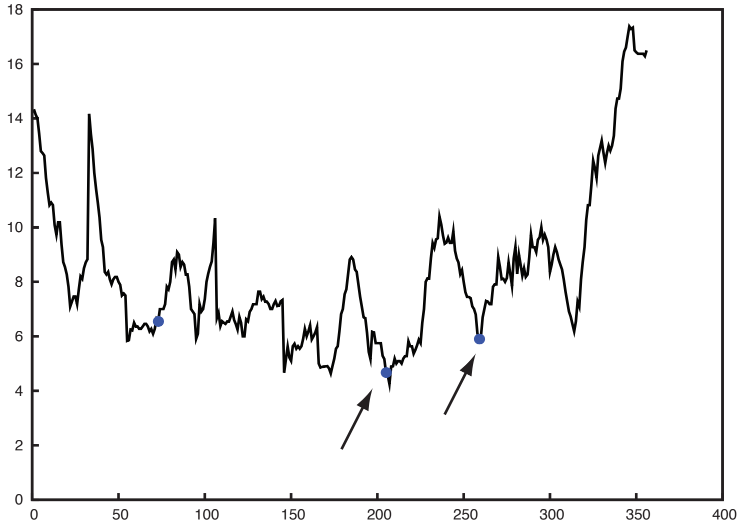

Reverse Gyrase/Topoisomerase I

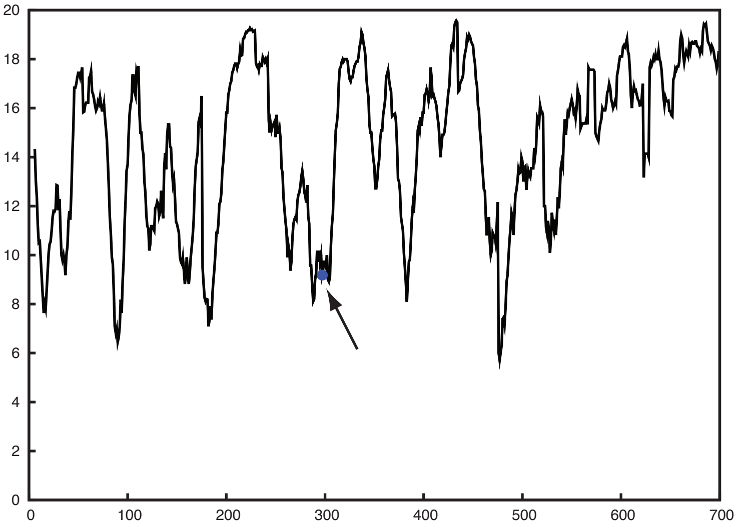

Ribonucleotide reductase, class I + II

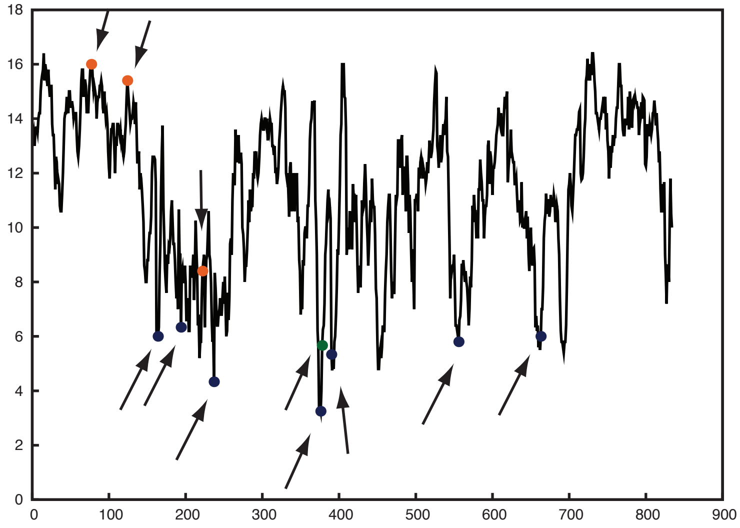

Ribonucleotide reductase, Anaerobic

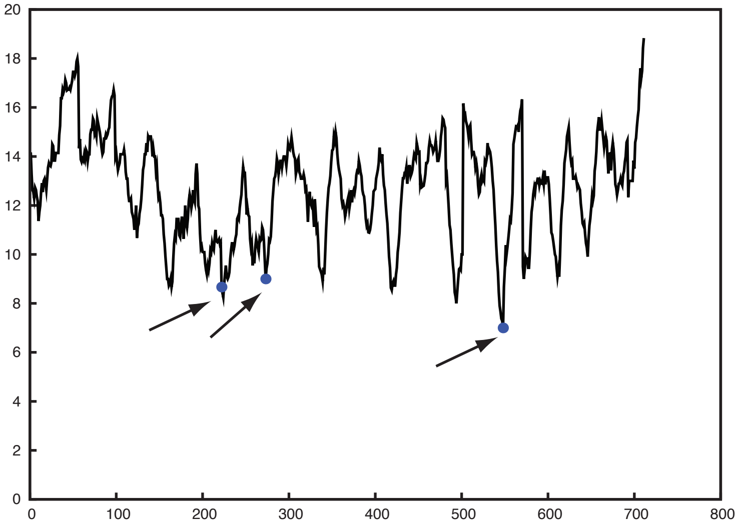

RNA terminal phosphate cyclase

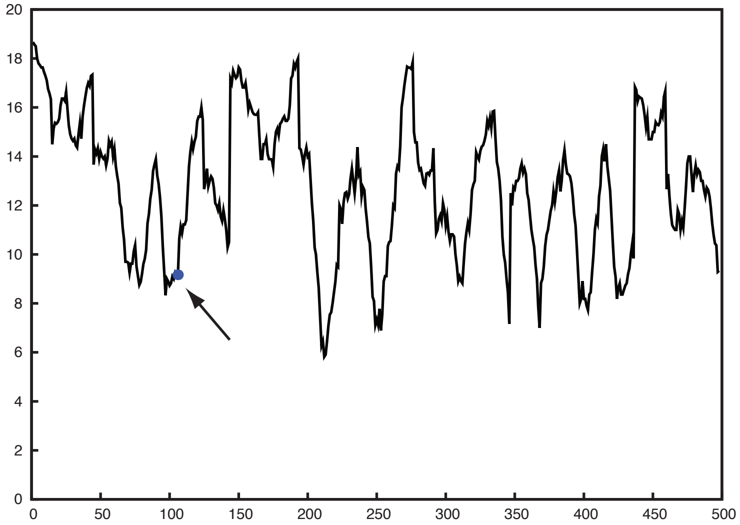

SNF2/Rad54 helicase

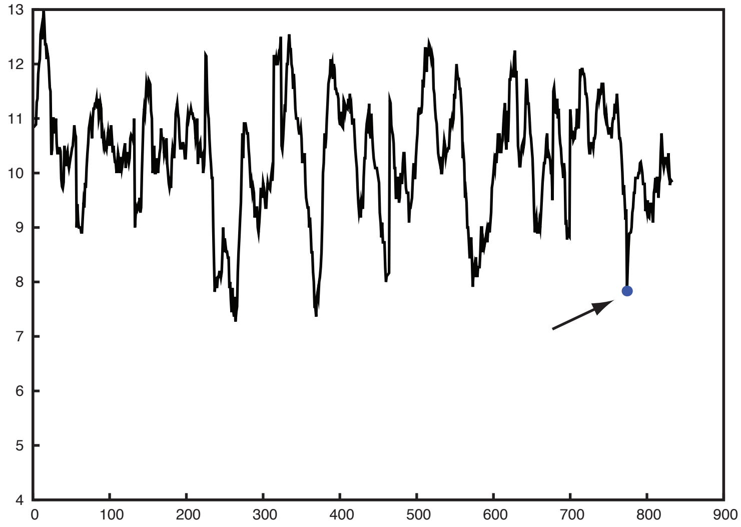

Threonyl-tRNA synthetase

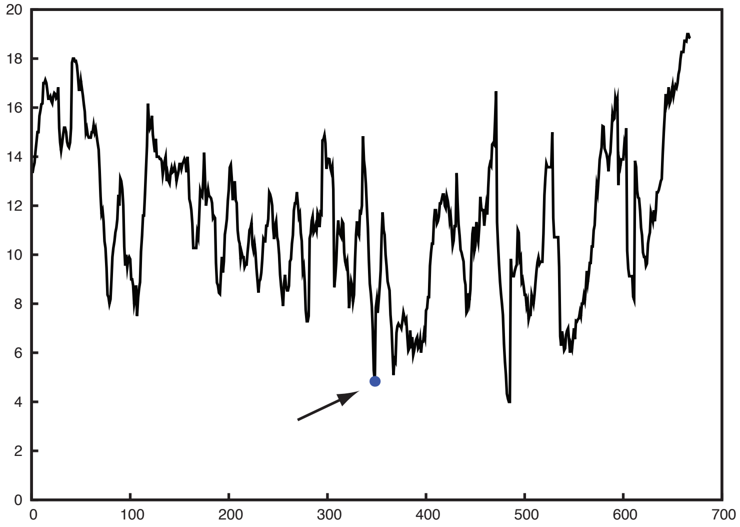

Translation initiation factor IF2

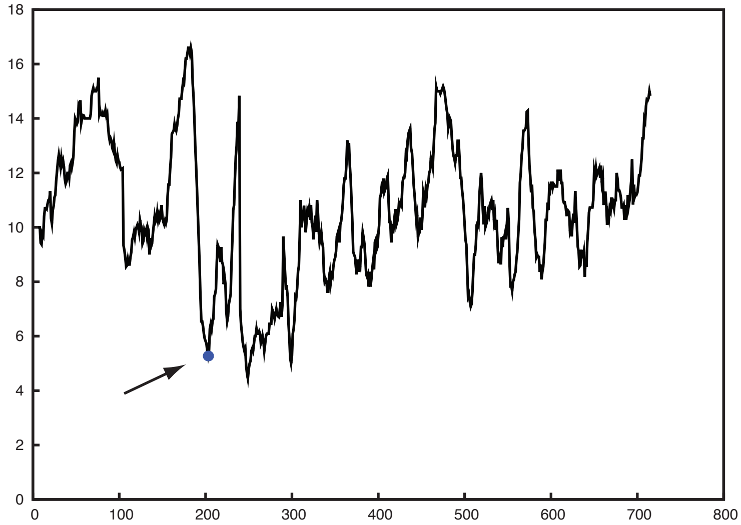

UDP-glucose 6-dehydrogenase

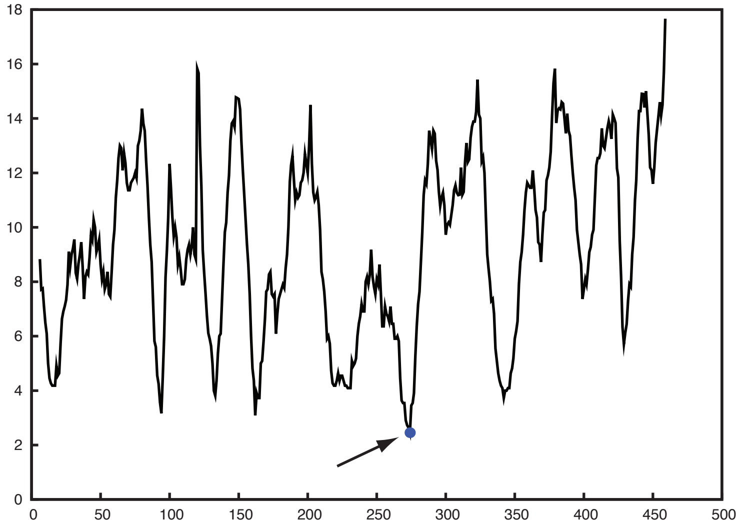

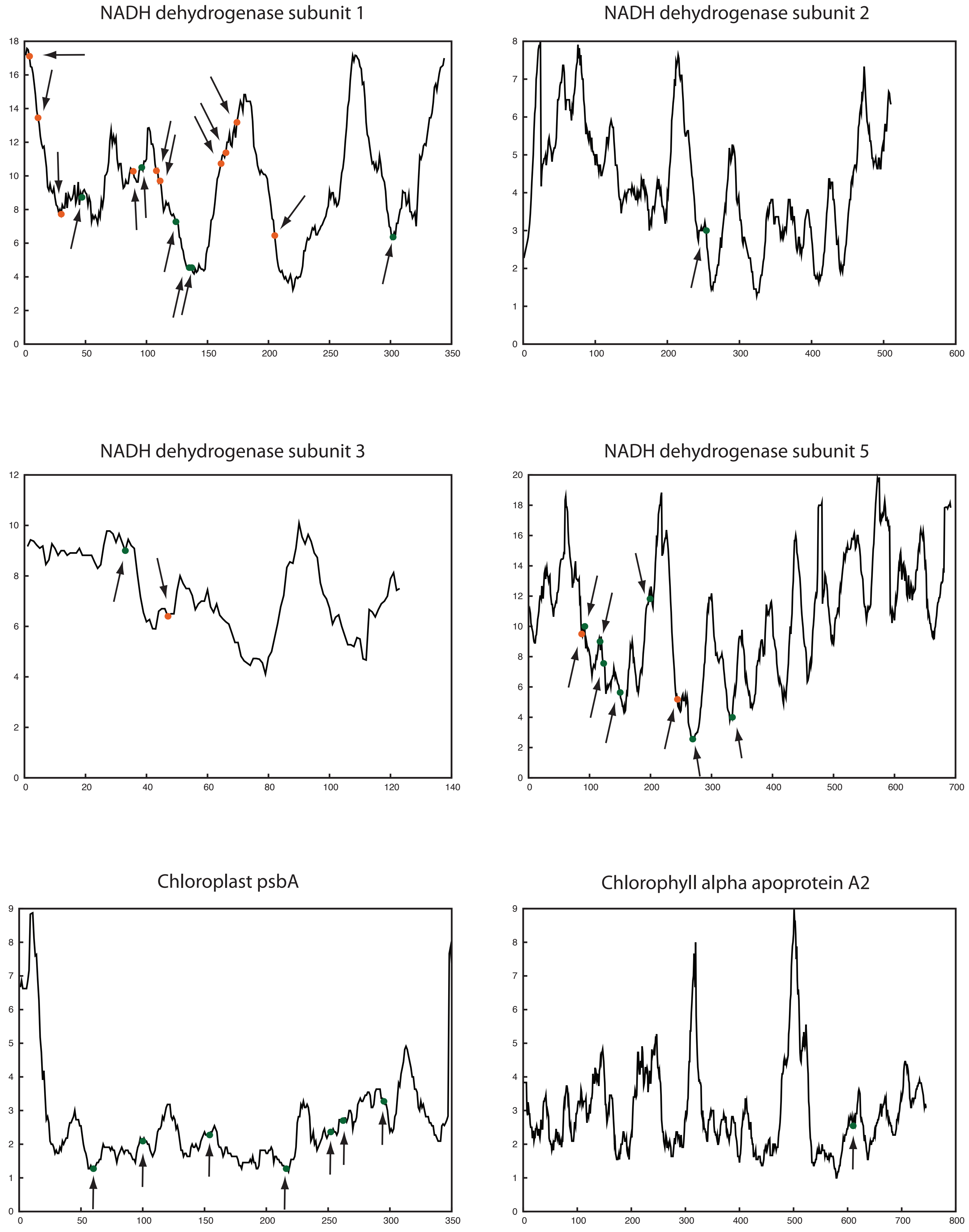
NADH dehydrogenase subunit 1
NADH dehydrogenase subunit 2
NADH dehydrogenase subunit 3
NADH dehydrogenase subunit 5
Chloroplast psbA
Chlorophyll alpha apoprotein A2

Chlorophyll a binding protein

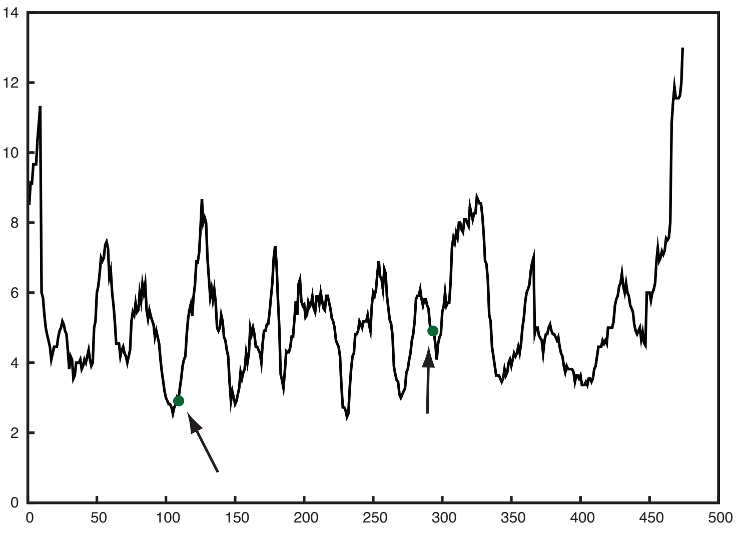

Supplement: Additional file 1 — Table of p-values and alignment profiles for additional 37 intein, group I or group II host proteins. Arrows point to intron or intein positions. Blue dots indicate intein positions, green dots group I intron positions, and orange dots group II intron positions. [file 1471-2148-9-303-S1.PDF]
