# Supplementary material for: Conservation of intron and intein insertion sites: implications for life histories of parasitic genetic elements
Source: BMC Evol Biol. 2009 Dec 31;9:303. doi: 10.1186/1471-2148-9-303 (PMC2814812; doi:10.1186/1471-2148-9-303)
Supplement: Additional file 2 — List of Introns found in Cytochrome C oxidase subunit I. The list contains all introns from species for which at least one of their cox1 gene introns were BLAST hits when introns from Podospora anserina (X55026) and Saccharomyces cerevisiae (V00694) cox1 genes were used as query sequences. [file 1471-2148-9-303-S2.PDF]

List of Introns found in Cytochrome C oxidase subunit I.

The list contains all introns from species for which at least one of their *cox1* gene introns were BLAST hits when introns from *Podospora anserina* (X55026) and *Saccharomyces cerevisiae* (V00694) *cox1* genes were used as query sequences (searches performed February 5-15th, 2007).
The following three BLAST programs were used: Protein-protein BLAST (blastp), Nucleotide-nucleotideBLAST (blastn), and Translated query vs. protein database (blastx). Colors reflect taxonomy[$]. Species are indicated as follows: *Agrocybe aegerita* (Aae), *Allomyces macrogynus* [7], *Amoebidium parasiticum* (Apa), *Candida parapsilosis* (Cpa), *Candida glabrata* (Cgl), *Candida stellata* (Cst), *Candida orthopsilosis* [17], *Chara vulgaris* (Cvu), *Chaetosphaeridium globosum* (Cgl2), *Dictyostelium discoideum* (Ddi), *Dictyostelium citrinum* (Dci), *Emericella nidulans* (Eni), *Epidermophyton floccosum* (Efl), *Hanseniaspora uvarum* (Huv), *Hypocrea jecorina* (Hje), *Kluyveromyces lactis* (Kla), *Kluyveromyces thermotolerans* (Kth), *Marchantia polymorpha* (Mpo), *Mesostigma viride* (Mvi), *Metridium senile* (Mse), *Monoblepharella* sp. JEL15 (Msp), *Moniliophthora perniciosa* (Mpe), *Mortierella verticillata* (Mve), *Monosiga brevicollis* [12], *Neurospora crassa* (Ncr), *Oltmannsiellopsis viridis* (Ovi), *Penicillium marneffei* [48], *Pellia epiphylla* (Pep), *Pichia Canadensis* (Pca), *Podospora anserina* [10], *Prototheca wickerhamii* (Pwi), *Pseudendoclonium akinetum* (Pak), *Pylaiella littoralis* (Pli), *Rhizophydium* sp. 136 (Rsp), *Rhizopus oryzae* (Ror), *Rhodomonas salina* (Rsa), *Saccharomyces cerevisiae* (Sce), *Saccharomyces servazzii* (Sse), *Scizosaccharomyces octosporus* (Soc), *Schizosaccharomyces japonicus* (Sja), *Schizosaccharomyces pombe* [17], *Smittium culisetae* (Scu), *Spizellomyces punctatus* (Spu), *Tetilla* sp. SP25456 (Tsp), *Thalassiosira pseudonana* (Tps), *Thalassiosira nordenskioeldii* (Tno), *Ustilago maydis* (Uma), *Yarrowia lipolytica* (Yli).

Position	Intron Group	Conserve Domain/s	Host
1	II	COG3344, RVT, X	Mpo, Pep
2	II	COG3344, RVT, X	Pan
3	II	COG3344, RVT, X	Kth, Sce, Mpo, Pep
4	II<br>I	COG3344, X<br>N/A	Kla, Ncr, Sce, Cvu, Rsa<br>Rsp
5	I<br>I	GIY-YIG, IENR1<br>N/A	Msp, Mve, Yli<br>Ddi, Dci
6	I	N/A	Ama
7	I	N/A	Rsp
8	I	2 x LAGLIDADG	Ama, Cpa, Cgl, Kla, Kth, Pan, Rsp, Ror, Sce, Sja, Yli
9	I	2 x LAGLIDADG	Ama, Eni, Ncr, Pan, Pma, Rsp, Uma, Apa, Cgl2
10	II	COG3344, RVT, X, HNH	Pan
11	I	N/A	Ama, Mpo, Pep, Apa
12	I	2 x LAGLIDADG	Aae, Ama, Cgl, Cst, Hje, Kth, Mpe, Pma, Rsp, Ror, Sce, SSe, Soc, Spo, Uma, Yli, Cgl2, Mpo, Pep, Pwi, Pak, Apa, Mbr
13	I	2 x LAGLIDADG	Pan
14		LAGLIDADG	Spu
15	I	GIY-YIG, IENR1<br>N/A	Pan, Uma<br>Mpo, Pep
16	I	LAGLIDADG	Rsp
17	I	2 x LAGLIDADG	Ama, Eni, Msp, Mpe, Ncr, Pan, Ror, Scu, Uma, Mpo, Pep, AmApa
18	I	LAGLIDADG	Ama
19	I	2 x LAGLIDADG	Cpa, Cst, Kla, Mve, Pan, Rsp, Sce, Scu, Uma, Pak, Pwi
20	I	2 x LAGLIDADG	Cpa, Cst, Huv, Kth, Msp, Pca, Pan, Rsp, Ror, Sce, Soc, Sse, Scu, Yli, Cvu, Mpo, Pep, Pwi, Ddi, Dci, Mbr (plus over 48 Plants)
21	I	1-2 x LAGLIDADG	Aae, Eni, Mpe, Ncr, Pma, Soc, Sja, Spo, Scu, Yli, Cvu, Pak
22	II	COG3344, RVT, X	Pli, Ovi, Rsa
23	I<br>I	2 x LAGLIDADG<br>N/A	Pan<br>Cgl2, Mbr
24	I<br>II	2 x LAGLIDADG<br>COG3344, RVT, X	Rsp<br>Tno, Pli, Cvu
25	I	1 x LAGLIDADG	Efl, Pan
26	I	2 x LAGLIDADG	Ama, Cpa, Efl, Hje, Mpe, Pma, Pan, Rsp, Scu, Uma, Yli, Cvu, Cgl2, Mvi, Ddi, Dci, Apa
27	I	2 x LAGLIDADG	Mse (plus over 29 Cnidaria)
28	I	1-2 x LAGLIDADG	Cst, Mpe, Pan, Scu, Cvu, Cgl2
29	II	LAGLIDADG	Rsp, Apa
30	II	COG3344, RVT, X	Soc
31	I	2 x LAGLIDADG	Aae, Cgl, Kla, Sce, Uma, Apa
32	I	N/A	Ama, Mve, Rsp
33	I<br>I	GIY-YIG, IENR1<br>N/A	Aae, Hje, Pma, Pan<br>Cor
34	I	1-2 x LAGLIDADG	Ama, Kla, Msp, Pma, Pan, Rsp, Sce, Sse, Soc, Scu, Uma, Yli, Mpo, Mvi, Pep, Pak, Ddi, Dci, Apa
35	I<br>I	N/A<br>LAGLIDADG	Pan, Rsp, Yli, Apa<br>Cst, Hje
36	II	COG3344, RVT, X	Sce, Pli, Tps
37	I	2 x LAGLIDADG	Spu
38	I	N/A	Ama
39	I	LAGLIDADG	Scu
40	I	GIY-YIG	Ama, Yli
41	I	GIY-YIG	Scu
42	I	GIY-YIG	Mpe

$ Taxonomic affiliation according to the NCBI's Taxonomy database

Eukaryota; Fungi; Ascomycota; Pezizomycotina; Eurotiomycetes; Eurotiales; Trichocomaceae; Emericella
Eukaryota; Fungi; Ascomycota; Pezizomycotina; Eurotiomycetes; Eurotiales; Trichocomaceae; mitosporic Trichocomaceae; Penicillium
Eukaryota; Fungi; Ascomycota; Pezizomycotina; Eurotiomycetes; Onygenales; Arthrodermataceae; mitosporic Arthrodermataceae; Epidermophyton
Eukaryota; Fungi; Ascomycota; Pezizomycotina; Sordariomycetes; Hypocreomycetidae; Hypocreales; Hypocreaceae; Hypocrea.
Eukaryota; Fungi; Ascomycota; Pezizomycotina; Sordariomycetes; Sordariomycetidae; Sordariales; Lasiosphaeriaceae; Podospora
Eukaryota; Fungi; Ascomycota; Pezizomycotina; Sordariomycetes; Sordariomycetidae; Sordariales; Sordariaceae; Neurospora
Eukaryota; Fungi; Ascomycota; Saccharomycotina; Saccharomycetes; Saccharomycetales; mitosporic Saccharomycetales; Candida.
Eukaryota; Fungi; Ascomycota; Saccharomycotina; Saccharomycetes; Saccharomycetales; Saccharomycodaceae; Hanseniaspora
Eukaryota; Fungi; Ascomycota; Saccharomycotina; Saccharomycetes; Saccharomycetales; Saccharomycetaceae; Kluyveromyces.
Eukaryota; Fungi; Ascomycota; Saccharomycotina; Saccharomycetes; Saccharomycetales; Saccharomycetaceae; Pichia.
Eukaryota; Fungi; Ascomycota; Saccharomycotina; Saccharomycetes; Saccharomycetales; Saccharomycetaceae; Saccharomyces.
Eukaryota; Fungi; Ascomycota; Saccharomycotina; Saccharomycetes; Saccharomycetales; Dipodascaceae; Yarrowia
Eukaryota; Fungi; Ascomycota; Schizosaccharomycetes;Schizosaccharomycetales; Schizosaccharomycetaceae; Schizosaccharomyces
Eukaryota; Fungi; Basidiomycota; Hymenomycetes; Homobasidiomycetes; Agaricales; Bolbitiaceae; Agrocybe
Eukaryota; Fungi; Basidiomycota; Hymenomycetes; Homobasidiomycetes; Agaricales; Tricholomataceae; mitosporic Tricholomataceae; Moniliophthora
Eukaryota; Fungi; Basidiomycota; Ustilaginomycetes; Ustilaginomycetidae; Ustilaginales; Ustilaginaceae; Ustilago
Eukaryota; Fungi; Chytridiomycota; Blastocladiales; Blastocladiaceae; Allomyces
Eukaryota; Fungi; Chytridiomycota; Chytridiales; Chytridiaceae; Rhizophydium
Eukaryota; Fungi; Chytridiomycota; Monoblepharidales; Monoblepharidaceae; Monoblepharella
Eukaryota; Fungi; Chytridiomycota; Spizellomycetales; Spizellomycetaceae; Spizellomyces
Eukaryota; Fungi; Zygomycota; Zygomycetes; Mucorales; Mucoraceae; Rhizopus
Eukaryota; Fungi; Zygomycota; Zygomycetes; Mortierellales; Mortierellaceae; Mortierella
Eukaryota; Fungi; Zygomycota; Trichomycetes; Harpellales; Legeriomycetaceae; Smittium

Eukaryota; Viridiplantae; Chlorophyta; Oltmannsiellopsis
Eukaryota; Viridiplantae; Chlorophyta; Trebouxiophyceae; Chlorellales; Chlorellaceae; Prototheca
Eukaryota; Viridiplantae; Chlorophyta; Ulvophyceae; Ulvales; Pseudendoclonium
Eukaryota; Viridiplantae; Streptophyta; Charophyceae; Charales; Characeae; Chara
Eukaryota; Viridiplantae; Streptophyta; Coleochaetophyceae; Coleochaetales; Chaetosphaeridiaceae; Chaetosphaeridium
Eukaryota; Viridiplantae; Streptophyta; Embryophyta;Marchantiophyta; Superclass II; Marchantiopsida; Marchantiidae; Marchantiales; Marchantiaceae; Marchantia
Eukaryota; Viridiplantae; Streptophyta; Embryophyta; Marchantiophyta; Superclass III; Pelliopsida; Pelliales; Pelliaceae; Pellia
Eukaryota; Viridiplantae; Streptophyta; Mesostigmatophyceae; Mesostigmatales; Mesostigmataceae; Mesostigma

Eukaryota; Metazoa; Cnidaria; Anthozoa; Hexacorallia; Actiniaria; Nynantheae; Metridiidae; Metridium
Eukaryota; Metazoa; Porifera; Demospongiae; Tetractinomorpha; Spirophorida; Tetillidae; Tetill

Eukaryota; stramenopiles; Bacillariophyta; Coscinodiscophyceae; Thalassiosirophycidae; Thalassiosirales; Thalassiosiraceae; Thalassiosira.
