# Supplementary material for: Conservation of intron and intein insertion sites: implications for life histories of parasitic genetic elements
Source: BMC Evol Biol. 2009 Dec 31;9:303. doi: 10.1186/1471-2148-9-303 (PMC2814812; doi:10.1186/1471-2148-9-303)
Supplement: Additional file 3 — Accession numbers for sequences used in protein alignment. Lists of accession numbers for each protein used for each conservation profile. [file 1471-2148-9-303-S3.DOC]

**Accession numbers for protein sequences used in the alignments.**

**Archaeal/vacuolar-type ATPase catalytic subunit A proteins:** 1VDZ_A, A56812, AAA82881, AAA21531, AAA30392, AAA33050, AAA33139, AAA61760, AAA72192, AAA82881, AAA83249, AAA85820, AAB40515, AAB50981, AAC06375, AAC17840, AAC49174, AAC59680, AAL90250, AAP37742, BAA09098, BAA36691, CAA45537, CAA49775, CAA67305, CAA98761, CAB51771, CAB52268, CAB55557, CAB99208, EAA32337, EAL02842, NP_069995, NP_127437, NP_218969, NP_247186, NP_276090, NP_294423, NP_342089, O06504, O32466, P22662, Q08636, Q26975, Q38676, Q48332, XP_001321627, XP_001349847

**Replication factor C proteins:** 1IQP_A, 2CHV_A, AAB09786, AAB60241, AAB84747, AAB88360, AAG37985, AAG51618, AAK41065, AAP36459, BAA29181, BAA80521, BAA82745, BAB27561, BAB60660, CAA07618, CAC12618, NP_002905, NP_014547, NP_070884, NP_082404, NP_125803, NP_275384, O94449, P34429, P53033, Q9HN27.

**Cell division control protein 21 proteins:** 2115257A, AAB35644, AAB86236, AAB94861, AAC60223, AAF17244, AAG37990, AAN73053, AAS83108, BAA29695, BAA79100, BAB27813, BAB60203, CAA19452, CAA55125, CAA72333, CAA82556, CAA90164, CAB58403, CAC36296, CAE05930, CAJ02400, NP_058983, NP_127115, NP_175112 , NP_280836, NP_394261, NP_588004, Q24849, Q9UXG1

**DNA polymerase I proteins:** 1KLN_A, AAS09424, CAK24997, CAK25954, EAL73185, NP_220007, NP_229419, NP_290488, NP_562910, NP_603602, NP_782664, P30314, XP_729420, YP_188827, YP_495560, YP_515795, YP_676477, YP_690972, YP_765765, YP_899218, YP_950517, ZP_00143567, ZP_00952553, ZP_01003352, ZP_01228658

**Cytochrome C Oxidase Subunit I proteins:** 803222A, 1OCC A, AAK14378, AAO70008, CAA32799, CAA38777, NP_008083, NP_008278, NP_009305, NP_038208, NP_042244, NP_150115, NP_258414, NP_279674, NP_389373, NP_414965, NP_700367, NP_818778, YP_024082, YP_135922, YP_203292, YP_246643, YP_406866, YP_588386, YP_639017, YP_667836, YP_783954, ZP_00373550, ZP_00629671

**ClpP protease:** YP_635990.1, YP_635828.1, YP_764387.1, P42379.1, ABU88229.1, ABU88294.1, ZP_02179372.1, ACI31244.1, ACJ50097.1, YP_002600892.1, YP_002601006.1, NP_958364.1, NP_488397.1, YP_001516654.1, BAC08624.1, YP_321824.1, EEG03687.1

**Translation initiation factor IF2:** YP_658772.1, NP_050746.1, ZP_01452543.1, NP_069602.1, CAL53117.1, YP_806789.1, YP_826419.1, YP_843720.1, YP_867618.1, YP_919909.1, YP_931097.1, 1G7R, YP_999860.1, YP_001012654.1, P18311.1, YP_001030951.1, YP_001046376.1, YP_001055530.1, YP_001040838.1, YP_001097901.1, YP_001125236.1, NP_126820.1, YP_001152871.1, NP_143000.1, NP_148571.1, YP_001180846.1, YP_001190131.1, YP_684480.1, YP_001228226.1, YP_001272775.1, ZP_01809991.1, ZP_01817253.1, ZP_01821328.1, ZP_01822057.1, ZP_01827169.1, ZP_01829405.1, ZP_01835566.1, ZP_01913353.1, ZP_01923079.1, YP_001323805.1, YP_001325094.1, YP_001330501.1, YP_001355892.1, YP_001378333.1, YP_001397395.1, YP_001405451.1, YP_001408785.1, NP_214394.1, NP_247234.1, NP_275402.1, YP_001434592.1, YP_001481705.1, YP_001486808.1, NP_280688.1, YP_001513067.1, NP_341780.1, NP_345072.1, NP_358075.1, YP_001540292.1, YP_001551501.1, YP_001548718.1, YP_001616441.1, ZP_02176703.1, ZP_02193283.1, YP_001608710.1, ZP_02710175.1, YP_001694043.1, YP_001737449.1, YP_001794016.1, ZP_02913017.1, YP_001814306.1, AAB41057.1, NP_559347.1, Q18FT0.2, NP_578866.1, YP_001987724.1, ZP_03053583.1, YP_002037217.1, YP_002048767.1, YP_002121676.1, ZP_03146295.1, ZP_03210529.1, NP_616459.1, NP_614878.1, ZP_03223273.1, YP_002250932.1, NP_623011.1, XP_002140628.1, YP_002307333.1, NP_634487.1, EEB18236.1, YP_002316041.1, YP_002353101.1, YP_002343596.1, YP_002429042.1, YP_002465444.1, ZP_03557576.1, YP_002510541.1, YP_002566708.1, YP_002574836.1, YP_002577718.1, YP_002577767.1, YP_002581198.1, YP_002582415.1, YP_002595381.1, YP_002598927.1, XP_002291169.1, YP_002607932.1, ZP_03662044.1, ZP_03693014.1, YP_002711950.1, YP_002728188.1, ZP_03844620.1, ZP_03869473.1, ZP_03874773.1, ZP_03964983.1, ZP_03981802.1, ZP_03999039.1, ZP_04012659.1, ZP_04019061.1, ZP_04039007.1, ZP_04050553.1, ZP_04434774.1, ZP_04441141.1, YP_002887315.1, NP_720869.1, NP_785565.1, NP_814997.1, BAA35081.1, NP_963777.1, NP_987404.1, YP_075351.1, BAD53222.1, YP_137613.1, YP_147116.1, YP_172785.1, YP_178155.1, YP_183718.1, YP_191979.1, YP_255368.1, XP_764745.1, YP_263377.1, YP_301589.1, YP_306837.1, YP_331215.1, YP_398083.1, YP_395859.1, YP_412551.1, YP_447670.1, CAJ57174.1, XP_952541.1, ZP_01067767.1, ZP_01069641.1, ZP_01071814.1, ZP_01100613.1, YP_503908.1, ZP_01174024.1, YP_564983.1, YP_579342.1

**KlbA:** AAD38172.2, YP_617332.1, YP_606400.1, YP_620923.1, YP_625337.1, NP_052160.1, YP_673169.1, YP_676532.1, AAG33866.1, YP_725227.1, YP_725494.1, YP_742992.1, YP_755653.1, ZP_01444122.1, YP_759872.1, YP_761688.1, NP_069174.1, NP_069493.1, NP_069829.1, YP_773297.1, YP_778176.1, YP_782663.1, YP_792647.1, YP_765822.1, YP_822690.1, YP_825409.1, YP_832404.1, YP_832412.1, YP_728353.1, YP_845622.1, YP_855994.1, NP_148459.2, ZP_01547111.1, YP_924305.1, YP_919851.1, YP_926586.1, YP_948621.1, ZP_01666173.1, YP_993003.1, YP_995056.1, YP_001013287.1, YP_001030968.1, YP_001037764.1, YP_001047911.1, YP_001058911.1, YP_001064082.1, YP_001066153.1, YP_001076995.1, YP_001042442.1, YP_001041468.1, ZP_01741815.1, ZP_01744909.1, ZP_01749323.1, YP_001092775.1, YP_001098151.1, 2OAP, ZP_01768531.1, ZP_01769062.1, YP_001115620.1, YP_001119332.1, YP_001113458.1, NP_106984.1, CAM74078.1, NP_127065.1, YP_001130089.1, YP_001142666.1, NP_142607.1, YP_001166517.1, YP_001203168.1, YP_001212001.1, YP_686662.1, YP_684939.1, YP_001237640.1, YP_001242823.1, YP_001235137.1, YP_001261134.1, YP_001273170.1, YP_001275628.1, YP_001276215.1, ZP_01813028.1, ZP_01853908.1, ZP_01864251.1, ZP_01877825.1, ZP_01900021.1, ZP_01904165.1, YP_001313832.1, ZP_01924529.1, YP_001329011.1, YP_001323552.1, YP_001325343.1, YP_001330254.1, 2JMZ, YP_001355004.1, YP_001350205.1, YP_001339185.1, YP_001381128.1, ZP_02007549.1, YP_001404661.1, YP_001405064.1, YP_001405512.1, YP_001405543.1, YP_001411516.1, NP_252992.1, NP_245786.1, NP_247766.1, YP_001431792.1, YP_001433313.1, NP_276814.1, YP_001435595.1, NP_344008.1, YP_001540259.1, YP_001541021.1, NP_353251.2, NP_376408.1, NP_384250.1, YP_001545273.1, YP_001547372.1, YP_001548956.1, ZP_02074485.1, NP_421736.1, ZP_02102447.1, ZP_02108920.1, YP_001579915.1, YP_001601954.1, NP_436098.1, ZP_02145782.1, ZP_02149604.1, ZP_02158968.1, ZP_02164719.1, ZP_02192950.1, ZP_02195223.1, YP_001634149.1, YP_001636009.1, YP_001639949.1, YP_001630687.1, ZP_02270251.1, ABZ09403.1, ZP_02297670.1, ZP_02359857.1, ZP_02371996.1, ZP_02374832.1, ZP_02377352.1, ZP_02379701.1, ZP_02381947.1, ZP_02388753.1, YP_001685812.1, ZP_02402916.1, ZP_02408490.1, ZP_02411445.1, ZP_02455737.1, ZP_02463569.1, ZP_02463674.1, ZP_02468918.1, ZP_02469138.1, ZP_02471320.1, ZP_02481786.1, ZP_02489989.1, ZP_02495341.1, ZP_02498121.1, ZP_02506137.1, ZP_02731746.1, ZP_02735156.1, ZP_02891835.1, ZP_02892690.1, YP_001773976.1, YP_001769273.1, YP_001756452.1, ZP_02906318.1, ZP_02909641.1, ACB59187.1, YP_001808149.1, NP_518773.1, NP_522646.1, YP_001824039.1, YP_001860507.1, YP_001861448.1, YP_786799.1, YP_001888358.1, YP_001890539.1, YP_001895289.1, YP_001925108.1, YP_001918469.1, YP_001796632.1, NP_578723.1, YP_001972626.1, YP_001976426.1, YP_001993174.1, YP_001999064.1, YP_002004737.1, YP_002009751.1, YP_002017711.1, YP_002028709.1, YP_002071066.1, YP_002091575.1, YP_002098526.1, YP_002131868.1, YP_002137871.1, YP_002135266.1, YP_002153700.1, YP_002182923.1, YP_002190241.1, YP_002196292.1, YP_002204862.1, NP_615207.1, NP_618844.1, NP_613991.1, YP_002256497.1, YP_002278394.1, YP_002283679.1, YP_002264711.1, YP_002290390.1, ZP_03286911.1, ZP_03296171.1, YP_002308062.1, NP_629161.1, NP_632962.1, NP_633540.1, YP_002311976.1, ZP_03452914.1, ZP_03455882.1, ZP_03473654.1, YP_002421473.1, YP_002437145.1, YP_002464278.1, YP_002464504.1, YP_002467674.1, YP_002488693.1, YP_002493410.1, YP_002497836.1, YP_002498084.1, YP_002499000.1, ZP_03546181.1, ZP_03550882.1, ZP_03570829.1, YP_002522227.1, YP_002524575.1, YP_002543001.1, YP_002553571.1, YP_002548136.1, ZP_03608360.1, YP_002565699.1, YP_002577029.1, YP_002577026.1, YP_002580457.1, YP_002581879.1, YP_002587403.1, XP_002336885.1, YP_002611872.1, ZP_03703827.1, YP_002679931.1, YP_002703544.1, YP_002707335.1, YP_002754213.1, ZP_03791879.1, ZP_03828535.1, ZP_03858274.1, ZP_03891452.1, ZP_03893668.1, ZP_03895581.1, YP_002822590.1, YP_002827947.1, YP_002828370.1, YP_002830641.1, YP_002831012.1, YP_002833375.1, ZP_03991277.1, ZP_03997736.1, ZP_04367788.1, ZP_04428509.1, ZP_04428624.1, YP_002836400.1, YP_002838947.1, YP_002841613.1, YP_002841804.1, YP_002844570.1, YP_002870312.1, ZP_04446760.1, ZP_04586840.1, YP_002896619.1, YP_002911791.1, NP_761182.1, NP_768076.1, NP_794587.1, NP_824429.1, AAP43988.1, NP_864249.1, NP_866979.1, NP_873736.1, NP_884614.1, NP_934800.1, NP_949019.1, NP_967131.1, NP_987160.1, YP_048904.1, YP_065266.1, YP_074219.1, YP_088969.1, AAU84351.1, YP_108414.1, YP_112198.1, YP_184266.1, YP_203899.1, YP_237461.1, YP_257837.1, YP_276552.1, AAF40195.1, YP_290330.1, YP_299600.1, YP_296852.1, YP_304289.1, YP_316917.1, CAE47792.1, YP_333439.1, YP_336473.1, YP_346379.1, YP_351954.1, YP_357165.1, ZP_00829731.1, YP_366883.1, YP_372811.1, YP_368902.1, YP_374576.1, YP_388855.1, YP_413037.1, YP_423107.1, YP_440462.1, YP_442977.1, YP_443057.1, ZP_00944947.1, ZP_00946618.1, ZP_00952881.1, ZP_00958802.1, ZP_00989819.1, YP_448186.1, ZP_01003995.1, ZP_01013057.1, ZP_01015154.1, ZP_01016944.1, YP_457372.1, ZP_01036255.1, ZP_01038966.1, ZP_01043023.1, ZP_01044498.1, YP_461336.1, ZP_01065773.1, YP_466032.1, YP_467759.1, YP_496797.1, YP_497130.1, ZP_01093096.1, ZP_01094388.1, YP_501803.1, YP_504571.1, YP_508936.1, ZP_01155438.1, ZP_01173081.1, ZP_01227676.1, YP_533570.1, YP_565011.1, YP_565185.1, YP_545749.1, YP_558219.1, YP_549369.1, YP_570647.1, YP_582796.1, YP_585797.1, ZP_01302260.1, YP_614343.1

**Large helicase related protein:** YP_629258.1, YP_634210.1, YP_638435.1, YP_672599.1, YP_680101.1, YP_658633.1, YP_689149.1, YP_715874.1, NP_069978.1, NP_071281.1, ZP_01463489.1, YP_766191.1, YP_827067.1, YP_842964.1, NP_148413.2, YP_883408.1, YP_923521.1, YP_920334.1, YP_931949.1, YP_946581.1, YP_952467.1, YP_863533.1, YP_984642.1, YP_001013134.1, YP_001020714.1, YP_001047419.1, YP_001069586.1, YP_001075778.1, YP_001044185.1, YP_001041345.1, ZP_01741823.1, ZP_01749331.1, YP_001096974.1, ZP_01769227.1, NP_111164.1, NP_111336.1, NP_126802.1, YP_001136066.1, YP_001158251.1, NP_143015.1, YP_001166785.1, YP_001171981.1, YP_001173155.1, YP_001194107.1, YP_001192176.1, YP_001187686.1, YP_685554.1, YP_685031.1, YP_001223393.1, YP_001266488.1, YP_001273075.1, YP_001277738.1, ZP_01883921.1, ZP_01914906.1, YP_001322982.1, YP_001329612.1, YP_001347232.1, YP_001372575.1, YP_001411733.1, NP_217813.1, NP_247267.1, YP_001431146.1, NP_276908.1, YP_001435756.1, YP_001462946.1, YP_001458435.1, NP_288089.1, NP_341677.1, YP_001536257.1, YP_001541686.1, NP_377362.1, YP_001549572.1, NP_394061.1, NP_394298.1, YP_001557834.1, YP_001564068.1, ZP_02083591.1, NP_416170.1, ZP_02112209.1, YP_001584388.1, YP_001602917.1, YP_001614610.1, ZP_02168383.1, ZP_02187452.1, ZP_02193814.1, YP_001623244.1, YP_001622601.1, ZP_02270333.1, YP_001670533.1, YP_001670575.1, ABZ08165.1, ZP_02292570.1, ZP_02381155.1, ZP_02407082.1, ZP_02451672.1, ZP_02459844.1, ZP_02475332.1, ZP_02485824.1, ZP_02493980.1, ZP_02510057.1, ZP_02551205.1, ZP_02731817.1, YP_001724949.1, YP_001737511.1, ZP_02884109.1, YP_001748006.1, YP_001709184.1, ZP_02931080.1, YP_001800912.1, NP_541440.1, YP_001820027.1, YP_001849551.1, YP_001862818.1, YP_001861407.1, YP_001888206.1, ZP_03002665.1, NP_578780.1, YP_001976789.1, ZP_03027777.1, ZP_03051201.1, YP_002008977.1, ZP_03058580.1, ZP_03068519.1, YP_002020538.1, YP_002124982.1, ZP_03131423.1, YP_002134144.1, ZP_03270572.1, ACI83028.1, ACI83029.1, YP_002293051.1, YP_002296889.1, YP_002306997.1, NP_629886.1, ZP_03417490.1, ZP_03421875.1, ZP_03426699.1, ZP_03450331.1, YP_002382543.1, YP_002387134.1, YP_002402883.1, YP_002412672.1, YP_002427847.1, ZP_03538432.1, YP_002458060.1, YP_002492273.1, YP_002494404.1, ZP_03569696.1, ZP_03588240.1, YP_002526376.1, YP_002551791.1, YP_002548442.1, ZP_03608458.1, YP_002565915.1, YP_002576263.1, YP_002576305.1, YP_002576390.1, YP_002577406.1, YP_002580151.1, YP_002583001.1, YP_002583000.1, YP_002628174.1, ZP_03693922.1, YP_002646367.1, ZP_03703619.1, ZP_03712290.1, YP_002713396.1, YP_002717888.1, ZP_03757467.1, ZP_03787326.1, ZP_03793628.1, YP_002762914.1, YP_002765546.1, ZP_03867169.1, ZP_03869612.1, ZP_03873648.1, ZP_03873964.1, ZP_03876454.1, ZP_03883291.1, ZP_03896728.1, ZP_03912261.1, ZP_03970488.1, ZP_03972450.1, YP_002830052.1, YP_002832810.1, ZP_03999705.1, ZP_04027499.1, ZP_04328027.1, ZP_04355115.1, ZP_04372038.1, ZP_04383260.1, ZP_04427273.1, YP_002839827.1, YP_002844003.1, YP_002870957.1, YP_002874980.1, ZP_04481226.1, NP_699996.1, ZP_04523219.1, NP_743264.1, NP_856969.1, NP_864488.1, NP_962349.1, NP_963296.1, NP_963693.1, NP_988261.1, YP_023658.1, YP_023972.1, CAG38618.1, YP_105668.1, YP_111300.1, YP_135636.1, YP_183177.1, YP_223196.1, ZP_00437990.1, YP_256114.1, YP_296062.1, YP_330730.1, YP_335463.1, YP_346937.1, YP_350760.1, YP_353733.1, YP_407928.1, YP_435296.1, ZP_00949403.1, YP_447211.1, ZP_00999835.1, ZP_01015146.1, ZP_01016308.1, ZP_01044966.1, ZP_01049559.1, ZP_01059502.1, YP_465299.1, YP_482609.1, YP_502071.1, ZP_01119721.1, ZP_01126102.1, ZP_01155446.1, YP_520007.1, YP_554153.1, YP_554526.1

**Phosphoenolpyruvate synthase:** YP_607393.1, AAA81512.1, YP_634917.1, YP_645793.1, , YP_657340.1, YP_657782.1, ABI15710.1, YP_726506.1, YP_733726.1, YP_742724.1, YP_750981.1, NP_069544.1, YP_844044.1, YP_869375.1, NP_147390.2, YP_888222.1, YP_902501.1, ZP_01618992.1, YP_919996.1, YP_931378.1, YP_963156.1, YP_969747.1, ZP_01666031.1, ZP_01676776.1, YP_987096.1, YP_974656.1, YP_001006416.1, YP_001020718.1, YP_001030975.1, ZP_01706437.1, YP_001047757.1, YP_001055214.1, YP_001040162.1, ZP_01731141.1, ZP_01739081.1, YP_001097019.1, YP_001104682.1, YP_001113801.1, NP_125790.1, YP_001136244.1, YP_001153655.1, NP_142107.1, YP_001183763.1, YP_001187578.1, YP_001176475.1, YP_686120.1, YP_001230065.1, YP_001273561.1, ZP_01894516.1, YP_001322939.1, YP_001324472.1, YP_001329566.1, YP_001348890.1, ZP_01984876.1, ZP_02008998.1, YP_001405322.1, NP_250461.1, NP_233371.1, NP_214468.1, NP_247521.1, NP_273662.1, NP_276246.1, YP_001435697.1, YP_001448635.1, YP_001453290.1, YP_001462997.1, YP_001478403.1, YP_001473610.1, NP_279426.1, YP_001502404.1, NP_288136.1, NP_310436.1, NP_342378.1, CAO91289.1, NP_377168.1, YP_001549617.1, YP_001566078.1, YP_001570653.1, YP_001598718.1, YP_001615213.1, ZP_02178637.1, ZP_02192452.1, ZP_02203813.1, YP_001655276.1, YP_001667834.1, NP_456162.1, YP_001673930.1, NP_460315.1, ZP_02658594.1, YP_001761086.1, ZP_02903337.1, YP_001794578.1, YP_001806106.1, NP_519542.1, NP_560002.1, YP_001863763.1, YP_001880462.1, YP_001898874.1, ZP_02960340.2, NP_577772.1, ZP_03029635.1, ZP_03046338.1, YP_002005682.1, ZP_03070659.1, YP_002126672.1, YP_002146688.1, YP_002215777.1, NP_618295.1, NP_613539.1, ZP_03219087.1, YP_002226730.1, YP_002253773.1, YP_002259616.1, YP_002243795.1, ACI82814.1, ZP_03282230.1, YP_002306693.1, NP_634747.1, YP_002311200.1, ZP_03316624.1, ZP_03320215.1, YP_002319847.1, ZP_03347441.1, ZP_03352005.1, ZP_03363594.1, YP_002382516.1, YP_002342264.1, YP_002428324.1, YP_002441145.1, YP_002467100.1, YP_002488139.1, YP_002509017.1, ZP_03550174.1, NP_669245.1, YP_002535616.1, ZP_03607800.1, YP_002565264.1, YP_002579557.1, YP_002583419.1, YP_002618074.1, ZP_03658687.1, ZP_03692897.1, ZP_03704379.1, ZP_03719481.1, YP_002668562.1, ZP_03745803.1, YP_002766844.1, ZP_03806626.1, ZP_03825383.1, ZP_03831043.1, ZP_03836532.1, ZP_03873078.1, ZP_03929339.1, ZP_03955498.1, YP_002829388.1, ZP_03996092.1, ZP_03997929.1, ZP_04339490.1, ZP_04403856.1, ZP_04415870.1, YP_002837519.1, YP_002840537.1, YP_002843314.1, ZP_04576092.1, ZP_04578282.1, ZP_04580858.1, ZP_04582938.1, YP_002849217.1, ZP_04599545.1, NP_718228.1, NP_799882.1, NP_842356.1, CAD56491.1, NP_988214.1, AAD28736.1, YP_024297.1, YP_049950.1, AAU84244.1, YP_136393.1, YP_150739.1, YP_171466.1, YP_181298.1, YP_183705.1, ZP_00517925.1, YP_256040.1, YP_264619.1, YP_296073.1, YP_326258.1, YP_347502.1, YP_357844.1, ZP_00823234.1, ZP_00826026.1, ZP_00830814.1, YP_385055.1, YP_399800.1, ZP_00946908.1, YP_445264.1, YP_447381.1, ZP_00996154.1, ZP_01043930.1, YP_474214.1, YP_477355.1, YP_504026.1, ZP_01131882.1, YP_540902.1, YP_574112.1

**Pre-mRNA splicing factor PRP8:** DAA01258.1, XP_001117328.1, XP_624014.2, XP_001218213.1, NP_001054734.1, NP_001056917.1, NP_619600.2, XP_782780.2, CAL55631.1, XP_001221630.1, XP_415805.2, XP_001015771.1, XP_001245132.1, XP_001265867.1, EAW90588.1, EAW90589.1, EAW90591.1, XP_001272909.1, XP_001330664.1, XP_001351366.1, XP_001360095.1, XP_001365062.1, YP_001110806.1, XP_001391816.1, XP_001420401.1, XP_001423560.1, XP_001434030.1, XP_359533.2, XP_001469202.1, BAF57625.1, XP_001486718.1, AAK73127.1, CAN66492.1, NP_001080253.1, XP_001525158.1, 2P87, 2P8R, XP_001515844.1, ABR67423.1, ABR67424.1, XP_001386263.2, EDN62404.1, NP_178124.1, AAK93250.1, YP_001359430.1, XP_001540722.1, XP_001554611.1, XP_001568496.1, XP_001595120.1, XP_001610701.1, XP_001613367.1, XP_001633987.1, XP_001647079.1, XP_001660510.1, CAO62710.1, CAO62711.1, XP_001665553.1, XP_001704838.1, XP_001689471.1, XP_001712807.1, XP_001713251.1, XP_001732213.1, XP_001733743.1, XP_001747121.1, XP_001768223.1, XP_001785044.1, XP_001791135.1, NP_957270.2, XP_001822074.1, XP_001835700.1, XP_001866804.1, XP_001875909.1, XP_001899767.1, XP_001908522.1, NP_498785.1, AAL56218.1, ABC00920.2, XP_001940650.1, EDK35997.2, ACE76957.1, ACE76969.1, ACE76971.1, ACE76975.1, ACE76978.1, EDV09210.1, NP_584759.1, NP_593861.1, XP_001948639.1, BAG59028.1, XP_001958961.1, XP_001975980.1, XP_001987870.1, XP_002004834.1, XP_002015433.1, XP_002033487.1, XP_002050969.1, XP_002074725.1, XP_002091123.1, XP_002076298.1, XP_002115184.1, CAG87634.2, NP_610735.1, EDZ71651.1, XP_002141530.1, XP_501687.2, CAP94634.1, EEB14455.1, XP_002150382.1, XP_002175593.1, EEC16083.1, EEC78578.1, EEC80085.1, EED16592.1, XP_002179646.1, XP_002215831.1, AAH34648.1, CAX15546.1, XP_002261014.1, XP_002163464.1, XP_002158199.1, EEE26885.1, EEE62437.1, EEE65158.1, EEF44818.1, CAX40364.1, XP_002287554.1, XP_002197327.1, XP_002327417.1, EEH11039.1, EEH18760.1, EEH36374.1, EEH48317.1, ACO70655.1, XP_002368068.1, EEP76930.1, CAY71142.1, XP_002379314.1, BAA22563.1, BAC30783.1, AAR00329.1, AAR00330.1, AAR00331.1, AAH64370.1, NP_195589.2, CAB38612.1, NP_983156.1, AAA67044.1, XP_382712.1, CAF90819.1, AAD29088.1, XP_447350.1, XP_451233.1, XP_459423.1, AAT79351.1, BAD67606.1, AAV91021.1, XP_566595.1, XP_308873.2, AAD55467.1, AAX38540.1, AAX38541.1, AAX38542.1, AAX38545.1, AAX38546.1, AAX38547.1, AAX38549.1, AAX38550.1, AAX38551.1, AAX39413.1, AAX39414.1, AAX39415.1, AAX39416.1, AAX39417.1, AAX39418.1, AAX39419.1, AAX39420.1, AAX39421.1, YP_224270.1, AAX89368.1, NP_012035.1, XP_626847.1, XP_644240.1, Q99PV0.1, XP_653245.1, XP_662127.1, XP_666793.1, XP_674592.1, XP_679499.1, XP_716279.1, XP_738254.1, XP_742575.1, XP_749438.1, XP_757683.1, XP_763310.1, XP_821624.1, XP_843501.1, XP_868438.1, XP_868443.1, XP_868445.1, XP_868448.1, XP_868451.1, XP_868455.1, XP_868460.1, XP_868464.1, XP_868467.1, XP_537770.2, XP_868472.1, XP_868477.1, XP_868480.1, XP_868483.1, BAE25786.1, AAX28469.2, XP_729464.1, ABC00915.1, ABC00916.1, ABC00917.1, ABC00918.1, ABC00919.1, YP_444182.1, XP_962953.1, XP_966931.1, NP_006436.3, ABF13297.1, CAJ14964.1, CAJ14965.1, CAJ14966.1, CAJ14967.1

**DNA-directed RNA polymerase subunit A:** YP_659142.1, NP_070550.1, YP_843669.1, YP_876741.1, YP_001031127.1, YP_001048029.1, YP_001098167.1, O57861.2, Q9HM33.1, NP_110554.1, NP_125813.1, NP_142130.1, YP_685573.1, ABQ75865.1, YP_001273957.1, YP_001323706.1, YP_001325330.1, YP_001330241.1, Q12TF2, YP_001403293.1, ABU41683.1, NP_248640.1, NP_276649.1, NP_280963.1, YP_001548970.1, NP_393515.1, YP_001583128.1, ZP_02193904.1, YP_001737956.1, NP_619411.1, NP_614933.1, P81409.2, Q97CR6.1, YP_002308029.1, NP_633270.1, YP_002467270.1, ZP_03608199.1, YP_002564825.1, YP_002576832.1, YP_002576918.1, YP_002580486.1, YP_002581808.1, ZP_03692289.1, ZP_03703985.1, ZP_03871459.1, ZP_03875039.1, ZP_03875310.1, ZP_03998642.1, BAA25164.1, NP_577748.2, NP_963703.1, NP_987146.1, YP_023336.1, CAG38138.1, YP_137169.1, YP_184316.1, AAZ32448.1, YP_304453.1, YP_325898.1, YP_447314.1, YP_503855.1, YP_567024.1

**Reverse gyrase/topoisomerase I:** YP_644723.1, ZP_01385599.1, YP_696389.1, YP_698989.1, ZP_01407769.1, YP_719942.1, YP_731316.1, YP_733719.1, YP_737712.1, YP_753530.1, NP_069857.1, NP_070633.1, ZP_01470755.1, YP_810595.1, YP_806621.1, YP_809252.1, YP_816589.1, YP_843026.1, AAA96962.1, NP_147880.2, NP_147906.2, YP_891380.1, YP_894518.1, ZP_01544322.1, YP_904210.1, ZP_01618852.1, YP_919983.1, YP_929913.1, YP_930753.1, YP_863029.1, NP_072784.1, YP_001001325.1, YP_974241.1, ZP_01688943.1, YP_001014318.1, YP_001012435.1, YP_001013109.1, YP_001013124.1, ZP_01706444.1, YP_001032579.1, YP_001055584.1, YP_001056006.1, YP_001040974.1, YP_001041125.1, YP_001041270.1, ZP_01725890.1, ZP_01727874.1, ZP_01729832.1, YP_001097306.1, NP_109949.1, NP_110538.1, YP_001125185.1, YP_001125646.1, YP_001125811.1, ZP_01772995.1, NP_126943.1, NP_127104.1, YP_001152524.1, ZP_01785353.1, ZP_01787162.1, ZP_01788623.1, ZP_01791743.1, ZP_01796959.1, NP_142581.1, NP_142736.1, NP_148170.1, YP_001183770.1, YP_001180363.1, YP_001191827.1, YP_001192298.1, YP_001211796.1, YP_686844.1, YP_001232415.1, YP_001244347.1, YP_001271380.1, YP_001273290.1, YP_001290656.1, YP_001291588.1, ZP_01818400.1, ZP_01820667.1, ZP_01824014.1, ZP_01830742.1, ZP_01834618.1, ZP_01860538.1, ZP_01870961.1, ZP_01872060.1, YP_001298646.1, YP_001304919.1, YP_001296841.1, YP_001322681.1, YP_001324231.1, YP_001329424.1, YP_001357114.1, YP_001357221.1, YP_001357405.1, ZP_01945693.1, ZP_01963616.1, ZP_01968323.1, YP_001398963.1, YP_001407082.1, YP_001404020.1, YP_001408974.1, YP_001410533.1, YP_001410867.1, ZP_02031525.1, YP_001421189.1, NP_213453.1, NP_213599.1, NP_213790.1, NP_227988.1, NP_248519.1, NP_248662.1, NP_267386.1, NP_273176.1, NP_276736.1, YP_001434598.1, YP_001435008.1, YP_001435105.1, YP_001467625.1, YP_001469829.1, YP_001483157.1, YP_001491118.1, YP_001530326.1, NP_350150.1, NP_341957.1, NP_342400.1, NP_342447.1, NP_345727.1, CAO87132.1, NP_358734.1, YP_001539953.1, YP_001540636.1, NP_376245.1, NP_377148.1, NP_377224.1, YP_001549743.1, NP_389494.1, NP_393542.1, ZP_02072545.1, ZP_02093224.1, YP_001577426.1, YP_001595977.1, YP_001620337.1, ZP_02177593.1, ZP_02178083.1, ZP_02178199.1, ZP_02179095.1, ZP_02179845.1, ZP_02180040.1, ZP_02193408.1, ZP_02208206.1, ZP_02210292.1, ZP_02212576.1, ZP_02218272.1, YP_001660677.1, YP_001665254.1, ZP_02330037.1, YP_001680778.1, ZP_02419079.1, ZP_02424432.1, ZP_02429020.1, ZP_02429843.1, ZP_02439327.1, ZP_02443688.1, ZP_02633952.1, ZP_02640977.1, ZP_02642614.1, ZP_02693499.1, ZP_02715323.1, ZP_02717324.1, ZP_02861742.1, ZP_02867363.1, YP_001708199.1, YP_001715103.1, YP_001696638.1, YP_001716766.1, AAB49283.1, YP_001735778.1, YP_001738810.1, YP_001736678.1, YP_001737502.1, YP_001793605.1, YP_001802651.1, YP_001814357.1, ZP_02953643.1, YP_001835757.1, NP_562618.1, NP_559079.1, NP_560414.1, YP_001843715.1, YP_001845107.1, YP_001917552.1, YP_001930557.1, YP_001931815.1, ZP_03008647.1, ZP_03014058.1, YP_001956618.1, YP_001960838.1, NP_578223.1, NP_578224.1, YP_001987564.1, ACF09891.1, YP_001999606.1, YP_001995351.1, YP_002003031.1, YP_002016861.1, YP_002019605.1, ZP_03073924.1, YP_002051028.1, YP_002105627.1, YP_002123348.1, ZP_03155722.1, YP_002135146.1, YP_002178593.1, ZP_03206896.1, ZP_03212922.1, NP_617416.1, NP_613336.1, NP_613576.1, NP_614887.1, 1GKU, 1GL9, ZP_03222470.1, YP_002251530.1, YP_002251235.1, NP_623067.1, NP_623335.1, ZP_03273798.1, YP_002297745.1, ZP_03288232.1, ZP_03296439.1, YP_002304395.1, YP_002306697.1, YP_002306713.1, NP_635100.1, YP_002316096.1, ZP_03300855.1, ZP_03303629.1, ZP_03309762.1, ZP_03330739.1, NP_660961.1, YP_002333963.1, YP_002335090.1, YP_002351969.1, YP_002353414.1, ZP_03471973.1, ZP_03478395.1, ZP_03489538.1, ZP_03497044.1, YP_002378700.1, YP_002341706.1, YP_002430354.1, YP_002428519.1, YP_002428729.1, YP_001741775.1, YP_002466356.1, YP_002508506.1, ZP_03556409.1, ZP_03568688.1, ACM09930.1, ACM09968.1, ACM09969.1, ACM09976.1, YP_002511104.1, YP_002522805.1, YP_002534056.1, YP_002560237.1, ZP_03608243.1, YP_002573005.1, YP_002574775.1, ZP_03611105.1, YP_002577503.1, YP_002577642.1, YP_002577710.1, YP_002577759.1, YP_002579738.1, YP_002580035.1, YP_002581633.1, YP_002582729.1, YP_002593732.1, ZP_03643592.1, YP_002606547.1, YP_002607471.1, YP_002614543.1, YP_002619828.1, YP_002628147.1, ZP_03656000.1, ZP_03661153.1, ZP_03662205.1, YP_002633979.1, ZP_03678135.1, ZP_03681865.1, ZP_03689077.1, ZP_03692755.1, ZP_03708440.1, YP_002653751.1, YP_002664856.1, YP_002670654.1, YP_002677509.1, ZP_03721282.1, YP_002709747.1, ZP_03734805.1, ZP_03741513.1, ZP_03745328.1, ZP_03748642.1, NP_688016.1, ZP_03749024.1, YP_002728159.1, YP_002728260.1, YP_002730192.1, YP_002731728.1, YP_002738435.1, YP_002740567.1, YP_002749246.1, YP_002744516.1, YP_002746515.1, YP_002772973.1, ZP_03822406.1, ZP_03869524.1, ZP_03895412.1, ZP_03900242.1, ZP_03908381.1, ZP_03916900.1, ZP_03930013.1, ZP_03945204.1, ZP_03946789.1, ZP_03970100.1, ZP_03973486.1, YP_002829314.1, YP_002829365.1, YP_002829732.1, YP_002832001.1, YP_002832053.1, YP_002832454.1, ZP_03998063.1, ZP_04010113.1, ZP_04039329.1, ZP_04043872.1, ZP_04339488.1, ZP_04355175.1, ZP_04356490.1, ZP_04358810.1, ZP_04378081.1, ZP_04421070.1, ZP_04433337.1, ZP_04433345.1, YP_002837496.1, YP_002837980.1, YP_002840153.1, YP_002840560.1, YP_002840612.1, YP_002843239.1, YP_002843291.1, YP_002843721.1, ZP_04446720.1, ZP_04450312.1, ZP_04498353.1, NP_692467.1, ZP_04539904.1, ZP_04555454.1, ZP_04565083.1, ZP_04567436.1, ZP_04581423.1, ZP_04583565.1, ZP_04584057.1, ZP_04584853.1, YP_002863425.1, ZP_04599685.1, YP_002915004.1, NP_735489.1, NP_757800.1, AAN87418.1, AAO73480.1, NP_820975.1, NP_844316.1, AAC26106.1, NP_895180.1, NP_907653.1, AAP58872.1, NP_953595.1, NP_963339.1, NP_963605.1, NP_963718.1, CAF28714.1, O67226.2, P74759.1, Q975P6.2, NP_988076.1, ZP_00157201.2, YP_005900.1, YP_023525.1, YP_036076.1, YP_045242.1, YP_060199.1, YP_067279.1, YP_075310.1, YP_083320.1, AAU82874.1, ZP_00154890.2, YP_130661.1, YP_145411.1, YP_143340.1, YP_147541.1, YP_175774.1, YP_179824.1, ZP_00370755.1, ZP_00372071.1, YP_182883.1, YP_183504.1, YP_193863.1, Q08582.3, YP_208895.1, CAI44249.1, CAI44289.1, CAI44333.1, CAI44357.1, CAI44381.1, ZP_00515556.1, YP_249232.1, YP_255499.1, YP_255998.1, YP_264942.1, YP_267940.1, XP_782119.1, YP_292959.1, YP_305910.1, YP_339866.1, ZP_00790908.1, YP_360614.1, YP_392687.1, YP_429226.1, ABC25363.1, ZP_00949093.1, YP_447412.1, ZP_01013502.1, YP_457851.1, ZP_01050174.1, ZP_01062257.1, ZP_01067561.1, ZP_01070534.1, ZP_01071150.1, YP_465906.1, YP_502480.1, ZP_01107501.1, ZP_01120499.1, ZP_01123080.1, ZP_01203320.1, ZP_01220202.1, ZP_01237025.1, YP_536196.1, CAJ72302.1, YP_564864.1, YP_565420.1, YP_544339.1, YP_563331.1, YP_581162.1

**UDP-glucose 6-dehydrogenase:** YP_624396.1, YP_629309.1, NP_052791.1, YP_677522.1, YP_741134.1, YP_747313.1, ZP_01459452.1, YP_780052.1, YP_847478.1, YP_855539.1, YP_864265.1, YP_897387.1, YP_897463.1, ZP_01546805.1, YP_911980.1, YP_928111.1, YP_932580.1, YP_933736.1, YP_949777.1, YP_001003744.1, ZP_01693795.1, YP_001019803.1, EAY55816.1, YP_001037777.1, ZP_01721057.1, YP_001089284.1, ZP_01741978.1, YP_001097024.1, YP_001115663.1, YP_001114355.1, NP_105958.1, YP_001124847.1, CAM74702.1, YP_001131571.1, YP_001166790.1, YP_001170255.1, YP_001202917.1, YP_001243123.1, BAF62918.1, ZP_01856440.1, ZP_01865350.1, ZP_01889238.1, ZP_01901626.1, ZP_01904528.1, ZP_01916661.1, YP_001300408.1, YP_001305128.1, YP_001311802.1, YP_001322933.1, YP_001324612.1, YP_001330564.1, YP_001380392.1, YP_001381609.1, ZP_01959029.1, YP_001395482.1, ZP_02031959.1, YP_001422834.1, YP_001422905.1, YP_001424294.1, NP_213002.1, NP_248048.1, YP_001478937.1, YP_001493952.1, YP_001496849.1, YP_001499752.1, YP_001516690.1, YP_001548652.1, NP_391438.1, NP_391504.1, ZP_02064767.1, ZP_02065580.1, ZP_02069246.1, ZP_02072902.1, ZP_02081577.1, NP_421182.1, NP_421185.1, YP_001580435.1, YP_080885.2, ZP_02147441.1, ZP_02150900.1, ZP_02161822.1, ZP_02167492.1, ZP_02171958.1, ZP_02191825.1, YP_001636440.1, YP_001639418.1, YP_001640488.1, YP_001641128.1, YP_001647840.1, ZP_02242720.1, YP_001663879.1, ZP_02376243.1, ZP_02467906.1, ZP_02642069.1, ZP_02845456.1, ZP_02845499.1, YP_001735573.1, ZP_02883623.1, ZP_02914245.1, ZP_02927845.1, NP_484702.1, ZP_02951626.1, NP_561410.1, YP_001838337.1, YP_001855119.1, YP_001856985.1, YP_001857293.1, YP_001868435.1, YP_001878038.1, YP_001895588.1, YP_001898366.1, YP_001924537.1, YP_001925903.1, YP_001918684.1, YP_001904174.1, YP_001938669.1, ZP_03014480.1, ZP_03014907.1, YP_001972915.1, YP_001983887.1, ZP_03053936.1, YP_002099562.1, YP_002121165.1, ZP_03100198.1, ZP_03112817.1, ZP_03112941.1, ZP_03128875.1, ZP_03152563.1, YP_002131799.1, YP_002133478.1, YP_002136773.1, YP_002233475.1, EDZ39293.1, YP_002248830.1, YP_002250310.1, ZP_03231229.1, ZP_03265857.1, ZP_03268147.1, YP_002297909.1, ZP_03293023.1, YP_002303634.1, NP_636875.1, NP_641884.1, ZP_03301469.1, ZP_03306597.1, EEC02126.1, NP_662068.1, YP_002354776.1, YP_002355506.1, YP_002420999.1, YP_002422017.1, YP_001741525.1, YP_002462456.1, CAJ97420.1, YP_002483976.1, YP_002491596.1, YP_002494843.1, YP_002510017.1, ZP_03557921.1, ZP_03574305.1, ZP_03586534.1, ZP_03593356.1, YP_002522041.1, YP_002595908.1, ZP_03631393.1, YP_002591771.1, YP_002629277.1, ZP_03679136.1, ZP_03680448.1, ZP_03699496.1, YP_002659763.1, YP_002690172.1, YP_002700430.1, YP_002707075.1, ZP_03732305.1, ZP_03765455.1, YP_002722700.1, YP_002731432.1, YP_002752541.1, YP_002752623.1, YP_002772004.1, YP_002799926.1, ZP_03854314.1, ZP_03888253.1, ZP_03897940.1, ZP_03969010.1, YP_002825381.1, ZP_04047009.1, YP_002851468.1, ZP_04087282.1, ZP_04093210.1, ZP_04093279.1, ZP_04111191.1, ZP_04117515.1, ZP_04123816.1, ZP_04171866.1, ZP_04177169.1, ZP_04188773.1, ZP_04188846.1, ZP_04201000.1, ZP_04209442.1, ZP_04225370.1, ZP_04225444.1, ZP_04253886.1, ZP_04265422.1, ZP_04276146.1, ZP_04292140.1, ZP_04303368.1, ZP_04309558.1, ZP_04314553.1, ZP_04320461.1, ZP_04328577.1, ZP_04343663.1, ZP_04353595.1, ZP_04353600.1, ZP_04358397.1, ZP_04359258.1, ZP_04424361.1, ZP_04491001.1, ZP_04494605.1, ZP_04503031.1, ZP_00052125.1, NP_693806.1, NP_693851.1, ZP_04531439.1, ZP_04540562.1, ZP_04543727.1, ZP_04545152.1, ZP_04554114.1, ZP_04568292.1, ZP_04570551.1, YP_002905598.1, NP_769023.1, NP_774769.1, NP_809742.1, NP_819866.1, NP_882231.1, NP_886356.1, NP_891348.1, NP_902711.1, ZP_00144068.1, NP_923915.1, NP_924008.1, NP_924744.1, NP_929736.1, NP_962759.1, NP_981600.1, NP_981677.1, YP_039188.1, YP_061472.1, YP_086464.1, YP_093313.1, YP_099578.1, YP_114899.1, YP_177180.1, YP_200705.1, YP_211199.1, YP_212010.1, YP_247261.1, YP_284506.1, YP_314719.1, ZP_00740533.1, YP_325081.1, YP_348548.1, YP_353728.1, YP_363325.1, YP_419939.1, YP_436052.1, ZP_00946828.1, YP_446801.1, ZP_00948882.1, ZP_00954337.1, ZP_00959576.1, YP_447270.1, ZP_01002138.1, ZP_01003638.1, YP_450975.1, ZP_01013266.1, ZP_01070122.1, YP_464269.1, YP_467505.1, ZP_01104648.1, ZP_01120032.1, ZP_01126982.1, YP_508018.1, ZP_01157735.1, YP_537245.1, ZP_01252523.1, YP_558776.1, ZP_01288503.1, ZP_01302872.1, ZP_01304339.1, YP_611170.1, EEF24891.1, NP_173979.1, ACG33823.1, AAO62313.1, ABG22007.1, EEB18669.1, EDS38675.1, EAT36695.1, XP_971267.1, AAB63462.1

**Dna polymerase A:** CAA61369.1, AAA79862.1, BAE95222.1, CAC12847.1, CAC12848.1, XP_001116065.1, XP_001116048.1, , YP_657473.1, YP_659142.1, NP_067694.1, AAG30070.1, YP_717843.1, NP_001039204.1, NP_069333.1, NP_070550.1, XP_001211449.1, NP_001067405.1, CAL52459.1, XP_001225371.1, YP_802988.1, YP_843669.1, YP_843812.1, ABK59374.1, XP_001032353.1, NP_148383.2, YP_876741.1, XP_001239851.1, CAC19127.1, AAG41999.1, XP_001261127.1, YP_919805.1, YP_930480.1, NP_073324.1, XP_001275938.1, XP_001326973.1, Q3KSP1.1, YP_001013090.1, YP_001030057.1, YP_001031127.1, XP_001347450.1, YP_001033959.1, XP_001353021.1, YP_001046713.1, YP_001048029.1, YP_001055940.1, YP_001040896.1, CAM46996.1, Q9LVN7.1, P46588.2, NP_077578.1, NP_078724.1, YP_001097770.1, YP_001098167.1, XP_772922.1, O57861.2, Q9HM33.1, NP_110554.1, NP_111342.1, AAK30251.1, Q9HH84.1, YP_001129507.1, YP_001129355.1, AAH08800.1, AAH09128.1, BAB59979.1, NP_125813.1, NP_126386.1, NP_127396.1, XP_001397242.1, XP_001416112.1, YP_001154087.1, NP_142130.1, NP_143776.1, XP_001468284.1, YP_001192217.1, CAG38139.2, XP_001486690.1, NP_001087694.1, YP_687422.1, YP_685573.1, ABQ75865.1, YP_001273614.1, YP_001273957.1, YP_001274054.1, EDL22729.1, EDL22730.1, EDL22732.1, EDL22733.1, EDL22734.1, EDM07474.1, EDM07475.1, EDM07476.1, EDM07477.1, XP_001523599.1, CAA90888.1, CAA90887.1, XP_001493912.1, YP_001323706.1, YP_001323876.1, YP_001324849.1, YP_001325330.1, YP_001330241.1, YP_001330592.1, XP_001386065.2, ABH05845.2, ABH05847.2, EDN60257.1, Q12TF2.2, ABO40783.1, ABO40787.1, AAW55994.2, ABH08756.2, YP_001403293.1, YP_001404269.1, XP_001538893.1, XP_001553035.1, XP_001567964.1, ABT13573.1, YP_001425655.1, YP_001427279.1, ABU41683.1, XP_001587512.1, XP_001610797.1, ABU52897.1, ABU52899.1, ABU52901.1, AAQ10669.2, XP_001641357.1, XP_001602680.1, XP_001606357.1, NP_002682.2, NP_247880.1, NP_248640.1, NP_275351.1, NP_276336.1, NP_276649.1, XP_001645504.1, YP_001434653.1, ABV03812.1, XP_001648163.1, CAO68524.1, XP_001673634.1, XP_001685930.1, NP_279569.1, NP_280963.1, YP_001497445.1, YP_001498312.1, ABN49965.2, NP_341651.1, YP_001541158.1, NP_378066.1, XP_001689909.1, YP_001548579.1, YP_001548970.1, NP_394366.1, NP_393515.1, YP_001582282.1, YP_001583128.1, YP_001595175.1, YP_001616140.1, ZP_02193714.1, ZP_02193904.1, AAI57513.1, YP_001648316.1, XP_001728836.1, XP_001735501.1, XP_001748370.1, XP_001754656.1, XP_001762435.1, YP_001687027.1, XP_001801013.1, ACA65605.1, ACA65651.1, ACA65700.1, ACA65716.1, ACA65733.1, XP_001822406.1, XP_001827844.1, XP_001831621.1, XP_001868453.1, XP_001883646.1, YP_001737626.1, YP_001737956.1, XP_001892851.1, P52431.1, P52342.1, P52025.1, YP_001794693.1, XP_001908464.1, XP_001911857.1, AAC57086.1, AAN35123.2, ABI21851.2, AAA35303.1, Q9HH05.1, NP_506017.1, NP_542554.1, AAA58439.1, AAA35768.1, NP_559770.1, AAB47255.1, 2JGU, NP_201201.2, NP_570750.1, XP_001940287.1, XP_967290.2, NP_577941.1, AAI63875.1, EDK35969.2, A7U6F1.1, NP_596124.1, ACF09484.1, XP_001948892.1, 2VWJ, 2VWK, XP_001957325.1, XP_001973157.1, XP_001984922.1, XP_002008314.1, XP_002026913.1, XP_002030664.1, XP_002030665.1, XP_002047697.1, XP_002069178.1, XP_002086418.1, XP_002085037.1, XP_002108586.1, 3CQ8, XP_002125320.1, CAR66020.1, NP_615844.1, NP_619411.1, NP_614322.1, NP_614933.1, P81409.2, Q97CR6.1, YP_002249153.1, EDZ73334.1, XP_002142640.1, YP_002306381.1, YP_002308029.1, NP_633270.1, NP_634028.1, XP_002145577.1, XP_002174009.1, ACJ70679.1, ACJ70688.1, ACJ70693.1, EEC67820.1, YP_002428988.1, XP_002181135.1, XP_002225541.1, XP_002225545.1, YP_002466770.1, YP_002467270.1, EEE25368.1, ZP_03607853.1, ZP_03608199.1, YP_002564825.1, YP_002564852.1, EEE51779.1, ACN10946.1, XP_002291074.1, XP_002307042.1, AAB62593.1, ZP_03692289.1, ZP_03692726.1, 3CFO, ZP_03703985.1, XP_002264385.1, EEH04243.1, EEH20363.1, EEH35554.1, EEH44767.1, EEH58614.1, YP_002830079.1, YP_002832837.1, ZP_03997886.1, ZP_03998642.1, CAD42683.1, YP_002854004.1, YP_002854382.1, YP_002838337.1, YP_002839800.1, YP_002844031.1, P30320.1, P30317.1, ACQ99189.1, XP_002365027.1, ACR33068.1, YP_002922395.1, EEQ31043.1, EEQ39055.1, EEQ47058.1, EEQ76253.1, EEQ82248.1, EEQ84437.1, EER05102.1, EER05103.1, YP_002958492.1, YP_002959821.1, EER25236.1, EER42060.1, ZP_04789949.1, ZP_04790791.1, XP_002421460.1, XP_002399458.1, XP_002429857.1, XP_002451581.1, XP_002450450.1, YP_002994326.1, YP_002994988.1, XP_002486944.1, NP_524099.2, Q51334.1, NP_733857.1, ZP_04874022.1, ZP_04874443.1, ZP_04875597.1, ZP_04875621.1, ZP_04876556.1, ZP_04876963.1, ZP_04879299.1, ZP_04880093.1, XP_002491049.1, XP_002494695.1, NP_035261.3, ZP_05303418.1, ZP_05304367.1, XP_002502470.1, EET90265.1, EET90381.1, XP_002515746.1, XP_002554637.1, XP_002550800.1, YP_003097306.1, XP_002568889.1, XP_002579779.1, EEU09012.1, EEU44688.1, YP_003128628.1, YP_003128772.1, YP_003129295.1, YP_003130526.1, ZP_05570619.1, ZP_05571288.1, YP_003176601.1, YP_003178706.1, XP_002584155.1, EEW27563.1, EEW29508.1, 3IAY, CAY78407.1, BAC40275.1, NP_776852.1, AAB99910.1, BAA25164.1, P97283.1, NP_861746.1, NP_577748.2, CAA33504.1, AAA72101.1, O05706.1, O33845.1, O48901.1, P74918.1, Q56366.1, XP_362488.1, BAA35142.1, AAS01671.1, NP_963362.1, NP_963703.1, NP_963808.1, CAA43922.1, AAA67132.1, AAA67131.1, BAA75663.1, NP_985361.1, NP_987146.1, NP_987500.1, XP_387351.1, CAG02717.1, YP_022906.1, YP_023336.1, CAG38138.1, CAA41968.1, XP_449097.1, XP_454020.1, XP_462512.1, XP_499875.1, YP_068007.1, YP_073706.1, 1WNS, YP_136425.1, YP_137169.1, YP_142676.1, YP_182414.1, YP_184316.1, YP_182359.1, XP_567620.1, P56689.1, BAD92797.1, AAX97697.1, AAX97698.1, NP_010181.1, XP_627772.1, XP_623795.1, XP_638283.1, BAA06142.2, XP_654477.1, XP_673793.1, XP_717004.1, AAY98790.1, BAE06251.1, YP_254794.1, XP_741713.1, XP_755997.1, XP_757605.1, YP_268993.1, XP_805939.1, XP_808567.1, AAZ32448.1, AAZ32459.1, CAB81809.1, 1WN7, YP_304453.1, YP_305298.1, YP_293784.1, XP_851285.1, XP_863454.1, BAA93703.1, Q7SIG7.1, Q9HH06.1, 1D5A, AAF66765.1, YP_325898.1, YP_326412.1, XP_888889.1, ABB29977.1, BAA07580.1, BAE48221.1, YP_401712.1, XP_729844.1, YP_446104.1, XP_951513.1, YP_447314.1, YP_448301.1, YP_448519.1, CAJ57159.1, XP_955596.1, XP_961558.1, 1QHT, ABD14868.1, ABD14869.1, YP_502623.1, YP_503855.1, ZP_01167632.1, AAF81662.1, NP_001034899.1, YP_566332.1, YP_567024.1, CAA61282.1, NP_044849.1, NP_048532.1, NP_049662.1, 2CW7, 2CW8

**DNA polymerase III:** ZP_01367072.1, YP_633981.1, YP_643874.1, YP_714599.1, YP_721618.1, YP_721633.1, YP_730835.1, YP_742683.1, YP_754708.1, ZP_01451950.1, ZP_01467326.1, ZP_01467711.1, ZP_01471881.1, YP_821313.1, YP_845352.1, YP_855729.1, YP_867125.1, YP_872813.1, YP_901734.1, ZP_01547419.1, YP_909993.1, ZP_01621251.1, ZP_01631782.1, ZP_01632016.1, YP_967159.1, ZP_01666082.1, YP_974500.1, YP_200601.6, YP_001011342.1, YP_001009306.1, ZP_01687668.1, YP_001017592.1, YP_001014757.1, EAY56352.1, YP_001037689.1, ZP_01721208.1, ZP_01730819.1, ZP_01731243.1, ZP_01738550.1, YP_001091137.1, YP_001089916.1, YP_001113654.1, Q9XDH5.1, YP_001142887.1, YP_001158639.1, ZP_01784180.1, YP_001180603.1, YP_001212770.1, YP_001224790.1, YP_001227581.1, YP_001222592.1, YP_001255856.1, YP_001274482.1, ZP_01856577.1, ZP_01859634.1, ZP_01875209.1, ZP_01895205.1, ZP_01908355.1, YP_001321812.1, YP_001362915.1, YP_001345148.1, YP_001380717.1, ZP_01947159.1, ZP_01965690.1, YP_001392730.1, AAA27191.1, ZP_02029785.1, ZP_02037903.1, YP_001422217.1, YP_001424773.1, NP_252330.1, NP_213688.1, ABV27346.1, YP_001484146.1, YP_001487786.1, NP_294230.1, YP_001506714.1, YP_001512141.1, YP_001515102.1, YP_001516834.1, NP_297205.1, CAO86641.1, CAO86680.1, YP_001536662.1, YP_001550584.1, ZP_02062302.1, ZP_02079117.1, ZP_02087260.1, YP_001597182.1, NP_440562.1, NP_443058.1, ZP_02177293.1, ZP_02189611.1, ZP_02206739.1, YP_001625621.1, YP_001631728.1, CAP09635.1, ZP_02210717.1, ZP_02234948.1, YP_001654878.1, YP_001658767.1, YP_001658930.1, ZP_02243954.1, YP_001664680.1, YP_001662822.1, ZP_02330165.1, YP_001678720.1, ZP_02432860.1, ZP_02443399.1, ZP_02613608.1, ZP_02616639.1, ZP_02732675.1, ZP_02848221.1, YP_001717194.1, YP_001734655.1, YP_001735617.1, YP_001783011.1, YP_001788703.1, YP_001710109.1, ZP_02918281.1, ZP_02928956.1, YP_001805491.1, YP_001806137.1, YP_001800580.1, NP_485097.1, NP_487618.1, P74750.2, YP_001840281.1, YP_001855324.1, YP_001868161.1, YP_001868929.1, YP_786753.1, ZP_02993162.1, ZP_02959074.2, YP_001916733.1, YP_001904343.1, YP_001939561.1, YP_001952956.1, YP_001954089.1, YP_001955969.1, YP_001971333.1, ZP_03026121.1, YP_001981623.1, ZP_03039171.1, YP_001998480.1, ZP_03055912.1, BAG55471.1, YP_002027639.1, YP_002048893.1, YP_002082567.1, YP_002122271.1, ZP_03128383.1, ZP_03142087.1, ZP_03145429.1, ZP_03157370.1, NP_604280.1, YP_002139890.1, YP_002135867.1, YP_002185730.1, YP_002222225.1, YP_002223034.1, 3E0D, EDZ38838.1, YP_002247456.1, YP_002251105.1, NP_623406.1, ZP_03271495.1, YP_002297437.1, ZP_03292734.1, ZP_03298485.1, YP_002303219.1, NP_636732.1, NP_639831.1, NP_641741.1, ZP_03312610.1, ZP_03314390.1, ZP_03323656.1, YP_002322298.1, ZP_03446321.1, YP_002353279.1, ZP_03464985.1, YP_002373829.1, ZP_03473075.1, ZP_03497707.1, YP_002378667.1, YP_002380125.1, YP_002430102.1, YP_002434559.1, YP_001741788.1, YP_002458874.1, YP_002479814.1, YP_002485005.1, YP_002493989.1, YP_002508070.1, ZP_03567758.1, YP_002573135.1, NP_682846.1, NP_682859.1, YP_002597090.1, ZP_03640833.1, ZP_03645919.1, ZP_03649461.1, YP_002603739.1, YP_002613461.1, YP_002616796.1, YP_002619607.1, ZP_03687577.1, ZP_03708072.1, ZP_03717214.1, YP_002673705.1, YP_002654793.1, YP_002662651.1, YP_002670263.1, YP_002708461.1, YP_002711683.1, YP_002716531.1, ZP_03734301.1, ZP_03742477.1, ZP_03759548.1, ZP_03764916.1, ZP_03767201.1, ZP_03774236.1, ZP_03780409.1, YP_002720784.1, YP_002728038.1, YP_002730690.1, YP_002756478.1, YP_002760249.1, YP_002766978.1, YP_002770858.1, YP_002786979.1, ZP_03816088.1, ZP_03857755.1, ZP_03866077.1, ZP_03867121.1, ZP_03882485.1, ZP_03894187.1, ZP_03903329.1, ZP_03907876.1, ZP_03914981.1, ZP_03924409.1, ZP_03924974.1, ZP_03927825.1, ZP_03930193.1, ZP_03937665.1, ZP_03975635.1, ZP_03978984.1, ZP_03989532.1, ZP_03991564.1, ZP_03993410.1, ZP_04028311.1, ZP_04032734.1, ZP_04035301.1, ZP_04041833.1, YP_002850740.1, ZP_04344281.1, ZP_04349674.1, ZP_04353316.1, ZP_04356763.1, ZP_04366293.1, ZP_04369832.1, ZP_04384264.1, ZP_04424301.1, ZP_04424303.1, ZP_04445269.1, ZP_04447637.1, YP_002882411.1, ZP_04452125.1, ZP_04455717.1, ZP_04468722.1, ZP_04494716.1, ZP_04498142.1, ZP_04508728.1, ZP_00121872.1, NP_695356.1, ZP_04532669.1, ZP_04570411.1, ZP_04571392.1, ZP_04575862.1, YP_002893281.1, 2KEQ, NP_710439.1, AAO72750.1, NP_820326.1, NP_828947.1, NP_865317.1, NP_875143.1, NP_880970.1, NP_883982.1, NP_893063.1, NP_894480.1, NP_897125.1, ZP_00144477.1, AAQ86808.1, AAQ86809.1, NP_926880.1, AAP47636.1, AAP47637.1, AAP47638.1, AAP47639.1, AAP47640.1, AAP47641.1, NP_952453.1, NP_968930.1, NP_973384.1, YP_005775.1, YP_010572.1, YP_055857.1, YP_062427.1, YP_065399.1, YP_080215.1, YP_113748.1, YP_143446.1, YP_171662.1, YP_172608.1, YP_182167.1, ZP_00371595.1, AAW75216.1, YP_219504.1, AAY41168.1, ZP_00514077.1, ZP_00515421.1, YP_288108.1, YP_291460.1, YP_322121.1, YP_324248.1, YP_356641.1, YP_359983.1, YP_363194.1, YP_377309.1, YP_375167.1, YP_381468.1, YP_384177.1, YP_388688.1, YP_397350.1, YP_401216.1, CAI78453.1, YP_413410.1, YP_430715.1, YP_433123.1, YP_445111.1, ZP_00994848.1, YP_450881.1, YP_455608.1, YP_461659.1, YP_466649.1, YP_473551.1, YP_477421.1, YP_486215.1, ZP_01080084.1, ZP_01085043.1, ZP_01089033.1, 1ZD7, 1ZDE, ZP_01104443.1, ZP_01113858.1, ZP_01123941.1, ZP_01173231.1, YP_517545.1, YP_515848.1, YP_526580.1, ZP_01223742.1, CAJ72534.1, YP_569987.1, ZP_01287194.1, ZP_01290812.1, YP_588968.1, YP_591822.1, YP_603727.1, YP_594634.1, ZP_01313157.1

**DNA primase/helicase:** NP_706610.1, YP_690085.1, YP_001648921.1, ZP_04535347.1, YP_002328245.1, ZP_03027318.1, YP_215328.1, YP_002533512.1, YP_224199.1, YP_002636888.1, YP_0021.1, ZP_02928292.1, YP_001155208.1, YP_001765176.1, YP_002094030.1, YP_549845.1, CAJ57176.1, YP_001610249.1, YP_001610249.1, YP_988524.1, YP_988524.1, YP_032692.1, YP_032692.1, YP_034154.1, YP_034154.1, NP_823847.1, AAN62835.1, CAJ57158.1, YP_418072.1, YP_002181974.1, ACO69124.1, ZP_03497306.1, NP_294982.1, NP_817458.1, EEH53892.1, YP_002014477.1, NP_813761.1, XP_001418285.1, YP_002308398.1, YP_002003469.1, YP_918997.1, XP_001772803.1, YP_918996.1, BAD46002.1, EEC81157.1, ZP_04503256.1, YP_656186.1, YP_002224207.1, XP_002299018.1, YP_002048644.1, YP_249580.2, NP_744419.1, AAY46279.1, NP_947593.1, YP_418073.1, CAL53634.1, NP_706610.1, YP_690085.1, YP_001648921.1, ZP_04535347.1, YP_002328245.1, ZP_03027318.1, YP_215328.1, YP_002533512.1, YP_224199.1, YP_002636888.1, YP_002113588.1, YP_124429.1, YP_002440123.1, XP_002335987.1, XP_002337405.1, ZP_02006408.1, ZP_02928292.1, YP_001155208.1, YP_001765176.1, YP_002094030.1, YP_549845.1, CAJ57176.1, YP_001610249.1, YP_001610249.1, YP_988524.1, YP_988524.1, YP_032692.1, YP_032692.1, YP_034154.1, YP_034154.1, NP_823847.1, AAN62835.1, CAJ57158.1, YP_418072.1, YP_002181974.1, ACO69124.1, ZP_03497306.1, NP_294982.1, NP_817458.1, EEH53892.1, YP_002014477.1, NP_813761.1, XP_001418285.1, YP_002308398.1, YP_002003469.1, YP_918997.1, XP_001772803.1, YP_918996.1, BAD46002.1, EEC81157.1, ZP_04503256.1, YP_656186.1, YP_002224207.1, XP_002299018.1, YP_002048644.1, YP_249580.2, NP_744419.1, AAY46279.1, NP_947593.1, YP_418073.1, CAL53634.1, YP_690085.1, YP_001648921.1, NP_706610.1, YP_002328245.1, YP_002328245.1, ZP_04535347.1, ZP_04535347.1, ZP_03027318.1, ZP_03027318.1, YP_215328.1, YP_215328.1, YP_002533512.1, YP_002533512.1, YP_224199.1, YP_224199.1, YP_002636888.1, YP_002636888.1, YP_002113588.1, YP_002113588.1, YP_124429.1, YP_124429.1, YP_002440123.1, YP_002440123.1, XP_002335987.1, XP_002335987.1, XP_002337405.1, ZP_02006408.1, ZP_02006408.1, YP_001155208.1, YP_001648922.1, ZP_02928292.1, ZP_02928292.1, YP_001765176.1, YP_002094030.1, YP_549845.1, CAJ57176.1, ZP_02499095.1, ZP_03452711.1, YP_002097751.1, ZP_02890160.1, YP_001101264.1, YP_001610249.1, YP_001610249.1, YP_001610249.1, AAN62835.1, NP_888753.1, YP_988524.1, YP_988524.1, YP_988524.1, YP_032692.1, YP_032692.1, YP_032692.1, NP_823847.1, YP_034154.1, YP_034154.1, YP_034154.1, YP_418072.1, CAJ57158.1, YP_002181974.1, YP_001562958.1, ZP_03497306.1, YP_441469.1, YP_642522.1, YP_879378.1, NP_817458.1, YP_002014477.1, YP_002224207.1, YP_656186.1, ZP_04365119.1, Q9F5P4.1, ACO69124.1, NP_294982.1, EEH53892.1, NP_813761.1, XP_001418285.1, NP_294983.1, YP_002308398.1, XP_001772803.1, YP_002003469.1, BAD46002.1, YP_918997.1, EEC81157.1, YP_918996.1, ZP_04503256.1, XP_002299018.1, YP_224270.1, YP_002048644.1, YP_249580.2, NP_744419.1, AAY46279.1, YP_418073.1, ZP_04334718.1, NP_959005.1, YP_002322708.1, NP_947593.1

**DNAB helicase:** YP_001324295.1, NP_248116.1, NP_988010.1, NP_988010.1, YP_001549808.1, YP_001549808.1, YP_001329359.1, YP_001329359.1, YP_001097223.1, YP_001097223.1, YP_001322557.1, YP_001322557.1, YP_001097458.1, YP_001097458.1, ZP_02192479.1, ZP_02192479.1, YP_001097792.1, YP_001097792.1, ABD17736.1, ABD17736.1, YP_001548609.1, YP_001548609.1, YP_183745.1, YP_183745.1, YP_685308.1, YP_685308.1, NP_143168.1, NP_143168.1, NP_126564.1, NP_126564.1, NP_578406.1, NP_578406.1, YP_001405615.1, YP_001405615.1, YP_002583268.1, YP_002583268.1, YP_503885.1, YP_503885.1, ZP_03998706.1, ZP_03998706.1, NP_280985.1, NP_280985.1, NP_632449.1, NP_632449.1, YP_565780.1, YP_565780.1, YP_137330.1, YP_137330.1, NP_618094.1, NP_618094.1, YP_002307730.1, YP_002307730.1, YP_325906.1, YP_325906.1, YP_306959.1, YP_306959.1, YP_656795.1, YP_656795.1, YP_002580831.1, YP_002580831.1, YP_002567343.1, YP_002567343.1, ZP_03875384.1, ZP_03875384.1, YP_843229.1, YP_843229.1, YP_001048404.1, YP_001048404.1, YP_001540156.1, YP_001540156.1, ZP_03692258.1, ZP_03692258.1, 2P6R, 2P6R, NP_071282.1, NP_071282.1, YP_001031179.1, YP_001031179.1, YP_002467772.1, YP_002467772.1, YP_002577078.1, YP_002577078.1, YP_002579102.1, YP_002579102.1, ZP_03997680.1, YP_919908.1, NP_558924.1, YP_657401.1, YP_930665.1, NP_376477.1, NP_376477.1, YP_002565871.1, YP_002913571.1, YP_002913571.1, NP_343811.1, YP_002831080.1, YP_002828439.1, YP_002828439.1, Q97VY9.1, YP_002841682.1, 2VA8, 2VA8, YP_001054984.1, YP_254972.1, NP_613398.1, YP_001152379.1, YP_001793507.1, ABZ07376.1, ABZ08606.1, YP_001191456.1, YP_876638.1, YP_001435870.1, ZP_03871982.1, NP_147034.2, NP_147034.2, YP_024158.1, YP_001040230.1, XP_969311.1, XP_002046924.1, XP_002084061.1, XP_615375.3, XP_001502374.2, NP_577782.1, XP_001850567.1, XP_002120889.1, NP_248404.1, XP_001419844.1, NP_142063.1, YP_002307364.1, NP_001078482.1, YP_001522732.1, XP_001120279.1, XP_002164745.1, NP_614118.1, XP_420565.1, YP_001329748.1, CAP97503.1, YP_001096840.1, NP_988404.1, XP_847166.1, XP_001212516.1, ZP_03524453.1, XP_001900955.1, XP_002072681.1, ACO61833.1, XP_001661214.1, XP_001388880.1, CAO44082.1, XP_002103660.1, CAF94327.1, XP_002031418.1, EED17429.1, EEH09198.1, EEH22234.1, XP_551895.3, EEH58987.1, XP_001418083.1, YP_001549435.1, XP_001579763.1, AAX33507.1, NP_524333.1, NP_001022911.1, XP_002147292.1, ZP_04492974.1, XP_369564.2, CAX43655.1, YP_001325010.1, YP_564940.1, ZP_01547488.1, XP_001785063.1, YP_001116803.1, YP_001584677.1, XP_001666463.1, XP_447128.1, XP_001527108.1, XP_970333.2, XP_721056.1, YP_448202.1, NP_498250.3, XP_001944604.1, EEF37684.1, XP_002273685.1, CAO14730.1, NP_275798.1, XP_001745241.1, XP_001299980.1, XP_001316881.1, YP_001031006.1, YP_518145.1, YP_002459532.1, XP_646493.1, EEH57383.1, XP_001737507.1, XP_655520.1, YP_447162.1, XP_795220.2, XP_001194731.1, NP_248116.1, YP_001324295.1, NP_988010.1, NP_988010.1, YP_001549808.1, YP_001549808.1, YP_001097223.1, YP_001097223.1, YP_001329359.1, YP_001329359.1, YP_001322557.1, YP_001322557.1, YP_001097458.1, YP_001097458.1, YP_001097792.1, YP_001097792.1, YP_001548609.1, YP_001548609.1, ABD17736.1, ABD17736.1, ZP_02192479.1, ZP_02192479.1, YP_183745.1, YP_183745.1, YP_001405615.1, YP_001405615.1, NP_280985.1, NP_280985.1, YP_002580831.1, YP_002580831.1, NP_126564.1, NP_126564.1, YP_685308.1, YP_685308.1, ZP_03998706.1, YP_137330.1, NP_143168.1, NP_143168.1, NP_632449.1, NP_632449.1, YP_002567343.1, NP_578406.1, NP_578406.1, NP_618094.1, NP_618094.1, YP_565780.1, YP_565780.1, YP_002307730.1, YP_002307730.1, YP_843229.1, YP_843229.1, YP_002583268.1, YP_002583268.1, YP_656795.1, YP_656795.1, ZP_03875384.1, ZP_03875384.1, YP_325906.1, YP_325906.1, YP_306959.1, YP_306959.1, YP_001048404.1, YP_001048404.1, ZP_03692258.1, ZP_03692258.1, 2P6R, 2P6R, NP_071282.1, NP_071282.1, YP_503885.1, YP_503885.1, YP_919908.1, YP_919908.1, YP_002577078.1, YP_002577078.1, YP_001040230.1, YP_002579102.1, YP_002579102.1, YP_002467772.1, YP_002467772.1, NP_376477.1, NP_376477.1, YP_001540156.1, YP_254972.1, YP_001581577.1, YP_001435870.1, YP_001435870.1, Q97VY9.1, NP_343811.1, YP_002831080.1, YP_002913571.1, YP_002841682.1, ZP_03997680.1, YP_001191456.1, YP_001191456.1, YP_002828439.1, YP_930665.1, NP_613398.1, ABZ07376.1, ABZ07376.1, YP_002565871.1, YP_876638.1, YP_001054984.1, NP_558924.1, YP_001793507.1, YP_001152379.1, NP_111333.1, NP_147034.2, NP_147034.2, NP_394295.1, YP_001031179.1, YP_024158.1, ZP_03871982.1, ABZ08606.1, XP_973059.2, YP_002428409.1, XP_001661818.1, XP_002245656.1, XP_001661214.1, YP_001522732.1, XP_001850567.1, YP_002567151.1, XP_551895.3, EEB12036.1, XP_970333.2, CAM14344.1, XP_001919555.1, NP_125746.1, YP_002307364.1, NP_248404.1, YP_182979.1, YP_001329748.1, YP_002580586.1, YP_001096840.1, NP_988404.1, XP_001371040.1, EED17429.1, EEH22234.1, ABC72356.1, YP_001549435.1, ZP_03875402.1, YP_002583084.1, XP_001104832.1, EEH58987.1, NP_598375.2, Q8TDG4.1, XP_369564.2, ACO61833.1, XP_002147292.1, ZP_03524453.1, YP_656834.1, XP_001651546.1, XP_847166.1, CAX43655.1, XP_420565.1, XP_001418083.1, XP_001388880.1, XP_001944604.1, NP_614118.1, XP_721056.1, XP_001212516.1, YP_001323112.1, CAL55548.1, XP_796097.2, ZP_04492974.1, XP_001268360.1, NP_001022911.1, XP_001666463.1, NP_189410.2, YP_564940.1, YP_001325010.1, YP_001510960.1, NP_275798.1, YP_001031006.1, EEH57383.1, CAO14730.1, XP_001317120.1, YP_001584677.1, YP_001116803.1, NP_143015.1, XP_660343.1, ACO69496.1, XP_001419134.1, XP_447128.1, XP_002273685.1, XP_745111.1, YP_061841.1, EEF37684.1, XP_002258275.1, XP_002281624.1, XP_001563861.1, YP_001020869.1, XP_001893527.1, NP_648818.3, XP_001024630.1, XP_641869.1, ZP_03271562.1, AAO31691.1, ZP_03154204.1, ZP_01624095.1, ZP_01624095.1, NP_440867.1, NP_440867.1, YP_720719.1, YP_720719.1, CAO86769.1, CAO86769.1, YP_001660407.1, YP_001660407.1, YP_001803945.1, YP_001803945.1, YP_002618279.1, YP_002618279.1, ZP_00516710.1, ZP_00516710.1, ZP_01729993.1, ZP_01729993.1, ZP_03768651.1, YP_322728.1, YP_322728.1, YP_002481184.1, YP_002481184.1, YP_001867444.1, YP_001867444.1, NP_488972.1, NP_488972.1, ZP_01632292.1, ZP_01632292.1, YP_171340.1, YP_171340.1, YP_002379671.1, YP_002379671.1, YP_001734949.1, YP_001734949.1, ZP_03145654.1, ZP_03145654.1, YP_001516616.1, YP_001516616.1, YP_002710985.1, YP_002710985.1, NP_681922.1, NP_681922.1, YP_002372262.1, YP_002372262.1, YP_474042.1, YP_477541.1, NP_924555.1, NP_924555.1, NP_893892.1, NP_893892.1, YP_729300.1, YP_729300.1, ZP_01124809.1, ZP_01124809.1, YP_001016086.1, YP_001016086.1, ZP_01471607.1, ZP_01471607.1, ZP_01079138.1, ZP_01079138.1, ZP_01469011.1, ZP_01469011.1, NP_896156.1, NP_896156.1, YP_380396.1, YP_380396.1, YP_376076.1, YP_376076.1, YP_292449.1, YP_292449.1, YP_001015947.1, YP_001015947.1, YP_001223785.1, YP_001223785.1, NP_876209.1, NP_876209.1, YP_002049085.1, YP_002049085.1, YP_001226319.1, YP_001226319.1, YP_001551669.1, YP_001551669.1, ZP_01084450.1, ZP_01084450.1, YP_001010257.1, YP_398247.1, YP_002672750.1, YP_001092072.1, YP_002598469.1, YP_001485130.1, NP_893775.1, YP_001012162.1, YP_002380895.1, NP_478217.1, NP_478217.1, YP_320354.1, YP_320354.1, YP_428910.1, ZP_03473421.1, YP_001038541.1, NP_976348.1, ZP_04298477.1, ZP_04166762.1, YP_001642915.1, YP_001741277.1, NP_240900.1, ZP_04431303.1, ZP_04444970.1, ZP_03209476.1, YP_460125.1, YP_460125.1, ZP_02204431.1, YP_001214022.1, YP_001214022.1, ZP_03989453.1, ZP_03989453.1, CAJ74490.1, ZP_03042228.1, ZP_02691852.1, YP_002522958.1, YP_181327.1, YP_001679754.1, ZP_02850414.1, YP_307663.1, YP_307663.1, ZP_03644392.1, NP_690950.1, ZP_03489308.1, YP_002509819.1, YP_002568321.1, YP_001634159.1, ZP_02172071.1, YP_001275878.1, NP_810523.1, ZP_03128589.1, YP_001621369.1, ZP_03056671.1, YP_001485771.1, YP_355804.1, YP_355804.1, ZP_01311057.1, YP_001432394.1, YP_001432394.1, YP_002380962.1, YP_002769545.1, YP_001546684.1, ZP_03493305.1, ZP_03307119.1, YP_394949.1, ZP_03845813.1, ZP_01961228.1, ZP_01874827.1, ZP_01874827.1, NP_621748.1, YP_302368.1, YP_002596126.1, YP_002251788.1, ZP_04496402.1, ZP_04496402.1, NP_802084.1, YP_002464493.1, ZP_01885691.1, ZP_04058881.1, ZP_03855352.1, ZP_03855352.1, ZP_03955638.1, ZP_03807971.1, NP_269470.1, YP_001210601.1, YP_001210601.1, YP_001409800.1, YP_002246820.1, YP_254449.1, ABI26342.1, ZP_03975210.1, YP_001988369.1, YP_001270933.1, ZP_03224762.1, ZP_03460915.1, ZP_02067342.1, YP_002431162.1, NP_664842.1, ZP_04598865.1, ZP_04598865.1, ZP_04327004.1, YP_001716232.1, ZP_04543409.1, YP_001877834.1, YP_001128312.1, YP_598791.1, ZP_01048111.1, ZP_02862451.1, ZP_02862451.1, YP_002535848.1, YP_001233043.1, YP_002352197.1, YP_100510.1, YP_522721.1, YP_001916202.1, YP_001916202.1, ZP_02433909.1, NP_599495.2, YP_212684.1, YP_001243116.1, YP_575812.1, ZP_04048134.1, YP_316988.1, ZP_04581141.1, ZP_04479256.1, YP_002722088.1, YP_545681.1, YP_002671180.1, NP_214275.1, YP_001202926.1, YP_002290512.1, YP_783712.1, ZP_03857424.1, YP_361464.1, YP_001661692.1, ZP_03631092.1, ZP_03633677.1, YP_001666158.1, YP_073842.1, NP_951156.1, YP_002562521.1, ZP_03026382.1, YP_995313.1, YP_002137039.1, ZP_02883496.1, ZP_03544029.1, YP_615849.1, ZP_03734955.1, YP_479387.1, YP_001791008.1, YP_970990.1, YP_002240028.1, YP_001489229.1, YP_001175685.1, AAO31691.1, ZP_03271562.1, ZP_03154204.1, ZP_01624095.1, ZP_01624095.1, NP_440867.1, NP_440867.1, YP_720719.1, YP_720719.1, CAO86769.1, CAO86769.1, YP_001660407.1, YP_001660407.1, YP_001803945.1, YP_001803945.1, YP_002618279.1, YP_002618279.1, ZP_00516710.1, ZP_00516710.1, ZP_01729993.1, ZP_01729993.1, ZP_03768651.1, YP_322728.1, YP_322728.1, YP_002481184.1, YP_002481184.1, YP_001867444.1, YP_001867444.1, NP_488972.1, NP_488972.1, ZP_01632292.1, ZP_01632292.1, YP_171340.1, YP_171340.1, YP_002379671.1, YP_002379671.1, YP_001734949.1, YP_001734949.1, ZP_03145654.1, ZP_03145654.1, YP_001516616.1, YP_001516616.1, NP_681922.1, NP_681922.1, YP_002710985.1, YP_002710985.1, YP_002372262.1, YP_002372262.1, YP_474042.1, YP_477541.1, NP_924555.1, NP_924555.1, NP_893892.1, NP_893892.1, YP_729300.1, YP_729300.1, ZP_01124809.1, ZP_01124809.1, YP_001016086.1, YP_001016086.1, ZP_01471607.1, ZP_01471607.1, ZP_01079138.1, ZP_01079138.1, ZP_01469011.1, ZP_01469011.1, NP_896156.1, NP_896156.1, YP_380396.1, YP_380396.1, YP_376076.1, YP_376076.1, YP_292449.1, YP_292449.1, YP_001223785.1, YP_001223785.1, YP_001015947.1, YP_001015947.1, YP_002049085.1, YP_002049085.1, NP_876209.1, NP_876209.1, YP_001226319.1, YP_001226319.1, YP_001551669.1, YP_001551669.1, ZP_01084450.1, ZP_01084450.1, YP_001010257.1, YP_398247.1, YP_002672750.1, YP_001092072.1, YP_002598469.1, YP_001485130.1, NP_893775.1, YP_001012162.1, YP_002380895.1, NP_478217.1, NP_478217.1, YP_320354.1, YP_320354.1, NP_976348.1, ZP_04166762.1, ZP_04298477.1, YP_001038541.1, YP_001642915.1, YP_428910.1, ZP_03473421.1, YP_001741277.1, NP_240900.1, ZP_04431303.1, ZP_04444970.1, ZP_03209476.1, YP_460125.1, YP_460125.1, ZP_02204431.1, YP_001214022.1, YP_001214022.1, ZP_02691852.1, ZP_03644392.1, ZP_03989453.1, ZP_03989453.1, CAJ74490.1, ZP_03042228.1, YP_002522958.1, YP_181327.1, NP_690950.1, ZP_02850414.1, YP_307663.1, YP_307663.1, YP_001679754.1, ZP_03489308.1, YP_002509819.1, ZP_02172071.1, NP_810523.1, ZP_03128589.1, YP_002568321.1, YP_001634159.1, YP_001275878.1, ZP_03307119.1, ZP_03493305.1, YP_355804.1, YP_001621369.1, ZP_03056671.1, YP_001485771.1, ZP_01961228.1, ZP_01311057.1, NP_621748.1, YP_002596126.1, YP_001546684.1, YP_002769545.1, ZP_03845813.1, YP_394949.1, ZP_01885691.1, ZP_01874827.1, ZP_01874827.1, YP_302368.1, ZP_03460915.1, YP_002251788.1, YP_002431162.1, NP_802084.1, ZP_04496402.1, ZP_04496402.1, ZP_02067342.1, YP_002464493.1, ZP_03855352.1, ZP_03855352.1, ZP_04058881.1, ZP_04327004.1, ZP_03955638.1, ZP_03807971.1, NP_269470.1, YP_001877834.1, YP_001409800.1, YP_001409800.1, YP_001210601.1, YP_001210601.1, ZP_01048111.1, ZP_04543409.1, YP_002246820.1, ZP_03975210.1, ABI26342.1, YP_254449.1, YP_001988369.1, YP_807449.1, YP_001270933.1, NP_664842.1, ZP_04598865.1, ZP_04598865.1, YP_100510.1, YP_001916202.1, YP_001916202.1, YP_001716232.1, YP_212684.1, YP_001128312.1, ZP_02433909.1, YP_598791.1, YP_001243116.1, YP_575812.1, YP_001233043.1, YP_316988.1, YP_002535848.1, YP_002352197.1, YP_522721.1, YP_002671180.1, YP_001202926.1, NP_599495.2, YP_002290512.1, ZP_02862451.1, ZP_02862451.1, YP_783712.1, ZP_04048134.1, ZP_04581141.1, YP_002722088.1, YP_545681.1, ZP_04479256.1, NP_214275.1, YP_361464.1, YP_001661692.1, ZP_03633677.1, YP_001666158.1, YP_073842.1, ZP_03631092.1, NP_951156.1, YP_001785357.1, ZP_02619645.1, YP_001389393.1, ZP_02615807.1, YP_001252579.1, YP_002562521.1, ZP_03026382.1, YP_995313.1, YP_002137039.1, ZP_03734955.1, ZP_03544029.1, YP_615849.1, YP_479387.1, YP_001791008.1, YP_001432394.1, YP_970990.1, YP_002240028.1, YP_001489229.1, YP_001175685.1, NP_440867.1, YP_002481184.1, YP_002481184.1, ZP_03154204.1, ZP_03154204.1, YP_001803945.1, YP_001803945.1, ZP_00516710.1, ZP_00516710.1, ZP_03271562.1, ZP_03271562.1, AAO31691.1, AAO31691.1, ZP_01729993.1, ZP_01729993.1, CAO86769.1, CAO86769.1, YP_001660407.1, YP_001660407.1, ZP_01624095.1, ZP_01624095.1, ZP_03145654.1, ZP_03145654.1, YP_002372262.1, YP_002372262.1, YP_171340.1, YP_171340.1, NP_488972.1, NP_488972.1, YP_322728.1, YP_322728.1, YP_002618279.1, YP_002618279.1, ZP_03768651.1, YP_001734949.1, YP_001734949.1, YP_002379671.1, YP_002379671.1, YP_001867444.1, YP_001867444.1, ZP_01632292.1, ZP_01632292.1, YP_720719.1, YP_720719.1, YP_002710985.1, YP_002710985.1, NP_681922.1, NP_681922.1, YP_001516616.1, YP_001516616.1, YP_477541.1, YP_477541.1, YP_474042.1, YP_474042.1, NP_924555.1, NP_924555.1, YP_729300.1, YP_729300.1, ZP_01471607.1, ZP_01471607.1, NP_893892.1, NP_893892.1, YP_001015947.1, YP_001015947.1, YP_292449.1, YP_292449.1, YP_001016086.1, YP_001016086.1, ZP_01469011.1, ZP_01469011.1, ZP_01124809.1, ZP_01124809.1, ZP_01079138.1, ZP_01079138.1, YP_001226319.1, YP_001226319.1, YP_002598469.1, YP_002598469.1, YP_002049085.1, YP_002049085.1, YP_001223785.1, YP_001223785.1, NP_896156.1, NP_896156.1, YP_001551669.1, YP_376076.1, YP_380396.1, ZP_01084450.1, YP_002380895.1, YP_002380895.1, YP_001012162.1, ZP_02691852.1, YP_320354.1, YP_320354.1, NP_478217.1, NP_478217.1, ZP_04014269.1, ZP_02914854.1, ZP_03473421.1, YP_615849.1, YP_002380962.1, YP_002380962.1, YP_002509819.1, NP_784458.1, YP_460125.1, NP_901280.1, YP_001546684.1, YP_496635.1, YP_001124148.1, ZP_01865109.1, ZP_01865109.1, ZP_03149856.1, YP_002769545.1, YP_002769545.1, YP_002522958.1, YP_002522958.1, YP_457625.2, ZP_01039165.1, ZP_01039165.1, YP_002568321.1, CAJ74490.1, ZP_04444970.1, YP_001634159.1, ZP_03989453.1, YP_428910.1, ZP_01311057.1, ZP_04431303.1, ZP_03698959.1, YP_001642915.1, ZP_03224762.1, ZP_03224762.1, YP_002794964.1, ZP_04166762.1, ZP_04195306.1, ZP_04353963.1, ZP_04353963.1, ZP_04292810.1, YP_002314386.1, YP_002464493.1, NP_802084.1, ZP_03857424.1, YP_173541.1, YP_173541.1, YP_001741277.1, YP_002246820.1, ZP_04601111.1, ZP_04601111.1, ZP_01874827.1, ZP_01874827.1, ZP_03056671.1, YP_001409800.1, NP_240900.1, ZP_01304049.1, ZP_01304049.1, YP_002905481.1, YP_002886082.1, YP_001485771.1, ZP_02184382.1, NP_245301.1, ZP_03115095.1, ZP_02189420.1, ZP_02189420.1, YP_002535848.1, YP_088063.1, ZP_03712815.1, YP_591050.1, YP_591050.1, NP_765862.1, ZP_03613453.1, ZP_02862451.1, ZP_01861164.1, ZP_03042228.1, ZP_01173799.1, ZP_03940736.1, ZP_03943752.1, ZP_04496402.1, NP_599495.2, NP_599495.2, YP_545681.1, YP_089706.1, YP_001679754.1, YP_002619251.1, YP_001233043.1, ZP_01749222.1, YP_001854136.1, YP_001854136.1, ZP_01786631.1, CAA29958.1, NP_387900.1, ZP_03589675.1, P09122.2, ZP_03074096.1, YP_794776.1, ZP_04009013.1, ZP_03959564.1, YP_386562.1, YP_386562.1, YP_536119.1, ZP_03975210.1, ABI26342.1, ZP_03757089.1, YP_288112.1, ZP_03715825.1, ZP_01003372.1, YP_002730323.1, ZP_03949486.1, NP_816407.1, ZP_04433563.1, YP_001102527.1, ZP_03212434.1, ZP_04441669.1, ZP_01725093.1, YP_001270933.1, YP_001501368.1, YP_001695809.1, YP_001877834.1, YP_001877834.1, ZP_03663908.1, ZP_02001285.1, YP_001950613.1, YP_001419699.1, YP_807449.1, YP_001988369.1, ZP_03958202.1, YP_001843099.1, ZP_03944075.1, ZP_03845813.1, YP_394949.1, YP_355804.1, YP_804957.1, ZP_04598865.1, ZP_03855352.1, YP_645183.1, ZP_02868558.1, ZP_03893734.1, ZP_00785568.1, ZP_00998654.1, YP_002513147.1, ZP_01741705.1, ZP_04449652.1, ZP_03633677.1, YP_511818.1, YP_073842.1, YP_002251788.1, ZP_02144661.1, YP_002704707.1, ZP_02147741.1, ZP_04563919.1, YP_630176.1, ZP_02140144.1, YP_681396.1, ZP_02427263.1, ZP_01034941.1, YP_001791008.1, YP_001166732.1, YP_002352197.1, YP_002690699.1, YP_251812.1, YP_116490.1, ZP_03883707.1, ZP_03734955.1, YP_903693.1, YP_390799.1, ZP_01160007.1, ZP_01235825.1, YP_001801306.1, YP_001260566.1, YP_704148.1, XP_001122770.1, YP_001701062.1, YP_907800.1, YP_956306.1, YP_162596.2, YP_001853500.1, ZP_03994594.1, ZP_04606237.1

DNA-directed DNA polymerase:

**DNA-directed RNA polymerase subunit B:** YP_635949.1, ABU88332.1, ACI31271.1, NP_958397.1, ACS95102.1, YP_002000391., YP_764412.1, ABU88269.1, YP_001382217., YP_635631.1, YP_538840.1, Q2VEI4.1, YP_514844.1, NP_054488.1, YP_398851.1, NP_783224.1, YP_740109.1, ABU85190.1, YP_086958.1, ACC93301.1, YP_762253.1, Q4VZP1.2, YP_247591.1, P46818.2, CAA57814.1, YP_001123365., NP_051051.1, YP_001123454., YP_001123278., YP_001123806., YP_001123629., YP_001123107., YP_001123191., YP_001123542., YP_001123717., YP_001123022., YP_001122938., YP_001671675., YP_538927.1, ACF33355.1, YP_665550.1, YP_001109491., YP_001718429., YP_002720104., YP_740467.1, NP_054924.1, Q589C0.1, NP_084686.2, YP_001687282., YP_001687136., YP_001468300., ACC93305.1, ACC93376.1, ACC93362.1, ACC93356.1, ACC93371.1, ACC93369.1, ACC93304.1, ACC93363.1, ACC93391.1, ACC93302.1, ACC93320.1, ACC93340.1, ACC93384.1, ACC93331.1, ACC93353.1, ACC93344.1, ACC93332.1, ACC93312.1, ACC93327.1, ACC93323.1, ACC93333.1, NP_084796.1, YP_002149733., AAL07334.1, YP_636291.1, YP_567068.1, YP_001294176., YP_740557.1, YP_740194.1, Q0G9M7.2, YP_784378.1, YP_001294090., Q7YJX8.1, NP_862746.2, YP_001004178., YP_002836082., YP_740642.1, YP_001542439., YP_001294262., A6MMT6.2, NP_904091.2, CAD45099.1, YP_001001526., YP_053147.1, ABU85436.1, AAZ03995.1, ABU85343.1, AAZ03996.1, YP_001294344., YP_001586174., YP_319757.1, Q3V542.2, AAZ03991.1, Q5QA72.1, YP_001595500., YP_784465.1, YP_358570.2, AAW82495.1, P08036.1, ACH47354.1, ACH47356.1, ACC93341.1, ACG69790.1, AAY58045.1, YP_002519540., ACP52193.1, ACP51394.1, ACP51891.1, ACP51642.1, YP_002905075., NP_039275.1, NP_904223.1, YP_001023692., YP_002970636., Q85FM7.2, NP_683774.1, YP_636566.1, YP_635874.1, ACQ90798.1, ACQ90878.1, YP_636174.1, ZP_01630667.1, ZP_03766028.1, NP_485634.1, YP_324701.1, YP_001868272., YP_002371954., YP_002376065., YP_722576.1, YP_173217.1, YP_400539.1, ZP_05037107.1, NP_681431.1, YP_473891.1, YP_478633.1, YP_001015706., YP_292209.1, NP_038385.1, YP_001019104., YP_063642.1, YP_002600950., NP_050839.1, YP_002808499., YP_002808641., YP_003058290., NP_045893.1, YP_778610.1, YP_638140.1, YP_936991.1, YP_001069296., AAN65352.1, AAN65360.1, AAQ76597.1, AAQ76601.1, AAN65359.1, AAQ76596.1, AAX93862.1, AAN65353.1, AAN65354.1, AAQ76602.2, YP_885753.1, AAQ76594.1, AAQ76595.1, AAN65355.1, YP_952098.1, A1T4J2.2, AAT86005.2, AAX93859.1, AAQ76598.1, YP_001704597., AAQ76600.1, AAN65350.1, AAQ76599.1, AAN65357.1, AAN65361.1, AAN65358.1, AAX93860.1, NP_963064.1, Q73SE4.2, YP_883637.1, ZP_05218454.1, ZP_05227774.1, YP_904864.1, YP_001849306., ZP_04746327.1, YP_001286626., NP_335107.1, A5U052.2, NP_215181.1, ZP_05140074.1, ZP_05327607.1, ZP_02549763.1, ZP_03423823.1, NP_302273.1, ABW91171.2, YP_002765180., ZP_04384573.1, YP_002778832., YP_121315.1, ZP_03886746.1, ACD63067.1, YP_003104270., YP_001108943., ZP_05476788.1, Q9L637.1, YP_003132284., YP_003200460., YP_001160741., YP_001539097., ZP_04605839.1, ZP_03890901.1, YP_001137461., YP_224788.1, NP_599733.1, NP_737107.1, ZP_03979859.1, EEW15876.1, AAS89193.1, YP_251643.1, YP_001799694., AAS89198.1, YP_711331.1, YP_479686.1, YP_001510307., ZP_04473865.1, AAV33242.1, AAV33243.1, ZP_04029194.1, ZP_04331925.1, YP_290710.1, Q82DQ5.2, NP_826091.1, ACR48347.1, ZP_04686482.1, ZP_05016391.1, ZP_05533594.1, NP_628815.1, ZP_05524297.1, ZP_05539406.1, ZP_04994617.1, ZP_05489113.1, ZP_04695503.1, ZP_04710683.1, YP_001824381., EEW74315.1, ZP_05006608.1, ZP_05012975.1, ZP_05515691.1, ZP_04998535.1, ZP_05508376.1, ZP_04702597.1, YP_003111690., YP_872059.1, YP_921923.1, ZP_03860040.1, YP_001360433., YP_003149854., ZP_00379579.1, ZP_03911407.1, ZP_04366331.1, YP_003160547., YP_002883161., YP_003155775., YP_832463.1, YP_002488751., YP_948666.1, YP_001625332., YP_002957766., YP_001854454., ZP_05367909.1, YP_001223376., YP_001709051., YP_062874.1, ZP_01129377.1, ZP_03389755.1, YP_056565.1, ZP_03924853.1, YP_003109032., ZP_01772071.1, ZP_03296715.1, YP_003144354., YP_644918.1, ZP_02954870.1, NP_563329.1, YP_699671.1, ZP_02865015.1, YP_001884466., YP_001919665., ZP_02950121.1, ZP_05129518.1, YP_001392842., ZP_02613488.1, YP_001255970., YP_001389211., YP_001783129., ZP_02993041.1, ZP_02616520.1, YP_001788829., NP_783125.1, NP_349741.1, ZP_04807642.1, YP_877190.1, YP_001039116., ZP_05428124.1, YP_001664371., YP_001662497., ZP_05334523.1, ZP_05380142.1, NP_623839.1, ZP_05337282.1, YP_001322217., YP_001179756., YP_002572692., ZP_01967712.1, ZP_02040802.1, ZP_03288719.1, ZP_02430634.1, ZP_04745576.1, ZP_04671566.1, ZP_02421977.1, YP_001692426., ZP_04018708.1, ZP_03916666.1, YP_003152214., ZP_02862401.1, YP_001111581., YP_003189854., Q250P0.2, YP_516696.1, YP_002456917., YP_001679958., AAN87431.1, YP_431293.1, YP_361127.1, YP_001716409., ZP_01666572.1, YP_002507865., YP_001916373., ZP_03729676.1, YP_076911.1, YP_755003.1, ZP_03928902.1, ZP_04659482.1, YP_804905.1, YP_001728816., YP_810902.1, ZP_03955718.1, ZP_05557415.1, ZP_04645388.1, ZP_04676106.1, ZP_05743910.1, ZP_01862157.1, ZP_03224894.1, YP_001419786., ZP_03589766.1, YP_077392.1, YP_002948309., ZP_05373236.1, NP_240992.1, YP_173646.1, ZP_02171803.1, ACV96839.1, ACV96841.1, ACV96840.1, ZP_03494190.1, YP_003186112., YP_002561269., YP_302303.1, ZP_05736808.1, ZP_02868682.1, YP_002522208., ZP_04494441.1, ZP_05734194.1, ZP_04339719.1, ZP_04468429.1, YP_709836.1, ZP_03435858.1, ZP_03675215.1, YP_002374906., ZP_03673607.1, ZP_03623495.1, ZP_03436875.1, NP_212523.1, ZP_03086643.1, ZP_03773170.1, ZP_03539597.1, ZP_03540264.1, YP_072834.1, ZP_03672435.1, ZP_03796107.1, AAC37118.1, YP_945389.1, YP_001883818., YP_002222041., YP_002222852., NP_218681.1, ZP_05623332.1, NP_973020.1, YP_002722483., ZP_04048291.1, NP_953905.1, YP_383586.1, YP_900362.1, YP_356123.1, YP_002364112., ZP_04571404.1, ZP_00143866.1, ZP_05552218.1, NP_602822.1, ZP_04969648.1, ZP_04573555.1, ZP_04569297.1, ZP_04858865.1, ZP_05634115.1, ZP_04566512.1, ZP_05618206.1, ZP_05631023.1, ZP_05626968.1, ZP_04504634.1, YP_003164748., ZP_04480012.1, ZP_02930291.1, YP_001956018., YP_001930497., YP_001305754., YP_002335131., YP_001411272., YP_001470108., YP_002941524., YP_001567420., ABF97884.1, EEE59590.1, XP_002466750., Q42877.1, AAY89346.1, AAY89347.1, XP_002519060., AAZ08457.1, AAY89348.1, AAZ08467.1, CAO69734.1, XP_002274051., CAA79528.1, CAA79527.1, NP_193902.1, AAY89343.1, AAZ08458.1, AAY89344.1, AAY89345.1, XP_001766425., CAL50081.1, XP_001415447., EEH59470.1, XP_002500540., XP_636812.1, ABF71504.1, ABF71517.1, AAU86990.1, ABF71505.1, ABF71506.1, ABF71525.1, ABF71522.1, ABF71529.1, AAU86988.1, AAU87001.1, AAU86974.1, ABL85615.1, AAS67518.1, ABG81893.1, AAU86981.1, AAS67523.1, AAU86985.1, AAS67521.1, AAU87006.1, ABG81897.1, XP_001552822., AAR85529.1, AAF19080.1, AAF19078.1, AAF19079.1, AAU86970.1, AAU86971.1, XP_001209185., XP_001825469., XP_001395161., AAS67499.1, XP_746740.1, XP_001262829., XP_001272355., XP_002568295., XP_001240650., EER25746.1, EEQ29571.1, EEQ78654.1, EEQ86645.1, XP_001540601., EEH10902.1, EEH18811.1, EEH36436.1, XP_002153141., AAF19081.1, AAF19082.1, AAS67514.1, AAF19077.1, AAS67505.1, AAF19075.1, AAF19074.1, AAF19071.1, AAF19072.1, XP_001226434., AAF19063.1, XP_001903788., AAF19062.1, XP_957106.2, CAD70445.1, AAS67508.1, ACQ84444.1, EEU39607.1, AAF19064.1, EDK38735.2, A5DHT2.3, XP_001485104., AAS67502.2, EEQ39501.1, CAR65929.1, XP_001387366., XP_001526602., AAF19059.1, XP_718439.1, XP_002416823., XP_002550158., AAS67504.1, XP_002491011., AAA68096.1, NP_014794.1, XP_448959.1, XP_001642238., XP_002496945., NP_985951.1, XP_451784.1, XP_002555168., XP_502376.1, BAA02600.1, NP_593101.2, XP_002175152., AAS67520.1, AAS67522.1, AAF19058.1, ABF18579.1, ABF18581.1, ABF18580.1, ABF06597.1, ABF18585.1, AAS67498.1, AAV48865.1, AAV53580.1, AAV48869.1, AAV48871.1, AAV53579.1, XP_001873382., AAV53367.1, ABD59032.1, ABF18587.2, ABF18576.1, ABF18586.1, AAS57791.1, AAQ01544.3, ABD59037.1, AAO64666.2, ABD59039.1, AAV48863.1, AAF19057.1, AAZ14928.1, XP_001829140., AAV48868.1, AAV53371.1, AAV53365.1, ABD04081.1, AAV53372.1, AAV53578.1, AAO64656.2, AAO64677.2, AAW80835.1, AAV48870.1, ABD65893.1, AAV48864.1, AAV53362.1, ABD65888.1, AAV53363.1, ABD65889.1, ABD65892.1, ABD65886.1, ABD65890.1, ABD65891.1, AAV53364.1, AAV53370.1, ABD46531.1, ABF67584.1, ABD65902.1, AAS67513.1, ABD46525.1, ABD46526.1, ABD04083.1, ABD59028.1, AAO64654.3, XP_775871.1, XP_570204.1, ABG88855.1, AAS67519.1, ABB20921.1, ABG29164.1, ABG53982.1, ABG53986.1, ABG72747.1, ABG88065.1, ABI17423.1, ABH09761.2, XP_001731621., ABH07989.1, ABM27006.1, ABC17947.1, ABM27007.1, ABM27008.1, ABC17948.1, ABC17955.1, ABC17950.1, ABC17931.1, ABC17956.1, ABC17940.1, ABC17944.1, ABC17943.1, ABM27009.1, ABC17952.1, ABC17941.1, ABM27011.1, ABM27012.1, ABM27013.1, ABC17945.1, ABC17959.1, ABC17949.1, ABM27010.1, ABC17942.1, ABM27018.1, ABC17938.1, ABC17934.1, ABC17958.1, ABC17939.1, ABC17951.1, ABM27022.1, ABM27023.1, ABM27014.1, ABM27015.1, XP_860737.1, XP_860838.1, XP_001083325., NP_000929.1, NP_722493.2, XP_001916826., NP_001092552., XP_860930.1, XP_860708.1, XP_001362455., BAG58155.1, AAH38472.1, XP_002192218., NP_001006448.1, XP_860990.1, XP_861018.1, XP_860865.1, XP_860899.1, XP_860963.1, XP_860777.1, XP_860805.1, XP_001136218., BAG57990.1, XP_861044.1, BAG61152.1, ACN10943.1, NP_001019632., CAF93098.1, XP_002128916., XP_002226834., XP_002228706., XP_313416.3, XP_001849183., NP_476706.1, XP_002103487., XP_001979888., XP_002097907., XP_002013620., XP_001955468., XP_002070673., XP_001996619., CAA29180.2, XP_001999501., XP_002054770., XP_002425513., XP_974653.1, XP_001607902., XP_001628960., XP_002163509., CAX73303.1, XP_002573029., XP_001744279., XP_763975.1, XP_953007.1, XP_001609729., XP_002141052., XP_628535.1, XP_002365045., EEQ99605.1, NP_586140.1, EEQ82318.1, XP_001827997., XP_001712221., XP_001713520., XP_001323594., XP_001467277., XP_001685000., XP_001567028., XP_844531.1, BAH80351.1, BAH80359.1, BAH80355.1, BAH80356.1, BAH80354.1, YP_659080.1, BAH80368.1, BAH80369.1, BAH80367.1, BAH80375.1, BAH80378.1, BAH80380.1, BAH80373.1, NP_281213.1, BAH80339.1, BAH80338.1, BAH80330.1, ZP_03692443.1, BAH80395.1, BAH80394.1, BAH80393.1, BAH80392.1, BAH80396.1, BAH80391.1, BAH80403.1, BAH80405.1, BAH80404.1, BAH80408.1, BAH80410.1, BAH80411.1, BAH80382.1, BAH80406.1, BAH80341.1, BAH80342.1, BAH80343.1, BAH80402.1, BAH80383.1, BAH80401.1, BAH80384.1, BAH80398.1, BAH80388.1, BAH80397.1, BAH80399.1, BAH80386.1, BAH80400.1, BAH80385.1, BAH80407.1, BAH80389.1, BAH80390.1, BAH80381.1, YP_325719.1, BAH80350.1, BAH80347.1, BAH80348.1, BAH80349.1, BAH80345.1, NP_616203.1, NP_634296.1, YP_307136.1, YP_842471.1, YP_684649.1, NP_070712.1, YP_001405091., YP_002467181., YP_001046215., YP_501548.1, YP_001030598., NP_111701.1, NP_393870.1, ZP_05570967.1, YP_023037.1, ZP_04875898.1, YP_001096747., YP_001549356., YP_001329825., NP_988482.1, NP_126305.1, NP_143407.1, NP_579293.1, YP_002306599., P31814.1, YP_002960292., ZP_04879274.1, YP_183496.1, ZP_04878049.1, YP_002993803., ABZ07549.1, NP_613971.1, YP_001795324., NP_558752.1, YP_001056972., YP_001154517., YP_930224.1, YP_001540284., YP_919699.1, YP_001013739., NP_148216.1, YP_001435240., YP_001040811., YP_002429068., CAA32924.1, YP_255366.1, NP_376135.1, YP_002832660., YP_002838165., YP_002829953., 2WAQ, ZP_04766297.1, YP_001190134., YP_876491.1, YP_001581681., ABZ08819.1, YP_001737952., XP_343189.3, NP_081699.1, XP_001499030., XP_531760.2, NP_060552.4, AAM18214.1, CAD97689.1, XP_613382.4, XP_001161529., XP_001365927., XP_001508364., XP_416307.2, XP_002196633., NP_001121521., XP_690811.2, CAG03415.1, XP_002233223., ACI49130.1, NP_498192.2, CAP20749.1, XP_001242390., EER27479.1, XP_002544581., EEH02917.1, EER40475.1, EEQ70841.1, EEQ90505.1, EEH37653.1, EEQ34008.1, XP_001388998., XP_002149081., EEU48715.1, XP_381019.1, XP_001483367., EDK39998.2, CAR66041.1, XP_002422092., XP_448895.1, NP_593690.1, XP_002171911., XP_758593.1, NP_001050323., AAP03366.1, XP_002436683., XP_002321356., CAL50515.1, XP_001416013., EEH52743.1, XP_002508452., XP_001755844., XP_002178910., XP_002286749., XP_654890.2, P42379.1, ABU88294.1, YP_001047160., YP_002467125

**FeS assembly protein:** ZP_03415495.1, NP_215977.1, YP_002161464.1, ZP_03424718.1, ZP_03432406.1, NP_960121.1, NP_960121.1, YP_882500.1, YP_882500.1, YP_001850570.1, YP_001850570.1, YP_905799.1, YP_905799.1, NP_301502.1, NP_301502.1, AAA17127.1, AAA17127.1, YP_953546.1, YP_953546.1, YP_001134939.1, YP_001134939.1, YP_887437.1, YP_887437.1, YP_001703482.1, YP_001703482.1, YP_639600.1, YP_639600.1, ZP_03884089.1, ZP_03884089.1, ZP_04028201.1, ZP_04028201.1, YP_119772.1, YP_119772.1, YP_707118.1, YP_707118.1, ZP_04506663.1, ZP_04506663.1, ZP_03818257.1, ZP_03818257.1, YP_002784176.1, YP_002784176.1, ZP_04349993.1, ZP_04349993.1, ZP_04375487.1, ZP_03892132.1, ZP_03892132.1, ZP_04604828.1, ZP_04604828.1, YP_002766502.1, YP_002766502.1, YP_002882353.1, YP_002882353.1, ZP_03393789.1, ZP_03393789.1, ZP_03868959.1, ZP_03868959.1, YP_002208058.1, YP_002208058.1, YP_001538104.1, YP_001538104.1, NP_626190.1, NP_626190.1, ZP_00995379.1, YP_250766.1, YP_250766.1, NP_600779.1, YP_001159898.1, YP_001159898.1, ZP_04476645.1, YP_001104405.1, YP_001104405.1, YP_872893.1, YP_872893.1, NP_827501.1, NP_827501.1, YP_002187615.1, YP_002187615.1, ZP_03909975.1, ZP_03909975.1, ZP_04529841.1, ZP_04529841.1, YP_002180553.1, ZP_04032700.1, YP_923741.1, YP_001506428.1, ZP_03866472.1, YP_001827107.1, YP_002197775.1, YP_002191375.1, YP_002191375.1, ZP_02944081.1, NP_738296.1, NP_738296.1, YP_001800401.1, YP_001855079.1, YP_831583.1, YP_001625384.1, YP_001625384.1, YP_002487910.1, YP_947848.1, YP_947848.1, ZP_04365785.1, YP_001362645.1, YP_001362645.1, YP_002906248.1, ZP_03979287.1, ZP_03921818.1, ZP_04484935.1, ZP_03710587.1, ZP_03933643.1, YP_002834747.1, ZP_03972179.1, ZP_03934424.1, ZP_03924432.1, ZP_03926597.1, ZP_03926597.1, ZP_03924943.1, ZP_03924943.1, ZP_04447975.1, ZP_03646243.1, ZP_03619046.1, ZP_02962923.1, YP_002470295.1, YP_062118.1, YP_001222474.1, YP_001710668.1, ZP_02044363.1, ZP_03432405.1, YP_002774335.1, NP_295829.1, YP_001199237.1, ZP_03625646.1, ZP_03875610.1, NP_971280.1, YP_001821042.1, NP_349883.1, YP_001191736.1, ZP_03462129.1, YP_002840577.1, NP_342417.1, YP_002829349.1, NP_377132.1, YP_256015.1, ACO63228.1, YP_002668436.1, YP_277870.1, YP_002417510.1, ZP_01066347.1, YP_002580918.1, NP_579015.1, CAL54755.1, NP_215977.1, ZP_03415495.1, YP_002161464.1, ZP_03424718.1, ZP_03432406.1, NP_960121.1, NP_960121.1, YP_882500.1, YP_882500.1, YP_001850570.1, YP_001850570.1, YP_905799.1, YP_905799.1, NP_301502.1, NP_301502.1, AAA17127.1, AAA17127.1, YP_953546.1, YP_953546.1, YP_001134939.1, YP_001134939.1, YP_887437.1, YP_887437.1, YP_001703482.1, YP_001703482.1, YP_639600.1, YP_639600.1, ZP_03884089.1, ZP_03884089.1, ZP_04028201.1, ZP_04028201.1, YP_119772.1, YP_119772.1, YP_707118.1, YP_707118.1, ZP_04506663.1, ZP_04506663.1, ZP_03818257.1, ZP_03818257.1, YP_002784176.1, YP_002784176.1, ZP_04349993.1, ZP_04349993.1, ZP_03892132.1, ZP_03892132.1, ZP_04375487.1, ZP_04604828.1, ZP_04604828.1, YP_002766502.1, YP_002766502.1, YP_002882353.1, YP_002882353.1, ZP_03393789.1, ZP_03393789.1, ZP_03868959.1, ZP_03868959.1, YP_001538104.1, YP_001538104.1, YP_002208058.1, YP_002208058.1, NP_626190.1, NP_626190.1, ZP_00995379.1, YP_250766.1, YP_250766.1, NP_600779.1, YP_001159898.1, YP_001159898.1, ZP_04476645.1, YP_001104405.1, YP_001104405.1, YP_872893.1, YP_872893.1, NP_827501.1, NP_827501.1, YP_002187615.1, YP_002187615.1, ZP_03909975.1, ZP_03909975.1, ZP_04529841.1, ZP_04529841.1, YP_002180553.1, ZP_04032700.1, YP_923741.1, YP_001506428.1, ZP_03866472.1, YP_001827107.1, YP_002197775.1, YP_002191375.1, YP_002191375.1, NP_738296.1, NP_738296.1, ZP_02944081.1, YP_001800401.1, YP_001855079.1, YP_831583.1, YP_001625384.1, YP_001625384.1, YP_002487910.1, YP_947848.1, YP_947848.1, ZP_04365785.1, YP_001362645.1, YP_001362645.1, YP_002906248.1, ZP_03979287.1, ZP_03921818.1, ZP_04484935.1, ZP_03710587.1, ZP_03933643.1, YP_002834747.1, ZP_03972179.1, ZP_03934424.1, ZP_03924432.1, ZP_03926597.1, ZP_03926597.1, ZP_03924943.1, ZP_03924943.1, ZP_04447975.1, ZP_03646243.1, ZP_03619046.1, ZP_02962923.1, YP_002470295.1, YP_062118.1, YP_001222474.1, YP_001710668.1, ZP_02044363.1, ZP_03432405.1, YP_002774335.1, NP_295829.1, YP_001199237.1, ZP_03625646.1, ZP_03875610.1, NP_971280.1, YP_001821042.1, NP_349883.1, YP_001191736.1, ZP_03462129.1, NP_342417.1, YP_002840577.1, YP_002829349.1, NP_377132.1, YP_256015.1, ACO63228.1, YP_002668436.1, YP_277870.1, YP_002417510.1, ZP_01066347.1, YP_002580918.1, NP_579015.1, CAL54755.1, ZP_03432406.1, ZP_03415495.1, NP_215977.1, YP_002161464.1, ZP_03424718.1, YP_001850570.1, YP_001850570.1, YP_905799.1, YP_905799.1, YP_882500.1, YP_882500.1, NP_960121.1, NP_960121.1, AAA17127.1, AAA17127.1, NP_301502.1, NP_301502.1, YP_953546.1, YP_001134939.1, YP_887437.1, YP_887437.1, YP_001703482.1, YP_001703482.1, YP_639600.1, YP_639600.1, ZP_04028201.1, YP_119772.1, YP_119772.1, ZP_03884089.1, ZP_03884089.1, ZP_04349993.1, ZP_03892132.1, ZP_03892132.1, ZP_04375487.1, YP_002784176.1, YP_002784176.1, YP_707118.1, YP_707118.1, ZP_03818257.1, YP_002766502.1, YP_002208058.1, YP_002208058.1, NP_626190.1, ZP_00995379.1, ZP_00995379.1, YP_001159898.1, ZP_04476645.1, YP_872893.1, YP_872893.1, YP_001538104.1, ZP_04506663.1, ZP_04506663.1, ZP_04604828.1, NP_827501.1, YP_002187615.1, YP_002180553.1, ZP_03393789.1, ZP_04032700.1, ZP_04032700.1, YP_923741.1, YP_923741.1, YP_001506428.1, ZP_03866472.1, YP_001827107.1, YP_002197775.1, YP_002191375.1, YP_001104405.1, ZP_02944081.1, YP_002882353.1, YP_002882353.1, YP_001855079.1, YP_831583.1, YP_001625384.1, YP_001625384.1, YP_002487910.1, YP_947848.1, YP_001362645.1, YP_001362645.1, ZP_04365785.1, ZP_04365785.1, YP_250766.1, NP_738296.1, ZP_03909975.1, ZP_03909975.1, ZP_03926597.1, ZP_04447975.1, ZP_04447975.1, ZP_03646243.1, ZP_04529841.1, ZP_04529841.1, ZP_03924943.1, ZP_03924943.1, YP_002470295.1, YP_001222474.1, YP_001222474.1, YP_001710668.1, YP_001710668.1, ZP_02044363.1, YP_002774335.1, ZP_03857858.1, YP_002521629.1, YP_001191736.1, YP_002840577.1, NP_342417.1, YP_002829349.1, NP_377132.1, YP_256015.1, YP_001378096.1, YP_002133254.1, YP_464055.1, YP_002668436.1, YP_277870.1, YP_002417510.1, ZP_01066347.1, ZP_03868959.1, ZP_04042079.1, YP_642960.1, ZP_04484935.1, ZP_03979287.1, YP_001800401.1, ZP_01130842.1, ZP_03937069.1, YP_062118.1, ZP_02028857.1, YP_909573.1, ZP_03447488.1, YP_001199237.1, ZP_03625646.1, ZP_03462129.1, NP_349883.1

**Filamentous hemagglutinin:** ZP_04613142.1, ZP_04634636.1, ZP_04628641.1, ZP_04619672.1, YP_646399.1, AAQ19126.1, YP_002934444., YP_002934701., NP_522634.1, ZP_00944934.1, YP_002255171., ZP_05726329.1, YP_002946398., YP_002948178., ZP_03741546.1, ZP_03543515.1, YP_544594.1, YP_001968808., ZP_00204494.1, YP_001651981., YP_001344309., NP_274768.1, YP_088359.1, CAR67544.1, YP_003041124., CAR67723.1, YP_003042136., YP_003041856., YP_003040322., YP_003039283., NP_928667.1, NP_930925.1, NP_928461.1, NP_927898.1, ZP_03828273.1, ZP_05305225.1, YP_002934700., AAQ19127.1, YP_002649872., ZP_03825513.1, ZP_03830383.1, YP_003017898., ZP_03317994.1, ZP_03320338.1, ZP_03806087.1, YP_001829746., YP_001830569., NP_779977.1, ZP_00681037.1, ZP_00650709.1, NP_298179.1, NP_299475.1, NP_300052.1, YP_001830888., NP_780288.1, ZP_00680623.1, ZP_02244867.1, ZP_02244872.1, YP_001971244., YP_002026501., ZP_02244874.1, YP_001915776., YP_728413.1, YP_294319.1, ZP_02381054.1, YP_001807094., NP_519896.1, YP_002255787., YP_001629204., ZP_05734200.1, YP_775667.1, ZP_02889734.1, ZP_04947042.1, ZP_02907909.1, NP_519008.1, NP_522101.1, YP_002255281., YP_002255280., YP_002257944., YP_001860454., YP_557393.1, ZP_03264850.1, YP_001863342., YP_558167.1, YP_002261122., YP_587469.1, ZP_04897245.1, YP_332688.1, YP_001065409., YP_001058172., ZP_01767991.1, ZP_04965784.1, ZP_03450969.1, YP_002895843., ZP_04887939.1, YP_001068101., ZP_02511815.1, ZP_02483697.1, ZP_02369415.1, YP_002909362., YP_443237.1, ZP_04945976.1, ZP_03570704.1, ZP_03575066.1, YP_001116438., ZP_02907825.1, ZP_02379482.1, ZP_04892296.1, YP_336327.1, ZP_04891150.1, ZP_04522526.1, YP_001076831., YP_112055.1, ZP_03454169.1, ZP_04968277.1, ZP_03789655.1, YP_001861848., YP_002005597., YP_002872763., YP_260790.1, ZP_05639281.1, NP_792996.1, YP_609437.1, XP_001786853., ZP_05639280.1, YP_258679.1, YP_258682.1, ZP_03397994.1, ZP_05639285.1, NP_793014.1, ZP_05639287.1, YP_002872819., YP_257313.1, ZP_05639282.1, YP_002869844., ZP_01362953.1, NP_248731.1, YP_002437651., YP_001345440., YP_788197.1, ZP_04931039.1, YP_001348137., YP_790775.1, YP_001348147., YP_002440425., ZP_04934218.1, NP_251152.1, YP_001345439., ZP_05639289.1, YP_276675.1, YP_257764.1, YP_575194.1, YP_672417.1, ZP_03032171.1, YP_543880.1, ZP_04004799.1, NP_752286.1, CAJ87528.1, ZP_05433039.1, NP_753124.1, CAE55681.1, ZP_04001783.1, AAZ57198.1, ZP_02814156.1, YP_308825.1, YP_002239913., YP_001174794., ZP_04622797.1, ZP_04636695.1, YP_001480624., ZP_04642182.1, ZP_03829142.1, YP_050213.1, AAN38708.1, YP_003004242., YP_003004334., ZP_05725774.1, YP_002987613., ZP_03827858.1, ZP_03833473.1, YP_003017752., ZP_05305135.1, YP_050212.1, ZP_01545092.1, YP_003158037., YP_002542793., ZP_05104655.1, ZP_01955515.1, ZP_05718784.1, ZP_03640348.1, ZP_03641287.1, ZP_03827530.1, YP_003015663., NP_930296.1, NP_929690.1, YP_002650524., YP_001489869., ZP_04504812.1, NP_603198.1, ZP_04969890.1, ZP_04569482.1, ZP_05551828.1, ZP_00144284.1, ZP_04571713.1, ZP_04859779.1, ZP_05634229.1, ZP_04860256.1, ZP_05634245.1, ZP_05634228.1, ZP_05040569.1, ZP_01305734.1, YP_261329.1, YP_349520.1, NP_287046.1, NP_309309.1, YP_002255439., YP_745484.1, ZP_03284417.1, ZP_04634430.1, YP_785245.1, YP_001894067., ZP_01751897.1, ZP_05125804.1, YP_001608846., YP_001609659., YP_002972394., YP_002972429., YP_002971801., YP_002972520., YP_001908997., YP_002650267., YP_001480300., AAM88788.1, ZP_03802954.1, ZP_04977422.1, ZP_05629136.1, YP_001708563., YP_045657.1, ZP_04662166.1, YP_001966360., YP_001480950., ZP_05705658.1, NP_637159.1, YP_243468.1, YP_363591.1, NP_642141.1, YP_363592.1, YP_001974096., NP_902541.1, NP_902542.1

**Glutamate synthase:** CAR65981.1, AAW82371.2, XP_001483729.1, EDK40360.2, XP_001382727.2, XP_001382727.2, XP_001528039.1, XP_718760.1, XP_718760.1, CAX44890.1, CAX44890.1, XP_718845.1, XP_718845.1, XP_501118.1, XP_501118.1, CAY71086.1, CAY71086.1, XP_001209476.1, XP_001209476.1, XP_001909499.1, XP_001909499.1, XP_750485.1, XP_750485.1, XP_001392067.1, XP_001392067.1, XP_001264931.1, XP_001264931.1, XP_001547433.1, XP_001547433.1, XP_001727923.1, XP_001727923.1, XP_002376254.1, XP_002376254.1, EEH03793.1, EEH03793.1, XP_957167.1, XP_957167.1, XP_367262.2, XP_367262.2, XP_381609.1, XP_381609.1, XP_002149290.1, XP_002149290.1, CAP98419.1, CAP98419.1, CAJ20840.1, CAJ20840.1, XP_662738.1, XP_662738.1, XP_001269425.1, XP_001269425.1, EED14920.1, EED14920.1, XP_001934320.1, XP_001934320.1, XP_001248715.1, XP_001248715.1, XP_001591223.1, XP_001591223.1, EEP78379.1, EEP78379.1, EEH21300.1, EEH21300.1, XP_454839.1, XP_454839.1, NP_594133.1, NP_594133.1, NP_984386.1, NP_984386.1, XP_002175175.1, XP_002175175.1, XP_759997.1, XP_759997.1, EEH45951.1, EEH45951.1, NP_010110.1, NP_010110.1, EDV08435.1, EDV08435.1, EDN60189.1, EDN60189.1, XP_001644909.1, XP_001644909.1, XP_448821.1, XP_448821.1, XP_001878616.1, XP_001878616.1, CAA61505.1, CAA61505.1, XP_001792115.1, XP_001792115.1, ZP_01852338.1, EDZ73393.1, XP_001762938.1, AAB41904.1, XP_001731018.1, XP_001731018.1, XP_002008227.1, XP_002085195.1, XP_002047025.1, BAF80064.1, XP_002067862.1, XP_001948786.1, XP_001849147.1, XP_567571.1, EEC79670.1, EEE64638.1, XP_001957626.1, XP_001984051.1, EDZ73395.1, EEB15855.1, XP_316385.4, YP_904194.1, AAV34470.1, BAA12741.1, CAO89501.1, YP_592755.1, YP_001328376.1, YP_001520615.1, YP_002282810.1, NP_386917.1, YP_002827368.1, ZP_01616878.1, YP_002590814.1, YP_341620.1, YP_267510.1, ZP_01216250.1, ZP_02181873.1, ZP_01117720.1, YP_002789142.1, YP_622576.1, YP_622576.1, YP_632097.1, YP_002552188.1, YP_002552188.1, YP_585404.1, ZP_04481621.1, ZP_03208356.1, XP_970053.1, NP_001041678.1, XP_001693082.1, CAA90032.2, NP_509693.1, XP_001228301.1, ZP_04424972.1, YP_001413236.1, YP_464029.1, YP_001964804.1, YP_001378049.1, YP_343609.1, YP_002626313.1, YP_002491287.1, YP_002133230.1, YP_001237510.1, YP_002287941.1, YP_001834082.1, YP_498445.1, ZP_04360050.1, YP_459314.1, ZP_01254365.1, YP_001208096.1, YP_001372806.1, ZP_04595566.1, NP_699261.1, YP_001621907.1, NP_541017.1, YP_222866.1, YP_001257130.1, YP_002733893.1, ZP_03786566.1, CAM77927.1, YP_001354991.1, YP_482099.1, NP_882256.1, NP_886382.1, NP_891373.1, YP_935144.1, YP_001101312.1, ZP_03899724.1, YP_283446.1, YP_715148.1, YP_001132384.1, YP_787786.1, YP_530651.1, YP_779621.1, YP_001523376.1, YP_158268.1, YP_001628755.1, YP_001506278.1, ZP_01551583.1, ZP_03268813.1, YP_488125.1, NP_946244.1, YP_001989992.1, YP_568014.1, YP_319555.1, YP_002364119.1, YP_576444.1, YP_908382.1, ZP_01046241.1, YP_001260774.1, YP_985061.1, YP_002891702.1, YP_594134.1, YP_002099477.1, YP_002006876.1, YP_001792419.1, YP_297330.1, YP_640207.1, YP_001071330.1, YP_001947370.1, ZP_03574660.1, YP_001578497.1, ZP_03586417.1, ZP_02380107.1, ZP_01863403.1, YP_001807047.1, YP_002092562.1, YP_001763683.1, YP_997159.1, YP_772215.1, ZP_02891753.1, YP_002229455.1, ZP_02907807.1, YP_367745.1, YP_834049.1, YP_001118232.1, YP_001566572.1, ZP_03545143.1, YP_524175.1, YP_969380.1, YP_547645.1, YP_980923.1, AAW82371.2, CAR65981.1, XP_001483729.1, EDK40360.2, XP_001382727.2, XP_001382727.2, XP_001528039.1, XP_718760.1, XP_718760.1, CAX44890.1, CAX44890.1, XP_718845.1, XP_718845.1, XP_501118.1, XP_501118.1, CAY71086.1, CAY71086.1, XP_001209476.1, XP_001209476.1, XP_001909499.1, XP_001909499.1, XP_750485.1, XP_750485.1, XP_001392067.1, XP_001392067.1, XP_001264931.1, XP_001264931.1, XP_002376254.1, XP_002376254.1, XP_001727923.1, XP_001727923.1, XP_001547433.1, XP_001547433.1, EEH03793.1, EEH03793.1, XP_957167.1, XP_957167.1, XP_367262.2, XP_367262.2, XP_381609.1, XP_381609.1, CAP98419.1, CAP98419.1, XP_002149290.1, XP_002149290.1, XP_001269425.1, XP_001269425.1, XP_662738.1, XP_662738.1, EED14920.1, EED14920.1, CAJ20840.1, CAJ20840.1, XP_001934320.1, XP_001934320.1, XP_001248715.1, XP_001248715.1, XP_001591223.1, XP_001591223.1, EEH21300.1, EEH21300.1, EEP78379.1, EEP78379.1, XP_454839.1, XP_454839.1, NP_984386.1, NP_984386.1, NP_594133.1, NP_594133.1, XP_002175175.1, XP_002175175.1, XP_759997.1, XP_759997.1, EEH45951.1, EEH45951.1, NP_010110.1, NP_010110.1, EDV08435.1, EDV08435.1, EDN60189.1, EDN60189.1, XP_448821.1, XP_448821.1, XP_001644909.1, XP_001644909.1, XP_001878616.1, XP_001878616.1, CAA61505.1, CAA61505.1, XP_001792115.1, XP_001792115.1, ZP_01852338.1, EDZ73393.1, XP_001762938.1, AAB41904.1, XP_001731018.1, XP_001731018.1, XP_002008227.1, XP_002085195.1, XP_002047025.1, XP_002067862.1, BAF80064.1, XP_567571.1, XP_001948786.1, XP_001849147.1, XP_001957626.1, XP_001984051.1, EEC79670.1, EDZ73395.1, EEE64638.1, EEB15855.1, YP_904194.1, XP_316385.4, AAV34470.1, BAA12741.1, CAO89501.1, YP_592755.1, YP_001328376.1, YP_001520615.1, YP_002282810.1, NP_386917.1, YP_002827368.1, YP_002590814.1, ZP_01616878.1, YP_341620.1, YP_267510.1, ZP_02181873.1, ZP_01117720.1, ZP_01216250.1, YP_002789142.1, YP_622576.1, YP_622576.1, YP_632097.1, YP_002552188.1, YP_002552188.1, YP_585404.1, ZP_04481621.1, ZP_03208356.1, XP_970053.1, NP_001041678.1, XP_001693082.1, CAA90032.2, NP_509693.1, XP_001228301.1, ZP_04424972.1, YP_001413236.1, YP_001964804.1, YP_464029.1, YP_001378049.1, YP_343609.1, YP_002626313.1, YP_001834082.1, YP_002491287.1, YP_002133230.1, YP_001237510.1, YP_002287941.1, YP_498445.1, YP_459314.1, ZP_01254365.1, ZP_04360050.1, YP_001208096.1, YP_001372806.1, YP_001621907.1, NP_699261.1, ZP_04595566.1, YP_001257130.1, YP_222866.1, NP_541017.1, ZP_03786566.1, YP_002733893.1, YP_482099.1, YP_001594030.1, CAM77927.1, NP_886382.1, NP_891373.1, NP_882256.1, YP_001354991.1, YP_283446.1, YP_935144.1, ZP_03899724.1, YP_715148.1, YP_787786.1, YP_001101312.1, YP_530651.1, YP_001132384.1, YP_779621.1, ZP_01551583.1, YP_001523376.1, YP_001628755.1, ZP_03268813.1, YP_158268.1, YP_001506278.1, YP_488125.1, YP_001989992.1, NP_946244.1, YP_002364119.1, YP_319555.1, YP_594134.1, YP_001260774.1, YP_576444.1, YP_568014.1, YP_002891702.1, YP_908382.1, ZP_01046241.1, YP_002099477.1, YP_985061.1, YP_640207.1, YP_001071330.1, ZP_03586417.1, YP_001947370.1, ZP_03574660.1, YP_001578497.1, YP_002006876.1, YP_001792419.1, YP_297330.1, ZP_02380107.1, YP_001763683.1, YP_001807047.1, YP_002092562.1, YP_772215.1, ZP_02891753.1, YP_002229455.1, ZP_01863403.1, YP_367745.1, ZP_02907807.1, YP_834049.1, YP_001118232.1, YP_997159.1, ZP_03545143.1, YP_001566572.1, YP_524175.1, YP_969380.1, YP_547645.1, YP_980923.1, AAW82371.2, CAR65981.1, XP_001483729.1, EDK40360.2, XP_001382727.2, XP_001382727.2, XP_001528039.1, XP_718760.1, XP_718760.1, CAX44890.1, CAX44890.1, XP_718845.1, XP_718845.1, XP_501118.1, XP_501118.1, CAY71086.1, CAY71086.1, XP_001209476.1, XP_001209476.1, XP_001909499.1, XP_001909499.1, XP_750485.1, XP_750485.1, XP_001392067.1, XP_001392067.1, XP_001264931.1, XP_001264931.1, XP_002376254.1, XP_002376254.1, XP_001727923.1, XP_001727923.1, XP_001547433.1, XP_001547433.1, EEH03793.1, EEH03793.1, XP_957167.1, XP_957167.1, XP_367262.2, XP_367262.2, XP_381609.1, XP_381609.1, CAP98419.1, CAP98419.1, XP_002149290.1, XP_002149290.1, XP_001269425.1, XP_001269425.1, XP_662738.1, XP_662738.1, EED14920.1, EED14920.1, CAJ20840.1, CAJ20840.1, XP_001934320.1, XP_001934320.1, XP_001248715.1, XP_001248715.1, XP_001591223.1, XP_001591223.1, EEH21300.1, EEH21300.1, EEP78379.1, EEP78379.1, XP_454839.1, XP_454839.1, NP_984386.1, NP_984386.1, NP_594133.1, NP_594133.1, XP_002175175.1, XP_002175175.1, XP_759997.1, XP_759997.1, EEH45951.1, EEH45951.1, NP_010110.1, NP_010110.1, EDV08435.1, EDV08435.1, EDN60189.1, EDN60189.1, XP_448821.1, XP_448821.1, XP_001644909.1, XP_001644909.1, XP_001878616.1, XP_001878616.1, CAA61505.1, CAA61505.1, XP_001792115.1, XP_001792115.1, ZP_01852338.1, EDZ73393.1, XP_001762938.1, AAB41904.1, XP_001731018.1, XP_001731018.1, XP_002008227.1, XP_002085195.1, XP_002047025.1, XP_002067862.1, BAF80064.1, XP_567571.1, XP_001948786.1, XP_001849147.1, XP_001957626.1, XP_001984051.1, EEC79670.1, EDZ73395.1, EEE64638.1, EEB15855.1, YP_904194.1, XP_316385.4, AAV34470.1, BAA12741.1, CAO89501.1, YP_592755.1, YP_001328376.1, YP_001520615.1, YP_002282810.1, NP_386917.1, YP_002827368.1, YP_002590814.1, ZP_01616878.1, YP_341620.1, YP_267510.1, ZP_02181873.1, ZP_01117720.1, ZP_01216250.1, YP_002789142.1, YP_622576.1, YP_622576.1, YP_632097.1, YP_002552188.1, YP_002552188.1, YP_585404.1, ZP_04481621.1, ZP_03208356.1, XP_970053.1, NP_001041678.1, XP_001693082.1, CAA90032.2, NP_509693.1, XP_001228301.1, ZP_04424972.1, YP_001413236.1, YP_001964804.1, YP_464029.1, YP_001378049.1, YP_343609.1, YP_002626313.1, YP_001834082.1, YP_002491287.1, YP_002133230.1, YP_001237510.1, YP_002287941.1, YP_498445.1, YP_459314.1, ZP_01254365.1, ZP_04360050.1, YP_001208096.1, YP_001372806.1, YP_001621907.1, NP_699261.1, ZP_04595566.1, YP_001257130.1, YP_222866.1, NP_541017.1, ZP_03786566.1, YP_002733893.1, YP_482099.1, YP_001594030.1, CAM77927.1, NP_886382.1, NP_891373.1, NP_882256.1, YP_001354991.1, YP_283446.1, YP_935144.1, ZP_03899724.1, YP_715148.1, YP_787786.1, YP_001101312.1, YP_530651.1, YP_001132384.1, YP_779621.1, ZP_01551583.1, YP_001523376.1, YP_001628755.1, ZP_03268813.1, YP_158268.1, YP_001506278.1, YP_488125.1, YP_001989992.1, NP_946244.1, YP_002364119.1, YP_319555.1, YP_594134.1, YP_001260774.1, YP_576444.1, YP_568014.1, YP_002891702.1, YP_908382.1, ZP_01046241.1, YP_002099477.1, YP_985061.1, YP_640207.1, YP_001071330.1, ZP_03586417.1, YP_001947370.1, ZP_03574660.1, YP_001578497.1, YP_002006876.1, YP_001792419.1, YP_297330.1, ZP_02380107.1, YP_001763683.1, YP_001807047.1, YP_002092562.1, YP_772215.1, ZP_02891753.1, YP_002229455.1, ZP_01863403.1, YP_367745.1, ZP_02907807.1, YP_834049.1, YP_001118232.1, YP_997159.1, ZP_03545143.1, YP_001566572.1, YP_524175.1, YP_969380.1, YP_547645.1, YP_980923.1, AAW82371.2, CAR65981.1, XP_001483729.1, EDK40360.2, XP_001382727.2, XP_001382727.2, XP_001528039.1, XP_718760.1, XP_718760.1, CAX44890.1, CAX44890.1, XP_718845.1, XP_718845.1, XP_501118.1, XP_501118.1, CAY71086.1, CAY71086.1, XP_001209476.1, XP_001209476.1, XP_001909499.1, XP_001909499.1, XP_750485.1, XP_750485.1, XP_001392067.1, XP_001392067.1, XP_001264931.1, XP_001264931.1, XP_002376254.1, XP_002376254.1, XP_001727923.1, XP_001727923.1, XP_001547433.1, XP_001547433.1, EEH03793.1, EEH03793.1, XP_957167.1, XP_957167.1, XP_367262.2, XP_367262.2, XP_381609.1, XP_381609.1, CAP98419.1, CAP98419.1, XP_002149290.1, XP_002149290.1, XP_001269425.1, XP_001269425.1, XP_662738.1, XP_662738.1, EED14920.1, EED14920.1, CAJ20840.1, CAJ20840.1, XP_001934320.1, XP_001934320.1, XP_001248715.1, XP_001248715.1, XP_001591223.1, XP_001591223.1, EEH21300.1, EEH21300.1, EEP78379.1, EEP78379.1, XP_454839.1, XP_454839.1, NP_984386.1, NP_984386.1, NP_594133.1, NP_594133.1, XP_002175175.1, XP_002175175.1, XP_759997.1, XP_759997.1, EEH45951.1, EEH45951.1, NP_010110.1, NP_010110.1, EDV08435.1, EDV08435.1, EDN60189.1, EDN60189.1, XP_448821.1, XP_448821.1, XP_001644909.1, XP_001644909.1, XP_001878616.1, XP_001878616.1, CAA61505.1, CAA61505.1, XP_001792115.1, XP_001792115.1, ZP_01852338.1, EDZ73393.1, XP_001762938.1, AAB41904.1, XP_001731018.1, XP_001731018.1, XP_002008227.1, XP_002085195.1, XP_002047025.1, XP_002067862.1, BAF80064.1, XP_567571.1, XP_001948786.1, XP_001849147.1, XP_001957626.1, XP_001984051.1, EEC79670.1, EDZ73395.1, EEE64638.1, EEB15855.1, YP_904194.1, XP_316385.4, AAV34470.1, BAA12741.1, CAO89501.1, YP_592755.1, YP_001328376.1, YP_001520615.1, YP_002282810.1, NP_386917.1, YP_002827368.1, YP_002590814.1, ZP_01616878.1, YP_341620.1, YP_267510.1, ZP_02181873.1, ZP_01117720.1, ZP_01216250.1, YP_002789142.1, YP_622576.1, YP_622576.1, YP_632097.1, YP_002552188.1, YP_002552188.1, YP_585404.1, ZP_04481621.1, ZP_03208356.1, XP_970053.1, NP_001041678.1, XP_001693082.1, CAA90032.2, NP_509693.1, XP_001228301.1, ZP_04424972.1, YP_001413236.1, YP_001964804.1, YP_464029.1, YP_001378049.1, YP_343609.1, YP_002626313.1, YP_001834082.1, YP_002491287.1, YP_002133230.1, YP_001237510.1, YP_002287941.1, YP_498445.1, YP_459314.1, ZP_01254365.1, ZP_04360050.1, YP_001208096.1, YP_001372806.1, YP_001621907.1, NP_699261.1, ZP_04595566.1, YP_001257130.1, YP_222866.1, NP_541017.1, ZP_03786566.1, YP_002733893.1, YP_482099.1, YP_001594030.1, CAM77927.1, NP_886382.1, NP_891373.1, NP_882256.1, YP_001354991.1, YP_283446.1, YP_935144.1, ZP_03899724.1, YP_715148.1, YP_787786.1, YP_001101312.1, YP_530651.1, YP_001132384.1, YP_779621.1, ZP_01551583.1, YP_001523376.1, YP_001628755.1, ZP_03268813.1, YP_158268.1, YP_001506278.1, YP_488125.1, YP_001989992.1, NP_946244.1, YP_002364119.1, YP_319555.1, YP_594134.1, YP_001260774.1, YP_576444.1, YP_568014.1, YP_002891702.1, YP_908382.1, ZP_01046241.1, YP_002099477.1, YP_985061.1, YP_640207.1, YP_001071330.1, ZP_03586417.1, YP_001947370.1, ZP_03574660.1, YP_001578497.1, YP_002006876.1, YP_001792419.1, YP_297330.1, ZP_02380107.1, YP_001763683.1, YP_001807047.1, YP_002092562.1, YP_772215.1, ZP_02891753.1, YP_002229455.1, ZP_01863403.1, YP_367745.1, ZP_02907807.1, YP_834049.1, YP_001118232.1, YP_997159.1, ZP_03545143.1, YP_001566572.1, YP_524175.1, YP_969380.1, YP_547645.1, YP_980923.1, CAR65981.1, AAW82371.2, XP_001483729.1, EDK40360.2, XP_001382727.2, XP_001382727.2, XP_001528039.1, XP_718760.1, XP_718760.1, CAX44890.1, CAX44890.1, XP_718845.1, XP_718845.1, XP_501118.1, XP_501118.1, CAY71086.1, CAY71086.1, XP_001209476.1, XP_001209476.1, XP_001909499.1, XP_001909499.1, XP_750485.1, XP_750485.1, XP_001392067.1, XP_001392067.1, XP_001264931.1, XP_001264931.1, XP_001547433.1, XP_001547433.1, XP_001727923.1, XP_001727923.1, XP_002376254.1, XP_002376254.1, EEH03793.1, EEH03793.1, XP_957167.1, XP_957167.1, XP_367262.2, XP_367262.2, XP_381609.1, XP_381609.1, XP_002149290.1, XP_002149290.1, CAP98419.1, CAP98419.1, CAJ20840.1, CAJ20840.1, XP_662738.1, XP_662738.1, XP_001269425.1, XP_001269425.1, EED14920.1, EED14920.1, XP_001934320.1, XP_001934320.1, XP_001248715.1, XP_001248715.1, XP_001591223.1, XP_001591223.1, EEP78379.1, EEP78379.1, EEH21300.1, EEH21300.1, XP_454839.1, XP_454839.1, NP_594133.1, NP_594133.1, NP_984386.1, NP_984386.1, XP_002175175.1, XP_002175175.1, XP_759997.1, XP_759997.1, EEH45951.1, EEH45951.1, NP_010110.1, NP_010110.1, EDV08435.1, EDV08435.1, EDN60189.1, EDN60189.1, XP_001644909.1, XP_001644909.1, XP_448821.1, XP_448821.1, XP_001878616.1, XP_001878616.1, CAA61505.1, CAA61505.1, XP_001792115.1, XP_001792115.1, ZP_01852338.1, EDZ73393.1, XP_001762938.1, AAB41904.1, XP_001731018.1, XP_001731018.1, XP_002008227.1, XP_002085195.1, XP_002047025.1, BAF80064.1, XP_002067862.1, XP_001948786.1, XP_001849147.1, XP_567571.1, EEC79670.1, EEE64638.1, XP_001957626.1, XP_001984051.1, EDZ73395.1, EEB15855.1, XP_316385.4, YP_904194.1, AAV34470.1, BAA12741.1, CAO89501.1, YP_592755.1, YP_001328376.1, YP_001520615.1, YP_002282810.1, NP_386917.1, YP_002827368.1, ZP_01616878.1, YP_002590814.1, YP_341620.1, YP_267510.1, ZP_01216250.1, ZP_02181873.1, ZP_01117720.1, YP_002789142.1, YP_622576.1, YP_622576.1, YP_632097.1, YP_002552188.1, YP_002552188.1, YP_585404.1, ZP_04481621.1, ZP_03208356.1, XP_970053.1, NP_001041678.1, XP_001693082.1, CAA90032.2, NP_509693.1, XP_001228301.1, ZP_04424972.1, YP_001413236.1, YP_464029.1, YP_001964804.1, YP_001378049.1, YP_343609.1, YP_002626313.1, YP_002491287.1, YP_002133230.1, YP_001237510.1, YP_002287941.1, YP_001834082.1, YP_498445.1, ZP_04360050.1, YP_459314.1, ZP_01254365.1, YP_001208096.1, YP_001372806.1, ZP_04595566.1, NP_699261.1, YP_001621907.1, NP_541017.1, YP_222866.1, YP_001257130.1, YP_002733893.1, ZP_03786566.1, CAM77927.1, YP_001354991.1, YP_482099.1, NP_882256.1, NP_886382.1, NP_891373.1, YP_935144.1, YP_001101312.1, ZP_03899724.1, YP_283446.1, YP_715148.1, YP_001132384.1, YP_787786.1, YP_530651.1, YP_779621.1, YP_001523376.1, YP_158268.1, YP_001628755.1, YP_001506278.1, ZP_01551583.1, ZP_03268813.1, YP_488125.1, NP_946244.1, YP_001989992.1, YP_568014.1, YP_319555.1, YP_002364119.1, YP_576444.1, YP_908382.1, ZP_01046241.1, YP_001260774.1, YP_985061.1, YP_002891702.1, YP_594134.1, YP_002099477.1, YP_002006876.1, YP_001792419.1, YP_297330.1, YP_640207.1, YP_001071330.1, YP_001947370.1, ZP_03574660.1, YP_001578497.1, ZP_03586417.1, ZP_02380107.1, ZP_01863403.1, YP_001807047.1, YP_002092562.1, YP_001763683.1, YP_997159.1, YP_772215.1, ZP_02891753.1, YP_002229455.1, ZP_02907807.1, YP_367745.1, YP_834049.1, YP_001118232.1, YP_001566572.1, ZP_03545143.1, YP_524175.1, YP_969380.1, YP_547645.1, YP_980923.1, CAR65981.1, AAW82371.2, XP_001483729.1, EDK40360.2, XP_001382727.2, XP_001382727.2, XP_001528039.1, XP_718760.1, XP_718760.1, CAX44890.1, CAX44890.1, XP_718845.1, XP_718845.1, XP_501118.1, XP_501118.1, CAY71086.1, CAY71086.1, XP_001209476.1, XP_001209476.1, XP_001909499.1, XP_001909499.1, XP_750485.1, XP_750485.1, XP_001392067.1, XP_001392067.1, XP_001264931.1, XP_001264931.1, XP_001547433.1, XP_001547433.1, XP_001727923.1, XP_001727923.1, XP_002376254.1, XP_002376254.1, EEH03793.1, EEH03793.1, XP_957167.1, XP_957167.1, XP_367262.2, XP_367262.2, XP_381609.1, XP_381609.1, XP_002149290.1, XP_002149290.1, CAP98419.1, CAP98419.1, CAJ20840.1, CAJ20840.1, XP_662738.1, XP_662738.1, XP_001269425.1, XP_001269425.1, EED14920.1, EED14920.1, XP_001934320.1, XP_001934320.1, XP_001248715.1, XP_001248715.1, XP_001591223.1, XP_001591223.1, EEP78379.1, EEP78379.1, EEH21300.1, EEH21300.1, XP_454839.1, XP_454839.1, NP_594133.1, NP_594133.1, NP_984386.1, NP_984386.1, XP_002175175.1, XP_002175175.1, XP_759997.1, XP_759997.1, EEH45951.1, EEH45951.1, NP_010110.1, NP_010110.1, EDV08435.1, EDV08435.1, EDN60189.1, EDN60189.1, XP_001644909.1, XP_001644909.1, XP_448821.1, XP_448821.1, XP_001878616.1, XP_001878616.1, CAA61505.1, CAA61505.1, XP_001792115.1, XP_001792115.1, ZP_01852338.1, EDZ73393.1, XP_001762938.1, AAB41904.1, XP_001731018.1, XP_001731018.1, XP_002008227.1, XP_002085195.1, XP_002047025.1, BAF80064.1, XP_002067862.1, XP_001948786.1, XP_001849147.1, XP_567571.1, EEC79670.1, EEE64638.1, XP_001957626.1, XP_001984051.1, EDZ73395.1, EEB15855.1, XP_316385.4, YP_904194.1, AAV34470.1, BAA12741.1, CAO89501.1, YP_592755.1, YP_001328376.1, YP_001520615.1, YP_002282810.1, NP_386917.1, YP_002827368.1, ZP_01616878.1, YP_002590814.1, YP_341620.1, YP_267510.1, ZP_01216250.1, ZP_02181873.1, ZP_01117720.1, YP_002789142.1, YP_622576.1, YP_622576.1, YP_632097.1, YP_002552188.1, YP_002552188.1, YP_585404.1, ZP_04481621.1, ZP_03208356.1, XP_970053.1, NP_001041678.1, XP_001693082.1, CAA90032.2, NP_509693.1, XP_001228301.1, ZP_04424972.1, YP_001413236.1, YP_464029.1, YP_001964804.1, YP_001378049.1, YP_343609.1, YP_002626313.1, YP_002491287.1, YP_002133230.1, YP_001237510.1, YP_002287941.1, YP_001834082.1, YP_498445.1, ZP_04360050.1, YP_459314.1, ZP_01254365.1, YP_001208096.1, YP_001372806.1, ZP_04595566.1, NP_699261.1, YP_001621907.1, NP_541017.1, YP_222866.1, YP_001257130.1, YP_002733893.1, ZP_03786566.1, CAM77927.1, YP_001354991.1, YP_482099.1, NP_882256.1, NP_886382.1, NP_891373.1, YP_935144.1, YP_001101312.1, ZP_03899724.1, YP_283446.1, YP_715148.1, YP_001132384.1, YP_787786.1, YP_530651.1, YP_779621.1, YP_001523376.1, YP_158268.1, YP_001628755.1, YP_001506278.1, ZP_01551583.1, ZP_03268813.1, YP_488125.1, NP_946244.1, YP_001989992.1, YP_568014.1, YP_319555.1, YP_002364119.1, YP_576444.1, YP_908382.1, ZP_01046241.1, YP_001260774.1, YP_985061.1, YP_002891702.1, YP_594134.1, YP_002099477.1, YP_002006876.1, YP_001792419.1, YP_297330.1, YP_640207.1, YP_001071330.1, YP_001947370.1, ZP_03574660.1, YP_001578497.1, ZP_03586417.1, ZP_02380107.1, ZP_01863403.1, YP_001807047.1, YP_002092562.1, YP_001763683.1, YP_997159.1, YP_772215.1, ZP_02891753.1, YP_002229455.1, ZP_02907807.1, YP_367745.1, YP_834049.1, YP_001118232.1, YP_001566572.1, ZP_03545143.1, YP_524175.1, YP_969380.1, YP_547645.1, YP_980923.1, XP_001792115.1, XP_001934320.1, EED14920.1, XP_662738.1, XP_001392067.1, XP_001264931.1, XP_750485.1, XP_002149290.1, EEH21300.1, XP_001591223.1, XP_001727923.1, XP_001269425.1, XP_957167.1, CAP98419.1, EEP78379.1, XP_001248715.1, XP_367262.2, XP_001909499.1, XP_381609.1, XP_002376254.1, EEH45951.1, CAJ20840.1, XP_001209476.1, XP_001547433.1, EEH03793.1, XP_001228301.1, EDK40360.2, EDK40360.2, XP_001483729.1, XP_001483729.1, CAR65981.1, CAR65981.1, AAW82371.2, AAW82371.2, XP_501118.1, XP_001878616.1, XP_001382727.2, XP_002175175.1, NP_594133.1, CAX44890.1, XP_567571.1, XP_718760.1, XP_718845.1, XP_001731018.1, XP_759997.1, CAY71086.1, XP_454839.1, XP_448821.1, NP_984386.1, XP_002267865.1, Q03460.1, AAB41904.1, XP_001644909.1, NP_010110.1, CAA61505.1, EDV08435.1, EDN60189.1, EDZ73395.1, XP_001649585.1, AAV31916.2, XP_316385.4, XP_002095083.1, XP_001984051.1, XP_002008227.1, NP_648922.1, XP_002067862.1, XP_001973010.1, XP_001352442.1, NP_730201.1, XP_001957626.1, XP_970053.1, XP_001849147.1, XP_002047025.1, XP_001605708.1, XP_001948786.1, XP_396817.2, EEB15855.1, XP_002247328.1, XP_002237632.1, NP_001041678.1, XP_001630774.1, XP_001693082.1, CAA90032.2, NP_509693.1, XP_001899642.1, XP_002160465.1, AAO24979.1, XP_002026159.1, XP_001675805.1, YP_289235.1, NP_959107.1, YP_908381.1, YP_001853674.1, YP_980000.1, NP_218375.1, ZP_03422455.1, NP_857525.1, NP_301171.1, YP_001625472.1, EDK40360.2, XP_001483729.1, CAR65981.1, AAW82371.2, XP_001382727.2, XP_001382727.2, XP_001528039.1, XP_718760.1, XP_718760.1, CAX44890.1, CAX44890.1, XP_718845.1, XP_718845.1, XP_501118.1, XP_501118.1, CAY71086.1, CAY71086.1, XP_001209476.1, XP_001209476.1, XP_750485.1, XP_750485.1, XP_001264931.1, XP_001264931.1, XP_002376254.1, XP_002376254.1, XP_001727923.1, XP_001727923.1, XP_001269425.1, XP_001269425.1, EEH03793.1, EEH03793.1, XP_001547433.1, XP_001547433.1, XP_001909499.1, XP_001909499.1, XP_001392067.1, XP_001392067.1, XP_367262.2, XP_367262.2, XP_381609.1, XP_381609.1, XP_957167.1, XP_957167.1, XP_662738.1, XP_662738.1, CAJ20840.1, CAJ20840.1, XP_002149290.1, XP_002149290.1, EED14920.1, EED14920.1, CAP98419.1, CAP98419.1, XP_001248715.1, XP_001248715.1, XP_001934320.1, XP_001934320.1, XP_001591223.1, XP_001591223.1, EEH21300.1, EEH21300.1, EEP78379.1, EEP78379.1, EEH45951.1, EEH45951.1, XP_454839.1, XP_454839.1, NP_984386.1, NP_984386.1, XP_001544350.1, XP_759997.1, XP_759997.1, XP_002175175.1, XP_002175175.1, NP_594133.1, NP_594133.1, XP_001878616.1, XP_001878616.1, NP_010110.1, NP_010110.1, EDV08435.1, EDV08435.1, EDN60189.1, EDN60189.1, XP_448821.1, XP_448821.1, XP_001644909.1, XP_001644909.1, CAA61505.1, CAA61505.1, XP_001792115.1, XP_001792115.1, ZP_01852338.1, XP_001762938.1, XP_567571.1, XP_001731018.1, XP_001731018.1, XP_001605708.1, XP_001228301.1, XP_001228301.1, EDZ73395.1, YP_002282810.1, YP_471049.1, YP_001979936.1, ZP_01451630.1, ZP_01451630.1, YP_530651.1, XP_002184279.1, YP_680351.1, YP_002015315.1, NP_293907.1, NP_293907.1, YP_001900797.1, YP_001900797.1, YP_947593.1, ZP_04481621.1, ZP_04481621.1, ZP_03208356.1, NP_001041678.1, XP_001693082.1, YP_114471.1, NP_796863.1, ZP_01868601.1, NP_759543.1, ZP_02008389.1, NP_933434.1, ZP_04360050.1, ZP_04386036.1, YP_002666920.1, YP_703675.1, ZP_01065693.1, NP_521086.1, YP_001106194.1, ZP_00991996.1, YP_002416131.1, YP_002763498.1, YP_205507.1, YP_002780727.1, ZP_01261464.1, YP_128767.1, YP_498445.1, ZP_01978559.1, ZP_04397067.1, ZP_04418026.1, ZP_01254365.1, ZP_03876620.1, YP_002175850.1, ZP_01956620.1, NP_232003.1, ZP_04410627.1, ZP_04403469.1, ZP_04413769.1, ZP_01981478.1, YP_002156924.1, ZP_01950323.1, ZP_01221149.1, YP_001354991.1, YP_002252282.1, ZP_00945229.1, YP_002263909.1, YP_002723787.1, YP_001101312.1, ZP_01815688.1, YP_001154880.1, YP_001628755.1, NP_882256.1, YP_482099.1, NP_891373.1, YP_864633.1, NP_886382.1, YP_787786.1, ZP_01236408.1, ZP_04024767.1, YP_001859006.1, YP_935144.1, YP_715148.1, ZP_02886874.1, ZP_03268813.1, YP_560620.1, YP_001897205.1, YP_283446.1, YP_297330.1, YP_002006876.1, YP_002663735.1, YP_001260774.1, YP_002898540.1, YP_001067977.1, YP_001060694.1, ZP_01769810.1, YP_109752.1, YP_104256.1, YP_001506278.1, YP_335082.1, ZP_01226794.1, YP_002354507.1, ZP_02357275.1, ZP_02380107.1, YP_727867.1, YP_924211.1, ZP_01718601.1, YP_367745.1, YP_585404.1, YP_002891702.1, YP_158268.1, YP_002552188.1, YP_622576.1, YP_985061.1, NP_626286.1, ZP_04366654.1, NP_827365.1, YP_002487762.1, ZP_01863403.1, YP_942445.1, YP_831185.1, YP_002207918.1, YP_997159.1, ZP_01041058.1, YP_001566572.1, YP_002184527.1, ZP_03545143.1, YP_547645.1, ZP_04598864.1, YP_001022291.1, YP_980923.1, YP_524175.1, ZP_03855351.1, YP_969380.1, XP_001483729.1, EDK40360.2, CAR65981.1, AAW82371.2, XP_001382727.2, XP_001382727.2, XP_718760.1, XP_718760.1, XP_001528039.1, CAX44890.1, CAX44890.1, XP_718845.1, XP_718845.1, XP_501118.1, XP_501118.1, CAY71086.1, CAY71086.1, XP_001209476.1, XP_001209476.1, XP_001264931.1, XP_001264931.1, XP_750485.1, XP_750485.1, XP_002376254.1, XP_002376254.1, XP_001727923.1, XP_001727923.1, EEH03793.1, EEH03793.1, XP_001269425.1, XP_001269425.1, XP_001547433.1, XP_001547433.1, XP_001909499.1, XP_001909499.1, XP_367262.2, XP_367262.2, XP_001392067.1, XP_001392067.1, XP_957167.1, XP_957167.1, XP_381609.1, XP_381609.1, XP_662738.1, XP_662738.1, CAJ20840.1, CAJ20840.1, XP_002149290.1, XP_002149290.1, CAP98419.1, CAP98419.1, EED14920.1, EED14920.1, XP_001248715.1, XP_001248715.1, XP_001934320.1, XP_001934320.1, XP_001591223.1, XP_001591223.1, EEH21300.1, EEH21300.1, EEP78379.1, EEP78379.1, EEH45951.1, EEH45951.1, XP_454839.1, XP_454839.1, NP_984386.1, NP_984386.1, XP_001544350.1, XP_759997.1, XP_759997.1, XP_002175175.1, XP_002175175.1, NP_594133.1, NP_594133.1, XP_001878616.1, XP_001878616.1, NP_010110.1, NP_010110.1, EDV08435.1, EDV08435.1, EDN60189.1, EDN60189.1, XP_448821.1, XP_448821.1, XP_001644909.1, XP_001644909.1, CAA61505.1, CAA61505.1, XP_001792115.1, XP_001792115.1, ZP_01852338.1, ZP_01852338.1, XP_001762938.1, XP_567571.1, XP_001731018.1, XP_001731018.1, XP_001605708.1, XP_001228301.1, XP_001228301.1, EDZ73395.1, YP_002282810.1, YP_471049.1, YP_001979936.1, ZP_01451630.1, ZP_01451630.1, YP_530651.1, YP_680351.1, XP_002184279.1, YP_002015315.1, NP_293907.1, NP_293907.1, YP_001900797.1, YP_001900797.1, YP_947593.1, YP_947593.1, ZP_04481621.1, ZP_03208356.1, XP_001948786.1, NP_001041678.1, XP_001693082.1, ZP_01991096.1, NP_796863.1, ZP_01868601.1, NP_759543.1, NP_933434.1, ZP_02008389.1, YP_002666920.1, ZP_04360050.1, ZP_01065693.1, ZP_00991996.1, YP_002416131.1, YP_001444154.1, ZP_01987146.1, YP_205507.1, YP_128767.1, ZP_04386036.1, ZP_01261464.1, ZP_01978559.1, YP_002175850.1, ZP_04418026.1, ZP_01956620.1, ZP_04397067.1, ZP_04410627.1, NP_232003.1, YP_703675.1, ZP_01981478.1, ZP_04403469.1, ZP_04413769.1, YP_002156924.1, YP_001106194.1, ZP_01950323.1, ZP_03876620.1, NP_521086.1, ZP_01221149.1, ZP_01254365.1, YP_002263909.1, YP_498445.1, YP_002780727.1, YP_002763498.1, YP_002723787.1, YP_001354991.1, ZP_00945229.1, YP_002252282.1, YP_001101312.1, ZP_01236408.1, NP_882256.1, YP_001628755.1, NP_891373.1, YP_482099.1, NP_886382.1, YP_787786.1, ZP_02886874.1, YP_935144.1, ZP_04024767.1, YP_560620.1, YP_715148.1, YP_001897205.1, YP_297330.1, YP_002006876.1, YP_283446.1, YP_001260774.1, YP_001506278.1, YP_002898540.1, YP_104256.1, YP_001067977.1, YP_001060694.1, ZP_01769810.1, YP_109752.1, YP_335082.1, YP_002099477.1, YP_443518.1, ZP_01718601.1, YP_727867.1, YP_002354507.1, ZP_02357275.1, ZP_03586417.1, ZP_02380107.1, YP_001947370.1, ZP_03574660.1, YP_585404.1, YP_367745.1, YP_002891702.1, YP_158268.1, YP_002552188.1, YP_622576.1, YP_002487762.1, YP_985061.1, YP_831185.1, YP_942445.1, YP_594134.1, YP_997159.1, YP_001566572.1, YP_002184527.1, ZP_04598864.1, ZP_03545143.1, YP_547645.1, YP_980923.1, ZP_03855351.1, YP_524175.1, YP_969380.1, XP_001792115.1, XP_001934320.1, EED14920.1, XP_662738.1, XP_001392067.1, XP_001264931.1, XP_750485.1, XP_002149290.1, EEH21300.1, XP_001591223.1, XP_001727923.1, XP_001269425.1, XP_957167.1, CAP98419.1, EEP78379.1, XP_001248715.1, XP_367262.2, XP_001909499.1, XP_381609.1, XP_002376254.1, EEH45951.1, CAJ20840.1, XP_001209476.1, XP_001547433.1, EEH03793.1, XP_001228301.1, EDK40360.2, EDK40360.2, XP_001483729.1, XP_001483729.1, CAR65981.1, CAR65981.1, AAW82371.2, AAW82371.2, XP_501118.1, XP_001878616.1, XP_001382727.2, XP_002175175.1, NP_594133.1, CAX44890.1, XP_567571.1, XP_718760.1, XP_718845.1, XP_001731018.1, XP_759997.1, CAY71086.1, XP_454839.1, XP_448821.1, NP_984386.1, XP_002267865.1, Q03460.1, AAB41904.1, XP_001644909.1, NP_010110.1, CAA61505.1, EDV08435.1, EDN60189.1, EDZ73395.1, XP_001649585.1, AAV31916.2, XP_316385.4, XP_002095083.1, XP_001984051.1, XP_002008227.1, NP_648922.1, XP_002067862.1, XP_001973010.1, XP_001352442.1, NP_730201.1, XP_001957626.1, XP_970053.1, XP_001849147.1, XP_002047025.1, XP_001605708.1, XP_001948786.1, XP_396817.2, EEB15855.1, XP_002247328.1, XP_002237632.1, NP_001041678.1, XP_001630774.1, XP_001693082.1, CAA90032.2, NP_509693.1, XP_001899642.1, XP_002160465.1, AAO24979.1, XP_002026159.1, XP_001675805.1, YP_289235.1, NP_959107.1, YP_908381.1, YP_001853674.1, YP_980000.1, NP_218375.1, ZP_03422455.1, NP_857525.1, NP_301171.1, YP_001625472.1, CAR65981.1, AAW82371.2, XP_001483729.1, EDK40360.2, XP_001382727.2, XP_001382727.2, XP_001528039.1, XP_718760.1, XP_718760.1, CAX44890.1, CAX44890.1, XP_718845.1, XP_718845.1, XP_501118.1, XP_501118.1, CAY71086.1, CAY71086.1, XP_001209476.1, XP_001209476.1, XP_001909499.1, XP_001909499.1, XP_750485.1, XP_750485.1, XP_001392067.1, XP_001392067.1, XP_001264931.1, XP_001264931.1, XP_001547433.1, XP_001547433.1, XP_001727923.1, XP_001727923.1, XP_002376254.1, XP_002376254.1, EEH03793.1, EEH03793.1, XP_957167.1, XP_957167.1, XP_367262.2, XP_367262.2, XP_381609.1, XP_381609.1, XP_002149290.1, XP_002149290.1, CAP98419.1, CAP98419.1, CAJ20840.1, CAJ20840.1, XP_662738.1, XP_662738.1, XP_001269425.1, XP_001269425.1, EED14920.1, EED14920.1, XP_001934320.1, XP_001934320.1, XP_001248715.1, XP_001248715.1, XP_001591223.1, XP_001591223.1, EEP78379.1, EEP78379.1, EEH21300.1, EEH21300.1, XP_454839.1, XP_454839.1, NP_594133.1, NP_594133.1, NP_984386.1, NP_984386.1, XP_002175175.1, XP_002175175.1, XP_759997.1, XP_759997.1, EEH45951.1, EEH45951.1, NP_010110.1, NP_010110.1, EDV08435.1, EDV08435.1, EDN60189.1, EDN60189.1, XP_001644909.1, XP_001644909.1, XP_448821.1, XP_448821.1, XP_001878616.1, XP_001878616.1, CAA61505.1, CAA61505.1, XP_001792115.1, XP_001792115.1, ZP_01852338.1, EDZ73393.1, XP_001762938.1, AAB41904.1, XP_001731018.1, XP_001731018.1, XP_002008227.1, XP_002085195.1, XP_002047025.1, BAF80064.1, XP_002067862.1, XP_001948786.1, XP_001849147.1, XP_567571.1, EEC79670.1, EEE64638.1, XP_001957626.1, XP_001984051.1, EDZ73395.1, EEB15855.1, XP_316385.4, YP_904194.1, AAV34470.1, BAA12741.1, CAO89501.1, YP_592755.1, YP_001328376.1, YP_001520615.1, YP_002282810.1, NP_386917.1, YP_002827368.1, ZP_01616878.1, YP_002590814.1, YP_341620.1, YP_267510.1, ZP_01216250.1, ZP_02181873.1, ZP_01117720.1, YP_002789142.1, YP_622576.1, YP_622576.1, YP_632097.1, YP_002552188.1, YP_002552188.1, YP_585404.1, ZP_04481621.1, ZP_03208356.1, XP_970053.1, NP_001041678.1, XP_001693082.1, CAA90032.2, NP_509693.1, XP_001228301.1, ZP_04424972.1, YP_001413236.1, YP_464029.1, YP_001964804.1, YP_001378049.1, YP_343609.1, YP_002626313.1, YP_002491287.1, YP_002133230.1, YP_001237510.1, YP_002287941.1, YP_001834082.1, YP_498445.1, ZP_04360050.1, YP_459314.1, ZP_01254365.1, YP_001208096.1, YP_001372806.1, ZP_04595566.1, NP_699261.1, YP_001621907.1, NP_541017.1, YP_222866.1, YP_001257130.1, YP_002733893.1, ZP_03786566.1, CAM77927.1, YP_001354991.1, YP_482099.1, NP_882256.1, NP_886382.1, NP_891373.1, YP_935144.1, YP_001101312.1, ZP_03899724.1, YP_283446.1, YP_715148.1, YP_001132384.1, YP_787786.1, YP_530651.1, YP_779621.1, YP_001523376.1, YP_158268.1, YP_001628755.1, YP_001506278.1, ZP_01551583.1, ZP_03268813.1, YP_488125.1, NP_946244.1, YP_001989992.1, YP_568014.1, YP_319555.1, YP_002364119.1, YP_576444.1, YP_908382.1, ZP_01046241.1, YP_001260774.1, YP_985061.1, YP_002891702.1, YP_594134.1, YP_002099477.1, YP_002006876.1, YP_001792419.1, YP_297330.1, YP_640207.1, YP_001071330.1, YP_001947370.1, ZP_03574660.1, YP_001578497.1, ZP_03586417.1, ZP_02380107.1, ZP_01863403.1, YP_001807047.1, YP_002092562.1, YP_001763683.1, YP_997159.1, YP_772215.1, ZP_02891753.1, YP_002229455.1, ZP_02907807.1, YP_367745.1, YP_834049.1, YP_001118232.1, YP_001566572.1, ZP_03545143.1, YP_524175.1, YP_969380.1, YP_547645.1, YP_980923.1

**Hypothetical protein UPF0027** YP_716116.1, YP_843194.1, YP_903236.1, ZP_01667568.1, YP_001030732.1, ZP_01723615.1, YP_001098092.1, YP_001105855.1, YP_001126344.1, NP_126316.1, YP_001161345.1, NP_143395.1, YP_001231296.1, YP_001273702.1, YP_001323619.1, YP_001324612.1, YP_001325507.1, YP_001330311.1, YP_001405106.1, ZP_02038565.1, ZP_02039578.1, YP_001421765.1, NP_242391.1, NP_247006.1, NP_276422.1, YP_001505354.1, YP_001529054.1, YP_001539783.1, YP_001548901.1, ZP_02081302.1, ZP_02084413.1, YP_001614170.1, ZP_02193861.1, YP_001665107.1, YP_001663176.1, ZP_02433182.1, ZP_02444784.1, YP_001695929.1, ZP_02911957.1, YP_001952166.1, YP_001957758.1, NP_579280.1, ZP_03026273.1, ZP_03042160.1, ZP_03147481.1, YP_002138143.1, NP_615603.1, NP_613709.1, YP_002248067.1, YP_002250998.1, NP_622931.1, YP_002306803.1, NP_633829.1, ZP_03312000.1, YP_002353160.1, ZP_03466155.1, ZP_03473199.1, ZP_03493829.1, YP_002432621.1, YP_002466035.1, YP_002478620.1, ZP_03547089.1, YP_002538764.1, ZP_03607935.1, YP_002573216.1, YP_002580296.1, YP_002582861.1, YP_002595781.1, YP_002605477.1, ZP_03649861.1, ZP_03706183.1, ZP_03724312.1, ZP_03731419.1, ZP_03736127.1, ZP_03757488.1, ZP_03780478.1, YP_002729556.1, YP_002771858.1, ZP_03816703.1, ZP_04052668.1, ZP_04342972.1, ZP_04423082.1, ZP_04606566.1, NP_954307.1, NP_987215.1, YP_075653.1, AAU82292.1, YP_163219.1, YP_183300.1, YP_183745.1, YP_290934.1, YP_305091.1, YP_386161.1, YP_389987.1, YP_429132.1, YP_448533.1, CAJ57163.1, CAJ57168.1, YP_482824.1, YP_502593.1, YP_566064.1, YP_595081.1

**Lon, ATP-dependent protease LA:** YP_625995.1, YP_630246.1, YP_644688.1, YP_673732.1, YP_656948.1, YP_696086.1, YP_698702.1, YP_754323.1, ZP_01440144.1, NP_069200.1, YP_773799.1, ZP_01462154.1, YP_843028.1, YP_878005.1, YP_878861.1, YP_902012.1, YP_942883.1, ZP_01665979.1, ZP_01666811.1, YP_001030214.1, YP_001039134.1, YP_001046192.1, YP_001087040.1, YP_001096934.1, YP_001119691.1, YP_001112718.1, YP_001113895.1, YP_001114627.1, NP_110674.1, NP_111013.1, NP_127256.1, NP_142432.1, ZP_01804987.1, YP_001184237.1, YP_001211356.1, YP_001212435.1, YP_001213436.1, YP_685198.1, YP_001234057.1, YP_001229476.1, YP_001255719.1, YP_001274142.1, ZP_01860248.1, YP_001308465.1, YP_001305576.1, YP_001318942.1, YP_001323021.1, YP_001325538.1, YP_001329653.1, YP_001385553.1, YP_001392580.1, YP_001405548.1, YP_001409929.1, YP_001411154.1, ZP_02025175.1, ZP_02042766.1, YP_001422118.1, NP_243917.1, NP_229665.1, NP_248420.1, NP_275926.1, NP_276029.1, YP_001471105.1, YP_001487685.1, NP_279404.1, YP_001512451.1, YP_001513264.1, YP_001513704.1, NP_349245.1, YP_001549532.1, NP_390699.1, NP_393576.1, NP_394540.1, ZP_02074627.1, YP_001567289.1, ZP_02193144.1, ZP_02210737.1, ZP_02330640.1, YP_001679098.1, YP_001679603.1, ZP_02477935.1, ZP_02618210.1, ZP_02620782.1, ZP_02621935.1, ZP_02633913.1, ZP_02637204.1, ZP_02640952.1, ZP_02643447.1, YP_001688492.1, ZP_02620753.2, ZP_02863365.1, ZP_02864442.1, YP_001706637.1, YP_001712986.1, YP_001717615.1, YP_001717678.1, YP_001738984.1, ZP_02883560.1, ZP_02892968.1, YP_001760835.1, YP_001765227.1, YP_001782868.1, YP_001788549.1, ZP_02909799.1, YP_001808538.1, ZP_02948139.1, ZP_02948925.1, ZP_02954839.1, NP_562307.1, NP_563551.1, YP_001847295.1, ZP_02993325.1, ZP_02993868.1, YP_001899435.1, YP_001887715.1, YP_001917275.1, YP_001918463.1, YP_001922698.1, NP_578196.1, ZP_03055920.1, NP_618179.1, NP_613901.1, YP_002231121.1, YP_002251270.1, NP_623361.1, ZP_03270164.1, ZP_03290537.1, YP_002306913.1, NP_632152.1, YP_002314967.1, YP_002314968.1, YP_002353448.1, ZP_03494885.1, YP_002461360.1, YP_002467681.1, YP_002505160.1, YP_002505170.1, YP_002509236.1, YP_002510071.1, ZP_03607395.1, YP_002565705.1, ZP_03690280.1, ZP_03693120.1, ZP_03698089.1, ZP_03729194.1, ZP_03734617.1, YP_002728228.1, YP_002730943.1, YP_002771277.1, ZP_03799886.1, ZP_03869688.1, ZP_03872876.1, ZP_03877704.1, ZP_03997959.1, ZP_04153133.1, ZP_04353226.1, ZP_04355032.1, ZP_04432710.1, ZP_04576073.1, ZP_04658422.1, ZP_04660466.1, YP_002940260.1, YP_002950547.1, YP_002958834.1, ZP_04753418.1, ZP_04790684.1, ZP_04795410.1, ZP_04803981.1, YP_002993506.1, NP_718945.1, ZP_04821499.1, YP_003003931.1, YP_003013153.1, ZP_04853010.1, ZP_04861801.1, YP_003051231.1, ZP_04873811.1, ZP_04874089.1, ZP_04874601.1, ZP_04874661.1, ZP_04877742.1, ZP_04878759.1, ZP_04945476.1, ZP_05098469.1, ZP_05120066.1, ZP_05132776.1, ZP_05304006.1, ZP_05370948.1, EET90272.1, ZP_05394530.1, ZP_05399993.1, ZP_05412656.1, ZP_05412900.1, NP_780828.1, NP_782909.1, NP_963637.1, NP_988306.1, YP_023396.1, YP_024013.1, YP_074189.1, YP_074190.1, YP_077141.1, YP_080095.1, AAU82630.1, AAU82806.1, YP_132037.1, YP_136493.1, YP_176131.1, YP_183677.1, YP_190537.1, YP_266300.1, 1Z0B, 1Z0C, 1Z0E, 1Z0W, YP_306981.1, YP_326115.1, YP_356440.1, YP_359192.1, CAI64089.1, YP_413021.1, YP_429403.1, ZP_00960717.1, YP_448248.1, YP_502445.1, ZP_01125866.1, ZP_01135216.1, ZP_01171809.1, YP_519429.1, YP_521263.1, ZP_01219417.1, ZP_01263963.1, YP_565579.1, YP_564103.1

**MupF:** ZP_01453532.1, YP_845998.1, YP_950452.1, ZP_01677290.1, YP_001084186.1, YP_001111204.1, YP_001119150.1, ZP_01787479.1, ZP_01788575.1, ZP_01788576.1, ZP_01789644.1, ZP_01791623.1, ZP_01797017.1, YP_001216625.1, YP_001291640.1, ZP_01821443.1, ZP_01822964.1, ZP_01829111.1, ZP_01878868.1, YP_001366295.1, YP_001405869.1, NP_274128.1, YP_001490593.1, YP_001469156.1, NP_312998.1, NP_298863.1, YP_001554566.1, YP_001599165.1, NP_439559.1, NP_439652.1, YP_001629581.1, YP_001633027.1, YP_001609606.1, NP_456028.1, ZP_02408903.1, ZP_02468356.1, ZP_02478064.1, ZP_02479349.1, ZP_02484617.1, YP_001714330.1, YP_001784006.1, YP_001775870.1, YP_001791278.1, ZP_03000282.1, ZP_03001036.1, ZP_03030046.1, ZP_03047230.1, ABO11584.2, ZP_03062582.1, YP_002221417.1, YP_002293862.1, YP_002297343.1, ZP_03311215.1, ZP_03339096.1, YP_002318768.1, ZP_03347779.1, ZP_03354119.1, ZP_03363557.1, ZP_03408686.1, YP_002332342.1, YP_002332452.1, YP_002330159.1, ZP_03503895.1, YP_002407927.1, YP_002342683.1, YP_002343133.1, YP_002432712.1, YP_002435155.1, YP_002439178.1, YP_002512519.1, YP_002550176.1, ZP_03698223.1, ZP_03714699.1, ZP_03747324.1, ZP_03749990.1, YP_002736886.1, YP_002746993.1, YP_002819418.1, ZP_04404310.1, ZP_04412054.1, ZP_04417835.1, ZP_04464399.1, ZP_04581218.1, ZP_04582926.1, ZP_04602815.1, ZP_04629248.1, YP_002924910.1, ZP_04680839.1, ZP_04752817.1, ZP_04753059.1, ZP_04754447.1, NP_716301.1, YP_003041946.1, ZP_04977481.1, ZP_04978949.1, ZP_05338643.1, NP_779194.1, NP_852755.1, NP_873071.1, NP_873952.1, NP_890165.1, NP_901808.1, NP_930133.1, NP_930658.1, NP_938235.1, ZP_00157240.2, YP_010340.1, YP_011919.1, YP_024703.1, YP_046794.1, YP_051809.1, YP_115319.1, YP_117734.1, YP_249190.1, ZP_00651743.1, ZP_00653061.1, ZP_00682996.1, AAZ67682.1, YP_389863.1, YP_421128.1, ZP_00944745.1, YP_454517.1, YP_550581.1, NP_050634.1

**Phage terminase-like protein:** YP_001038136.1, ZP_02170299.1, ZP_02171978.1, ZP_03462244.1, ZP_03462244.1, ZP_02041556.1, ZP_02041556.1, ZP_01966927.1, ZP_01966927.1, YP_001681403.1, YP_001681403.1, ZP_03152178.1, ZP_03152178.1, YP_001662881.1, YP_001662881.1, YP_001396026.1, YP_001396026.1, YP_002507111.1, YP_002507111.1, ZP_04019459.1, ZP_04019459.1, NP_976725.1, NP_976725.1, YP_001681478.1, YP_001681478.1, ZP_03782031.1, ZP_03782031.1, ZP_02430004.1, ZP_02430004.1, ZP_04023880.1, ZP_04023880.1, ZP_03974680.1, ZP_03974680.1, YP_181803.1, YP_181803.1, ZP_03973612.1, ZP_03973612.1, ABB02571.1, ABB02571.1, ZP_02418172.1, ZP_02418172.1, YP_001321774.1, YP_001321774.1, ZP_03993192.1, ZP_03993192.1, ZP_03923792.1, ZP_03923792.1, YP_329369.1, YP_329369.1, YP_060459.1, YP_060459.1, ZP_03928464.1, ZP_03928464.1, ZP_03168550.1, ZP_03168550.1, YP_001764586.1, NP_817458.1, YP_002014477.1, YP_752874.1, ZP_00787590.1, YP_001219870.1, YP_002470455.1, ZP_01047839.1, YP_116245.1, YP_002188758.1, ZP_02433115.1, ZP_00788409.1, ZP_00788409.1, ZP_02661965.1, ZP_03696771.1, YP_963861.1, YP_002358510.1, NP_718536.1, YP_263761.1, ZP_03801732.1, YP_002150681.1, ZP_03839624.1, YP_002548622.1, YP_002453334.1, ACQ68071.1, ZP_01099964.1, YP_179457.1, YP_002091327.1, YP_969977.1, ZP_04147253.1, NP_743720.1, YP_001748209.1, ZP_03070114.1, YP_001743247.1, YP_002575444.1, NP_746011.1, YP_002547884.1, YP_747683.1, ZP_04430138.1, YP_318229.1, YP_853044.1, YP_001884299.1, ZP_03842655.1, YP_001863187.1, YP_002254486.1, YP_318085.1, YP_001285808.1, ZP_03292421.1, NP_836845.1, YP_001743036.1, YP_688674.1, YP_002397735.1, ZP_03052620.1, ZP_03064165.1, NP_287339.1, YP_577730.1, YP_010722.1, YP_002407979.1, YP_002440138.1, NP_309625.1, ZP_03284796.1, YP_575689.1, NP_061498.1, ZP_03899692.1, YP_001437723.1, YP_577278.1, YP_352410.1, YP_107764.1, ZP_02373115.1, YP_654764.1, ZP_01788437.1, ZP_01768019.1, YP_002014328.1, ZP_02375777.1, YP_002239189.1, YP_006582.1, YP_001177321.1, AAF27357.1, ZP_02621261.1, YP_435681.1, YP_333046.1, ZP_02111898.1, YP_002284336.1, YP_001992960.1, ZP_02453429.1, YP_318158.1, YP_970129.1, YP_002507234.1, YP_002244037.1, YP_002216031.1, NP_700375.1, YP_001588383.1, YP_001189452.1, ZP_03045429.1, YP_002411643.1, YP_541972.1, YP_002115129.1, YP_785985.1, YP_001111081.1, YP_311631.1, YP_146381.1, ZP_03317859.1, ZP_02367142.1, YP_001344531.1, ZP_03804018.1, ZP_04520671.1, YP_002502374.1, NP_543088.1, ZP_03030857.1, YP_333082.1, NP_945032.1, NP_536358.1, AAC16226.1, YP_002338190.1, ZP_04147571.1, ZP_04227921.1, YP_030056.1, NP_846333.1, ZP_00394242.1, ZP_03236151.1, ZP_04121827.1, YP_078731.1, ZP_02389668.1, ZP_04271217.1, ZP_04591305.1, ZP_04235220.1, YP_642522.1, ZP_02950194.1, YP_002028887.1, ZP_04365119.1, ZP_01666651.1, AP_001775.1, YP_925839.1, YP_002339942.1, YP_002274971.1, YP_879378.1, YP_535676.1, YP_002224207.1, YP_656186.1, ZP_04265297.1, NP_348518.1, ZP_01963225.1, YP_002165905.1, NP_818312.1, ZP_04606583.1, YP_830503.1, YP_001406953.1, ZP_02185941.1, YP_001925379.1, YP_001491662.1, ZP_03816056.1, ZP_04383759.1, Q9F5P4.1, YP_001885143.1, ZP_04346152.1, NP_562034.1, ZP_02632545.1, YP_002125131.1, ZP_03886109.1, YP_002780413.1, NP_688845.1, ZP_02708664.1, ZP_01828913.1, AAN62835.1, YP_001429870.1, NP_061628.1, ZP_03890780.1, ZP_03890780.1, NP_058441.1, YP_716974.1, YP_716974.1, YP_099743.1, NP_959005.1, NP_959005.1, YP_002094030.1, YP_001765176.1, ZP_04334718.1, ZP_04334718.1, CAJ57175.1, CAJ57175.1, ZP_04127961.1, NP_823847.1, YP_001786830.1, YP_001155208.1, YP_253690.1, ZP_04321061.1, YP_956789.1, YP_956789.1, YP_001132150.1, YP_001132150.1, CAJ57158.1, ZP_03861786.1, YP_002181974.1, ZP_01968177.1, ZP_04030286.1, YP_891092.1, ZP_04527728.1, ZP_03051427.1, YP_001359430.1

**Recombinase A:** YP_639311.1, ABG72901.1, YP_679628.1, YP_706695.1, YP_715872.1, Q9REV6.1, Q9S660.1, YP_830954.1, ABK55850.1, ABK55851.1, ABK55854.1, ABK55856.1, ABK55862.1, ABK55863.1, ABK55864.1, ABK55865.1, ABK55867.1, ABK55868.1, ABK55874.1, ABK55876.1, ABK55883.1, ABK55884.1, ABK55896.1, ABK55900.1, ABK55901.1, CAL69150.1, CAL69152.1, CAL69155.1, CAL69168.1, CAL69169.1, YP_865525.1, YP_873250.1, YP_882804.1, YP_887057.1, YP_907029.1, YP_909887.1, YP_923533.1, YP_947360.1, YP_953239.1, 1G18, ZP_01667805.1, ZP_01693016.1, EAY55698.1, ZP_01719310.1, ZP_01724696.1, ZP_01733799.1, YP_001087824.1, YP_001103979.1, P48292.1, P48288.1, P48294.1, P48295.1, YP_001125268.1, CAM77326.1, YP_001135234.1, YP_001156610.1, YP_001158269.1, YP_001222767.1, CAM07008.1, CAM07009.1, CAM07010.1, CAM07011.1, CAM07012.1, CAM07013.1, CAM07014.1, CAM07015.1, CAM07017.1, CAM07018.1, CAM07019.1, CAM07020.1, CAM07021.1, CAM07023.1, CAM07024.1, CAM07025.1, CAM07026.1, CAM07027.1, CAM07029.1, CAM58517.1, ZP_01886144.1, YP_001361244.1, YP_001377655.1, ZP_01996439.1, ZP_02029656.1, ZP_02044715.1, NP_217253.1, NP_243249.1, ABV00483.1, ABV00484.1, ABV00491.1, YP_001486840.1, NP_301732.1, YP_001505579.1, YP_001510948.1, YP_001513081.1, YP_001529150.1, YP_001536280.1, NP_371809.1, YP_001546954.1, ZP_02062610.1, ZP_02080528.1, YP_001602540.1, ZP_02188100.1, YP_001623883.1, YP_001634976.1, CAP64295.1, ZP_02212530.1, ZP_02234546.1, YP_001680938.1, ZP_02424236.1, YP_001703792.1, YP_001692065.1, YP_001709945.1, YP_001800268.1, Q9F407.1, Q9F410.1, Q9F414.1, Q9F415.1, Q9F416.1, Q9F417.1, YP_001823257.1, ZP_02962733.1, YP_001850282.1, YP_001855437.1, YP_001917644.1, ZP_03011526.1, YP_001958363.1, ZP_03041591.1, ZP_03052970.1, ACF39382.1, NP_601162.1, BAG68221.1, ZP_03146260.1, YP_002140418.1, YP_002136028.1, YP_002136409.1, ACH56443.1, ACH56444.1, ACH56445.1, ZP_03168226.1, ZP_03226272.1, EDZ38784.1, YP_002274922.1, YP_002297142.1, ZP_03293286.1, NP_629894.1, YP_002315881.1, ZP_03323753.1, YP_002322426.1, ZP_03392686.1, ZP_03426083.1, ZP_03429592.1, ZP_03433740.1, 2ZR0, 2ZRC, 2ZRH, ZP_03446193.1, YP_002432103.1, ZP_03537818.1, ZP_03537819.1, YP_002459565.1, YP_002463084.1, YP_002487540.1, YP_002494492.1, YP_002508934.1, ACL92954.1, ACL92962.1, ACL92965.1, ACL92967.1, ACL92970.1, ACL92973.1, ACL92984.1, ACL92990.1, ZP_03591416.1, ZP_03604382.1, YP_002519261.1, ZP_03619437.1, ZP_03646717.1, YP_002603990.1, ZP_03682629.1, ZP_03690764.1, ZP_03710392.1, ZP_03728585.1, ZP_03731491.1, ZP_03742370.1, ZP_03758236.1, YP_002756306.1, CAJ82777.1, CAJ82768.1, CAJ82770.1, CAJ82771.1, CAJ82773.1, CAJ82775.1, YP_002766200.1, YP_002772877.1, YP_002783943.1, ZP_03815608.1, ZP_03856552.1, ZP_03866158.1, ZP_03867167.1, ZP_03886534.1, ZP_03891164.1, ZP_03912263.1, ZP_03919009.1, ZP_03924099.1, ZP_03925483.1, ZP_03926124.1, ZP_03933488.1, ZP_03934909.1, ZP_03937323.1, ZP_03940233.1, ZP_03954376.1, ZP_03957348.1, ZP_03979129.1, ZP_03989471.1, YP_002835031.1, ZP_03993363.1, ZP_04019071.1, ZP_04026720.1, ZP_04031426.1, ZP_04043140.1, ZP_04152395.1, ZP_04160143.1, ZP_04198728.1, ZP_04333739.1, ZP_04348647.1, ZP_04356324.1, ZP_04367435.1, ZP_04381439.1, ZP_04384695.1, ZP_04424040.1, ZP_04433423.1, ZP_04447719.1, YP_002882460.1, ZP_04476862.1, ZP_04481235.1, ZP_04494335.1, ZP_04507484.1, YP_002884806.1, NP_692545.1, NP_696578.1, ZP_04532623.1, YP_002906404.1, ZP_04608162.1, YP_002938101.1, ZP_04661993.1, CAX83753.1, YP_002956809.1, ZP_04685508.1, ZP_04690456.1, ZP_04696687.1, ZP_04705218.1, ZP_04711879.1, ZP_04749846.1, ZP_04786612.1, ZP_04806592.1, Q59717.1, CAD56684.1, NP_738459.1, P16971.2, YP_003012100.1, ZP_04834497.1, ZP_04850941.1, ZP_04853362.1, YP_003023512.1, ACT64225.1, ZP_04926163.1, ZP_04990932.1, ZP_05012943.1, ZP_05124170.1, ZP_05142239.1, ZP_05226761.1, ZP_05311629.1, ZP_05334867.1, ZP_05366281.1, ZP_05367445.1, ZP_05374182.1, YP_003093810.1, ZP_05400748.1, ZP_05405213.1, CAA03857.1, AAC38570.1, NP_785779.1, NP_787739.1, NP_789554.1, NP_816775.1, NP_823668.1, AAP49230.1, AAP49231.1, AAP49232.1, AAP49233.1, AAP49234.1, AAP49236.1, AAP49237.1, AAP49238.1, AAP49239.1, AAP49240.1, AAP49241.1, AAP49242.1, AAP49243.1, AAP49244.1, AAP49245.1, P95846.1, NP_939798.1, NP_961782.1, AAS16314.1, AAS16315.1, AAS16317.1, AAS16318.1, AAS16319.1, AAS16320.1, AAS16323.1, AAS16324.1, AAS16325.1, AAS16326.1, AAS16328.1, AAS16329.1, AAS16330.1, AAS16331.1, ZP_00206783.1, ZP_00056375.2, CAA54563.1, AAT35784.1, AAT35785.1, AAT35787.1, AAT35788.1, AAT35789.1, AAT35790.1, AAT35791.1, AAT35792.1, AAT35793.1, AAT35794.1, AAT35796.1, AAT35797.1, AAT35798.1, AAT35799.1, AAT35800.1, AAT35801.1, AAT35803.1, AAT35804.1, AAT35805.1, CAF21827.1, CAF21829.1, CAF21837.1, CAF21840.1, AAD29645.1, AAT69989.1, AAT69994.1, YP_055722.1, YP_062501.1, YP_079090.1, YP_120055.1, YP_175695.1, YP_191920.1, Q08327.1, Q6AE00.2, ZP_00378192.1, AAY41901.1, AAY41902.1, YP_250898.1, YP_288864.1, P41054.1, , Q6KCK0.1, YP_342966.1, ABB29452.1, ABB29469.1, YP_419985.1, YP_429936.1, ZP_00995747.1, ZP_01004742.1, YP_467165.1, YP_482607.1, YP_496553.1, ZP_01128342.1, ZP_01130098.1, ZP_01173570.1, CAJ57510.1, CAJ57516.1, CAJ57517.1, CAJ57531.1, CAJ57534.1, CAJ57524.1, YP_518176.1, ABF06626.1, ZP_01303090.1, YP_589337.1, ZP_01311355.1

**Ribonucleotide reductase, class I + II:** YP_742310.1, ZP_01739863.1, ZP_03691930.1, YP_002300245., ZP_01035938.1, YP_969044.1, YP_551369.1, YP_114954.1, YP_001717412., ZP_03495706.1, ZP_04037118.1, NP_296095.1, YP_001516707., NP_926912.1, YP_658568.1, ZP_03999548.1, YP_002567020., YP_003177587., YP_136243.1, YP_327319.1, YP_003130199., NP_280419.1, ZP_03694849.1, ABB77927.1, YP_876684.1, YP_001582961., CAF28708.1, NP_578169.1, YP_184149.1, YP_001736577., YP_001153873., YP_001794977., YP_002955841., ZP_01288839.1, ZP_05336021.1, YP_516555.1, AAU84278.1, YP_359531.1, ZP_05570431.1, XP_001225859., XP_001909942., XP_370503.1, ABF71875.1, EEU45879.1, XP_385350.1, XP_001267356., XP_752157.1, XP_002565681., XP_002341068., EEH48431.1, EEQ78211.1, EEQ28424.1, EER26138.1, XP_002583836., XP_001795228., XP_001934447., XP_001549288., XP_001588490., EDK38820.2, BAD96506.1, XP_001700540., YP_001209517., ZP_05704510.1, ZP_02478768.1, ZP_04978627.1, ZP_00348265.1, YP_003007303., YP_003175795., YP_327711.1, YP_002564381., ABB77922.1, YP_444285.1, ZP_03497649.1, YP_706374.1, YP_120520.1, YP_638982.1, ZP_03887216.1, ZP_04028672.1, YP_240947.1, ABC79615.1, YP_001536302., ZP_04608183.1, ZP_04480858.1, ZP_04478040.1, YP_290205.1, YP_873234.1, YP_482589.1, ZP_05478366.1, YP_003134565., YP_001104006., YP_003099228., YP_003203257., ZP_03891185.1, YP_924998.1, ZP_03866343.1, YP_003149495., YP_003112579., YP_002754665., YP_590201.1, YP_822940.1, YP_002134603., YP_002049452., ZP_05045409.1, NP_875207.1, NP_682117.1, YP_400626.1, ZP_05023767.1, ZP_01632057.1, NP_488075.1, YP_322188.1, ZP_03765843.1, ZP_05037241.1, YP_720358.1,

**Ribonucleotide reductase, anaerobic:** YP_695371.1, YP_754935.1, AAG37223.1, YP_843683.1, YP_876686.1, YP_916768.1, ZP_01667058.1, YP_001037074.1, YP_001046222.1, YP_001046235.1, YP_001052876.1, YP_001097982.1, YP_001112057.1, YP_001130324.1, YP_001179584.1, YP_001209802.1, YP_001212392.1, YP_001212704.1, YP_685421.1, YP_001244134.1, YP_001273956.1, YP_001307214.1, YP_001305856.1, YP_001323750.1, YP_001325231.1, YP_001330441.1, YP_001404869.1, YP_001410753.1, ZP_02041959.1, NP_228195.1, NP_247823.1, NP_248037.1, NP_276652.1, YP_001434627.1, YP_001470063.1, YP_001474539.1, YP_001513596.1, YP_001530450.1, NP_347120.1, NP_347841.1, YP_001548777.1, YP_001568023.1, ZP_02193841.1, YP_001664653.1, YP_001662776.1, ABZ07645.1, ABZ08630.1, ABZ09207.1, ABZ09455.1, ABZ09902.1, ZP_02420594.1, ZP_02431419.1, ZP_02478622.1, YP_001718118.1, ZP_02868810.1, YP_001738587.1, ZP_02900187.1, ZP_02900200.1, ZP_02638069.2, ZP_02641835.2, ZP_02952790.1, NP_561833.1, ACC86126.1, YP_001876077.1, YP_001884381.1, YP_001919565.1, YP_001943105.1, YP_001956525.1, YP_001959606.1, YP_001967960.1, YP_001991628.1, YP_001995601.1, YP_002015596.1, YP_002019400.1, YP_002122064.1, YP_002128166.1, YP_002220748.1, NP_615046.1, NP_614184.1, YP_002248349.1, YP_002246379.1, YP_002251404.1, NP_623435.1, ZP_03290622.1, NP_633391.1, XP_002174124.1, ZP_03391235.1, YP_002334493.1, YP_002353584.1, YP_002431473.1, YP_002435889.1, YP_002436231.1, YP_001741319.1, YP_002465128.1, YP_002475724.1, YP_002508853.1, YP_002533854.1, ZP_03608198.1, YP_002572447.1, ZP_03611940.1, ZP_03635348.1, ZP_03635422.1, YP_002601763.1, ZP_03681608.1, ZP_03703986.1, ZP_03713764.1, ZP_03718422.1, ZP_03737098.1, ZP_03750797.1, ZP_03757779.1, ZP_03778162.1, ZP_03906757.1, ZP_04058522.1, ZP_04340743.1, ZP_04468951.1, ZP_04602165.1, YP_002940254.1, ZP_04668672.1, YP_002955849.1, ZP_04752967.1, ZP_04756940.1, ZP_04759850.1, ZP_04763455.1, ZP_04774506.1, ZP_04789601.1, YP_002992546.1, YP_002993099.1, ZP_04822904.1, ZP_04863664.1, ZP_04873430.1, ZP_04875739.1, ZP_04879798.1, ZP_04978549.1, ZP_05132532.1, ZP_05303374.1, ZP_05319981.1, ZP_05332620.1, ZP_05336555.1, ZP_05379159.1, ZP_05395116.1, EEU34719.1, ZP_05492984.1, YP_003128240.1, YP_003140713.1, YP_003146402.1, YP_003159885.1, YP_003162147.1, YP_003130508.1, ZP_05621282.1, YP_003183067.1, YP_003196948.1, YP_003190580.1, YP_003191347.1, YP_003193220.1, ZP_05705211.1, ACV74833.1, ZP_05709245.1, NP_780965.1, ZP_00134686.1, NP_872972.1, NP_969395.1, NP_971937.1, NP_987347.1, XP_383202.1, YP_009523.1, YP_012159.1, YP_063800.1, AAU82120.1, AAU82145.1, AAU82212.1, AAU82293.1, AAU83393.1, AAU83530.1, AAU83764.1, AAU85395.1, YP_154580.1, YP_162760.1, YP_266860.1, YP_304585.1, YP_360870.1, YP_386773.1, YP_389505.1, YP_398506.1, YP_430302.1, YP_426364.1, YP_447313.1, CAJ57167.1, YP_461290.1, YP_461909.1, YP_504224.1, YP_533376.1, CAJ72475.1, YP_566748.1, YP_566760.1, ZP_01313785.1

**RNA terminal phosphate cyclase:** YP_628347.1, YP_633573.1, YP_629773.1, YP_747106.1, NP_069696.1, ZP_01459598.1, ZP_01464376.1, CAL56150.1, YP_846839.1, YP_843789.1, NP_148148.2, YP_876007.1, YP_901808.1, ZP_01622715.1, YP_919682.1, YP_930729.1, YP_001003920.1, XP_001305113.1, XP_001310135.1, XP_001320361.1, YP_001012725.1, CAM32979.1, YP_001029573.1, XP_001347744.1, XP_001356267.1, YP_001047560.1, YP_001055045.1, YP_001040992.1, YP_001096718.1, NP_111841.1, BAB60487.1, NP_125803.1, NP_126251.1, YP_001152439.1, YP_001159224.1, NP_143457.1, YP_001191831.1, YP_001214204.1, YP_001212132.1, YP_684757.1, NP_001090369.1, YP_001230167.1, YP_001245015.1, YP_001250948.1, YP_001273239.1, YP_001306121.1, YP_001323221.1, YP_001324338.1, YP_001325500.1, YP_001329855.1, YP_001378179.1, YP_001404951.1, YP_001410193.1, XP_001581694.1, NP_213010.1, XP_001615186.1, NP_229158.1, XP_001607419.1, NP_247666.1, NP_248036.1, NP_248426.1, NP_276709.1, YP_001434983.1, YP_001470556.1, XP_001667680.1, NP_280280.1, NP_337208.1, YP_001530451.1, NP_342450.1, YP_001537381.1, YP_001540160.1, YP_001541671.1, NP_377227.1, YP_001543588.1, YP_001549326.1, NP_393759.1, ZP_02065448.1, ZP_02086264.1, ZP_02088965.1, YP_001582787.1, YP_001612593.1, ZP_02177476.1, ZP_02193184.1, ZP_02203264.1, ZP_01947267.2, ABZ07514.1, XP_001745425.1, ZP_02355619.1, ZP_02362759.1, ZP_02370843.1, ZP_02384727.1, ZP_02549552.1, ZP_02733055.1, YP_001716655.1, XP_001870125.1, YP_001737836.1, YP_001793560.1, NP_492498.1, NP_559005.1, YP_001869633.1, YP_001938963.1, YP_001949958.1, NP_579344.1, ABO13245.2, YP_001998238.1, XP_001947227.1, XP_001963238.1, XP_001974258.1, XP_001992790.1, XP_002002144.1, XP_002039109.1, XP_002057666.1, XP_002064417.1, XP_002090700.1, XP_002079923.1, XP_002108933.1, ZP_03157798.1, YP_002137806.1, YP_002133371.1, NP_615239.1, NP_613597.1, NP_614965.1, YP_002249541.1, YP_002246985.1, YP_002251661.1, YP_001424621.2, YP_002303374.1, YP_002305386.1, YP_002307099.1, NP_633562.1, , ZP_03416770.1, ZP_03433640.1, NP_661501.1, YP_002334926.1, YP_002352073.1, YP_002371855.1, ZP_03496783.1, YP_002380020.1, YP_002428903.1, ZP_03537711.1, YP_002466089.1, YP_002491421.1, XP_002259057.1, YP_002523907.1, YP_002537776.1, YP_002534776.1, YP_002566672.1, ZP_03629450.1, ZP_03694489.1, ZP_03699999.1, ZP_03703896.1, ZP_03711909.1, ZP_03730440.1, ZP_03738794.1, ZP_03762420.1, YP_002773410.1, YP_002775316.1, EEH60882.1, XP_002503505.1, YP_002801699.1, ZP_03856856.1, ZP_03860531.1, YP_002829310.1, ZP_03999648.1, ZP_04422893.1, ZP_04427857.1, YP_002837430.1, YP_002840616.1, ZP_04480971.1, ZP_04495526.1, ZP_04545122.1, ZP_04548574.1, NP_609965.1, NP_856310.1, NP_901095.1, NP_952735.1, NP_963373.1, NP_988512.1, NP_998268.1, XP_393151.1, 1UC2, YP_023383.1, AAU43700.1, AAU82698.1, AAU83124.1, AAU83326.1, AAU83551.1, AAU84293.1, YP_096213.1, YP_112731.1, YP_127460.1, YP_124463.1, YP_181550.1, YP_182771.1, XP_317316.2, ZP_00378105.1, XP_642393.1, XP_648794.1, YP_255946.1, YP_260686.1, XP_764297.1, NP_001025674.1, YP_289418.1, YP_304796.1, YP_307866.1, YP_316117.1, YP_326741.1, YP_344001.1, YP_355452.1, YP_359049.1, YP_384575.1, YP_388581.1, YP_411598.1, YP_439510.1, YP_446591.1, YP_447682.1, XP_953305.1, YP_460053.1, YP_464158.1, YP_475920.1, YP_504582.1, ABD75810.1, YP_525082.1, XP_969671.1, CAJ72931.1, YP_566822.1, O27634.2

**SNF2/Rad54 helicase:** YP_642522.1, YP_690085.1, YP_879378.1, YP_925839.1, ZP_01666651.1, YP_001038136.1, YP_001155208.1, YP_001491662.1, NP_294982.1, NP_294983.1, ZP_02171978.1, YP_001765176.1, YP_001648921.1, Q9F5P4.1, YP_002014477.1, ZP_03497306.1, ZP_03815780.1, ZP_03816056.1, ZP_04346152.1, ZP_04365119.1, YP_002966683.1, AAN62835.1, ZP_04941340.1, ZP_04998052.1, ZP_05214640.1, NP_817458.1, NP_818312.1, NP_823847.1, YP_224270.1, YP_418072.1, CAJ57158.1, CAJ57176.1, YP_549845.1

**Threonyl-tRNA synthetase:** XP_001086926.1, YP_662069.1, YP_658297.1, YP_706802.1, YP_710168.1, YP_734085.1, YP_738067.1, YP_748513.1, YP_750712.1, YP_753770.1, XP_517835.2, XP_001153847.1, XP_001153909.1, XP_001153418.1, XP_001153291.1, XP_001153674.1, XP_001212429.1, CAL54387.1, ZP_01472711.1, XP_001219623.1, XP_001220095.1, YP_864295.1, XP_413774.2, YP_877833.1, YP_882672.1, YP_894938.1, YP_901973.1, YP_906934.1, XP_001248022.1, XP_001266681.1, EAX10803.1, YP_927640.1, YP_932583.1, YP_945706.1, YP_959329.1, YP_963382.1, ZP_01667441.1, ZP_01678683.1, XP_001272218.1, CAM13348.1, YP_001006170.1, ABM82342.1, ZP_01708041.1, YP_001037653.1, YP_001050514.1, XP_001373323.1, XP_001370758.1, XP_001373481.1, ZP_01722511.1, ZP_01738865.1, YP_001087049.1, BAB26799.1, YP_001100081.1, YP_001104239.1, ZP_01768717.1, YP_001112965.1, AAH04621.1, ZP_01771191.1, NP_116578.1, AAA22863.1, YP_001135098.1, XP_001389370.1, XP_001418868.1, XP_001441555.1, XP_001450463.1, XP_001452975.1, XP_001468953.1, XP_001017218.2, YP_001172865.1, YP_001183538.1, YP_001179752.1, YP_001191664.1, YP_001176454.1, XP_001486012.1, AAB04939.1, YP_001215778.1, YP_001212506.1, NP_001087812.1, YP_001243794.1, YP_001222563.1, YP_001255628.1, ABQ75826.1, EDL03283.1, ABR17634.1, EDL82977.1, EDL82978.1, EDM08408.1, ZP_01859377.1, ZP_01872516.1, XP_001524878.1, XP_001508543.1, XP_001513531.1, ZP_01915136.1, YP_001310292.1, YP_001306561.1, YP_001319467.1, AAK82468.1, XP_001385328.2, EDN61420.1, YP_001335838.1, YP_001375062.1, YP_001353177.1, YP_001355582.1, ZP_01967232.1, ZP_01980216.1, ZP_01996815.1, YP_001392415.1, YP_001397450.1, YP_001396575.1, YP_001410237.1, XP_001539849.1, XP_001548242.1, XP_001550563.1, XP_001568243.1, ZP_02034685.1, ZP_02039877.1, NP_212854.1, NP_232683.1, XP_001587735.1, NP_214149.1, XP_001610740.1, XP_001615376.1, XP_001623293.1, NP_228549.1, CAB02510.1, XP_001603180.1, NP_001096223.1, XP_001642915.1, XP_001658309.1, YP_001463015.1, YP_001471606.1, YP_001473857.1, YP_001481771.1, YP_001486007.1, XP_001679182.1, NP_280563.1, YP_001502118.1, NP_288153.1, XP_319682.4, YP_001513514.1, YP_001531074.1, NP_348978.1, NP_343832.1, NP_376876.1, XP_001696198.1, YP_001554758.1, YP_001559765.1, YP_001567882.1, ZP_02084362.1, ZP_02093357.1, NP_416234.1, YP_001588225.1, YP_583315.2, ZP_02159762.1, ZP_02177014.1, YP_001645041.1, XP_001729744.1, ZP_02211696.1, YP_001651268.1, ZP_02234861.1, YP_001669708.1, YP_001664759.1, YP_001663665.1, XP_001739012.1, XP_001750114.1, NP_456179.1, YP_001674240.1, YP_001676832.1, YP_001680356.1, NP_460299.1, ZP_02418862.1, ZP_02422007.1, ZP_02431032.1, ZP_02438989.1, ZP_02441421.1, ZP_02494347.1, XP_001784310.1, ZP_02613877.1, ZP_02618121.1, ZP_02622643.1, ZP_02658309.1, YP_001689681.1, ZP_02861731.1, XP_001802251.1, YP_001703631.1, XP_001816697.1, YP_001691999.1, YP_001698346.1, YP_001717525.1, XP_001841372.1, XP_001845897.1, XP_001878968.1, YP_001738230.1, YP_001737367.1, XP_001893343.1, YP_001748839.1, YP_001782772.1, YP_001788457.1, ZP_02903316.1, YP_001709474.1, ZP_02918713.1, YP_204597.2, NP_488763.1, ZP_02950872.1, YP_001850393.1, ZP_02993413.1, YP_001884138.1, ZP_02960307.2, YP_001917984.1, YP_001930308.1, BAG38072.1, XP_001929781.1, XP_001936638.1, XP_001335155.2, EDV09560.1, NP_596364.1, NP_594034.1, ZP_03037248.1, ZP_03046830.1, XP_001950747.1, BAG53144.1, ZP_03056511.1, XP_001491149.2, XP_001498225.2, BAG57079.1, BAG59361.1, XP_001965104.1, XP_001969762.1, XP_001989177.1, XP_002002014.1, XP_002014541.1, XP_002042027.1, XP_002052626.1, XP_002069231.1, XP_002088448.1, XP_002079196.1, ZP_03086920.1, XP_002116349.1, ZP_03101262.1, ZP_03107334.1, ZP_03110657.1, NP_001125825.1, YP_002150784.1, YP_002220653.1, CAR65481.1, YP_002226745.1, YP_002237974.1, YP_002250745.1, ZP_03232370.1, ZP_03235015.1, EDZ71519.1, NP_623309.1, ZP_03282208.1, ZP_03293938.1, ZP_03297958.1, CAP97671.1, XP_002147528.1, YP_002311792.1, ZP_03304635.1, ZP_03316599.1, ZP_03319565.1, XP_002172490.1, ZP_03380918.1, YP_002322621.1, AAM50188.1, ZP_03436486.1, YP_002335447.1, YP_002352919.1, YP_002367070.1, YP_002382501.1, NP_001116258.1, YP_002433364.1, YP_002445747.1, XP_002186340.1, AAB61048.1, XP_002219850.1, XP_002236065.1, AAI43944.1, YP_002456689.1, ZP_03539428.1, ZP_03540326.1, YP_002505706.1, YP_002513522.1, XP_002259263.1, XP_002164939.1, ZP_03589433.1, NP_669219.1, ZP_03593566.1, YP_002538809.1, YP_002530031.1, YP_002535386.1, YP_002574647.1, NP_671778.1, ZP_03619149.1, ZP_03637968.1, XP_002290729.1, XP_002195014.1, XP_002193839.1, YP_002604988.1, ZP_03655835.1, ZP_03672248.1, ZP_03673009.1, ZP_03675058.1, ZP_03691265.1, ZP_03693983.1, ZP_03717846.1, ZP_03730427.1, ZP_03735101.1, ZP_03737996.1, ZP_03757296.1, ZP_03769908.1, ZP_03772775.1, EEH09084.1, ZP_03777729.1, YP_002720925.1, EEH22340.1, YP_002729587.1, YP_002749728.1, EEH34054.1, EEH48870.1, YP_002766350.1, YP_002771085.1, ZP_03796944.1, ZP_03800041.1, YP_002784049.1, EEH58234.1, ZP_03821363.1, YP_002805659.1, ZP_03831069.1, ZP_03855767.1, ZP_03874695.1, ZP_03886624.1, ZP_03902705.1, ZP_03906726.1, ZP_03916013.1, ZP_03924304.1, ZP_03928681.1, ZP_03931100.1, ZP_03946511.1, YP_002828459.1, YP_002831105.1, ZP_03990397.1, ZP_04019139.1, NP_758514.2, ZP_04045056.1, ZP_04046857.1, ZP_04050326.1, ZP_04065194.1, ZP_04071981.1, ZP_04084417.1, ZP_04096525.1, ZP_04102109.1, ZP_04120356.1, ZP_04126449.1, ZP_04145631.1, ZP_04154183.1, ZP_04157073.1, ZP_04168819.1, ZP_04174581.1, ZP_04191800.1, ZP_04197403.1, ZP_04212097.1, ZP_04217535.1, ZP_04222576.1, ZP_04227833.1, ZP_04233661.1, ZP_04245250.1, ZP_04262049.1, ZP_04273357.1, ZP_04278801.1, ZP_04284064.1, ZP_04289318.1, ZP_04294942.1, ZP_04300617.1, ZP_04306091.1, ZP_04317445.1, ZP_04323345.1, ZP_04341157.1, ZP_04342845.1, ZP_04350837.1, ZP_04352206.1, ZP_04363882.1, ZP_04385789.1, ZP_04420595.1, ZP_04422772.1, YP_002842345.1, ZP_04445493.1, ZP_04454813.1, NP_695905.1, ZP_04561750.1, ZP_04578147.1, ZP_04583392.1, ZP_04584494.1, EEP80648.1, ZP_04599420.1, XP_002383381.1, YP_002913592.1, ZP_04610987.1, ZP_04615048.1, ZP_04626223.1, ZP_04627407.1, ZP_04630889.1, ZP_04640880.1, EEQ30510.1, EEQ41676.1, EEQ46063.1, YP_002919970.1, YP_002933297.1, ZP_04658872.1, CAR21241.1, EEQ69572.1, EEQ85827.1, YP_002941770.1, ZP_04666209.1, EER03601.1, EER09238.1, EER15070.1, EER15071.1, EER17786.1, YP_002957133.1, EER23329.1, EER30782.1, ZP_04749316.1, EER44156.1, ZP_04756343.1, XP_002412596.1, ZP_04767549.1, XP_002420321.1, XP_002432585.1, XP_002460938.1, NP_711421.1, YP_002987664.1, ZP_04801825.1, ZP_04807464.1, YP_002990950.1, ZP_04809097.1, XP_002481676.1, NP_717895.1, NP_723725.1, NP_723726.1, AAH39225.1, AAN71609.1, YP_003004279.1, YP_003009768.1, ZP_04829591.1, ZP_04862922.1, YP_003017465.1, YP_003040702.1, ZP_05045556.1, ZP_05070408.1, ZP_05082281.1, ZP_05104620.1, XP_002491958.1, XP_002496040.1, YP_003056945.1, ZP_05224891.1, ZP_05291997.1, ZP_05307656.1, XP_002499537.1, ZP_05332471.1, ZP_05335029.1, ZP_05345330.1, ZP_05349734.1, ZP_05380091.1, ZP_05392970.1, ZP_05400002.1, ZP_05405107.1, BAC27235.1, NP_744613.1, NP_149065.2, AAN87390.1, NP_761244.1, NP_782828.1, NP_832085.1, NP_841031.1, NP_844767.1, NP_198035.2, NP_859973.1, AAH55371.1, CAA74705.1, NP_929904.1, NP_934738.1, NP_689508.3, NP_689547.2, XP_361008.1, NP_952566.1, NP_961650.1, NP_978728.1, CAA23560.1, NP_983365.1, YP_002393.1, XP_381424.1, CAA26666.1, ZP_00120366.2, YP_009014.1, CAG03123.1, CAG08617.1, ZP_00236782.1, NP_001001219.1, CAE02700.1, YP_036488.1, YP_050517.1, XP_449908.1, XP_455348.1, XP_457398.1, XP_499902.1, XP_505278.1, YP_073160.1, YP_076387.1, YP_077489.1, YP_083736.1, YP_088245.1, AAH10578.2, ZP_00134603.2, YP_113204.1, NP_001005932.1, NP_001006977.1, YP_150756.1, Q7MK65.2, AAH88355.1, YP_177470.1, YP_178222.1, ZP_00372076.1, XP_569057.1, AAH90718.1, NP_001014042.1, YP_216339.1, BAD94986.1, BAD96524.1, ZP_00392643.1, XP_628350.1, XP_395662.2, XP_653365.1, XP_663266.1, XP_666722.1, ZP_00513735.1, XP_677134.1, XP_710514.1, YP_255899.1, YP_252049.1, XP_751461.1, XP_760995.1, XP_766478.1, XP_811024.1, XP_816364.1, NP_001026618.1, YP_285877.1, NP_001022033.1, NP_001022034.1, XP_843247.1, XP_857231.1, XP_536172.2, XP_536509.2, XP_860710.1, XP_860741.1, XP_860781.1, XP_850160.1, XP_860843.1, BAE31895.1, ZP_00744025.1, YP_320127.1, Q4J9C4.2, YP_326856.1, NP_001029542.1, YP_356834.1, YP_360408.1, YP_384370.1, YP_389130.1, YP_392582.1, CAI78599.1, YP_407826.1, YP_411187.1, XP_727721.1, YP_403416.1, YP_430603.1, YP_435707.1, XP_953817.1, XP_959801.1, YP_460699.1, ZP_01067964.1, ZP_01100547.1, ZP_01125097.1, YP_516473.1, XP_967345.1, YP_546111.1, YP_562712.1, ABF08046.1

**NAHD dehydrogenase subunit 1:** YP_626454.1, YP_626507.1, YP_626481.1, AAG23680.1, YP_635803.1, YP_636932.1, YP_636919.1, YP_636524.1, YP_636374.1, YP_636354.1, YP_654086.1, Q2QD36.1, P92432.4, Q6L3D5.2, YP_654394.1, YP_654276.1, YP_654355.1, YP_654407.1, YP_654342.1, YP_654316.1, YP_654381.1, YP_654329.1, P25706.3, Q6ENA2.2, Q95H43.2, YP_665670.1, YP_665698.1, YP_665611.1, CAH59766.1, CAH59769.1, CAH59772.1, CAH59782.1, CAH59788.1, YP_667825.1, YP_684410.1, YP_717098.1, YP_717293.1, YP_721332.1, YP_725566.1, YP_731813.1, YP_740257.1, YP_740172.1, YP_745116.1, YP_740620.1, YP_740706.1, NP_049303.1, NP_046146.1, NP_054562.1, NP_038449.1, NP_038180.1, NP_044754.1, NP_066473.1, NP_039353.1, NP_039449.1, NP_042570.1, NP_054441.1, NP_043092.1, NP_043742.1, NP_057982.1, YP_762315.1, NP_042269.1, NP_054993.1, YP_762480.1, YP_762697.1, YP_778546.1, YP_778639.1, YP_783944.1, YP_784442.1, YP_784523.1, YP_817537.1, ABK35785.1, YP_867519.1, YP_874792.1, YP_874707.1, YP_903497.1, YP_899462.1, YP_913510.1, YP_913692.1, YP_913562.1, YP_913354.1, YP_913666.1, YP_913484.1, YP_913241.1, ZP_01632574.1, YP_981666.1, YP_997562.1, YP_001004234.1, YP_001010505.1, YP_001008573.1, YP_001018686.1, YP_001014062.1, NP_074956.1, YP_001001589.2, YP_001019162.1, YP_001020607.1, YP_001023656.1, YP_001023756.1, YP_001023792.1, YP_001023807.1, YP_001023828.1, YP_001031200.1, Q1ACE4.2, ABO20803.1, YP_001090221.1, YP_001090404.1, CAA23994.1, NP_075424.1, P08774.1, YP_001109562.1, YP_001100099.1, NP_084850.1, P48899.1, YP_001123430.1, YP_001122852.1, YP_001123342.1, YP_001123694.1, YP_001123870.1, YP_001123085.1, YP_001123170.1, YP_001123255.1, YP_001123519.1, YP_001123606.1, YP_001123782.1, YP_001123001.1, NP_114313.1, CAM77657.1, YP_001155824.1, YP_001165407.1, YP_001165360.1, YP_001234066.1, YP_001249252.1, YP_001249337.1, BAA99457.2, ABR09223.1, YP_001294148.1, YP_001294403.1, YP_001294326.1, YP_001294240.1, YP_001312271.1, YP_001315135.1, NP_150120.1, NP_150385.1, CAA69811.3, YP_001353159.1, YP_001381679.1, ABS50622.1, YP_001419503.1, YP_001414482.1, NP_221146.1, ABU50158.1, YP_001427399.1, ABU85138.1, ABU85282.1, ABU85358.1, ABU85400.1, ABU85451.1, ABU85524.1, ABU85596.1, ABU85711.1, A2CD61.2, A1VM67.2, YP_001483383.1, YP_001492717.1, YP_001492833.1, YP_001493970.1, YP_001496820.1, YP_001495304.1, YP_001499768.1, YP_001504349.1, YP_001516486.1, Q2IL15.2, Q8DL32.2, Q7V4D7.2, Q7VE30.2, NP_360867.1, CAO90419.1, NP_009259.2, YP_001531337.1, YP_001542502.1, YP_001547743.1, YP_001550062.1, ABX45148.1, CAA31563.1, YP_001595564.1, ABX82067.1, YP_001603265.1, NP_442814.1, NP_443554.1, ZP_02187170.1, YP_001630292.1, YP_001648474.1, YP_001648520.1, YP_001648590.1, YP_001648603.1, YP_001648492.1, YP_001648542.1, YP_001648483.1, YP_001648674.1, YP_001648627.1, YP_001648547.1, YP_001648741.1, ABY55193.1, NP_084756.2, YP_001660659.1, YP_001661381.1, YP_001661423.1, YP_001671737.1, ZP_02462939.1, YP_001687115.1, YP_001687191.1, YP_001687337.1, YP_001687505.1, YP_001718491.1, YP_001734186.1, YP_001718703.1, ACB38122.1, ACB38125.1, ACB38127.1, YP_001790541.1, YP_001797646.1, YP_001806702.1, NP_484267.1, NP_520176.1, YP_001837410.1, NP_570160.1, YP_001868872.1, YP_001874782.1, YP_001874840.1, YP_001876505.1, YP_785572.1, YP_001876523.1, YP_001899773.1, ACD44965.1, ACD44986.1, ACD44987.1, ACD45046.1, ACD46627.1, NP_569690.1, YP_001936198.1, YP_001938283.1, ACD85990.1, YP_001977836.1, YP_002000540.1, YP_002000548.1, YP_002005073.1, YP_002122385.1, ABZ79354.2, ACH47250.1, ACH47251.1, ACH47252.1, ACH47253.1, ACH47254.1, YP_002169566.1, YP_002213605.1, ACH78245.1, YP_002220660.1, YP_002221404.1, NP_612817.1, BAB92036.1, BAB92039.1, BAB92040.1, ACI22632.1, YP_002265497.1, YP_002274326.1, YP_002280788.1, YP_002297458.1, NP_659254.1, AAL65276.1, YP_002364553.1, YP_002482396.1, YP_002491784.1, YP_002499314.1, YP_002519625.1, YP_002519690.1, YP_002519716.1, YP_002519794.1, YP_002519833.1, BAH15233.1, YP_002586790.1, YP_002586842.1, YP_002586868.1, YP_002587023.1, NP_681456.1, YP_002608183.1, YP_002600965.1, YP_002608338.1, YP_002592062.1, YP_002616934.1, ZP_03697923.1, YP_002670015.1, YP_002712025.1, ZP_03741375.1, NP_689379.1, ZP_03763947.1, YP_002720167.1, EEH02508.1, EEH02524.1, ZP_03788067.1, ACO50738.1, NP_683857.1, ZP_03907206.1, YP_002860122.1, YP_002860269.1, YP_002836145.1, YP_002845659.1, NP_696969.1, ZP_04577224.1, YP_002916671.1, EEQ69176.1, ZP_04698566.1, P06254.3, YP_002970815.1, YP_002970843.1, YP_002970871.1, YP_002970787.1, YP_002970899.1, EER36336.1, NP_705917.1, XP_002408505.1, YP_002981837.1, ZP_04761352.1, YP_002970700.1, Q37165.1, BAC23919.1, NP_739841.1, CAA27295.1, Q00242.1, Q01300.1, Q01148.1, NP_771550.1, NP_775405.1, AAO21627.1, AAO21633.1, NP_777469.1, NP_783287.1, Q9ZZ38.1, CAA75485.1, Q37556.1, NP_861489.1, NP_862810.1, NP_879658.1, AAQ23437.1, NP_892281.1, NP_895845.1, CAA73993.1, NP_900618.1, NP_904154.1, NP_904243.1, ZP_00142657.1, AAR24023.1, AAR24025.1, AAR24039.1, AAR24040.1, AAR24041.1, AAR24042.1, AAR24043.1, AAR24044.1, AAR24045.1, AAR24047.1, NP_943693.1, NP_943604.1, NP_943715.1, NP_944685.1, NP_944672.1, CAA52037.1, CAA52038.1, P31839.1, AAC99654.1, NP_957737.1, CAE48116.1, P92558.4, YP_003713.1, AAT08555.1, AAT08568.1, YP_024343.1, AAT45464.1, YP_025886.1, YP_025808.1, NP_848115.2, YP_052884.1, YP_053211.1, CAB44668.1, YP_067721.1, YP_087021.1, YP_097076.1, Q9B6E8.1, BAD66721.1, AAV51342.1, YP_159772.1, YP_169114.1, AAW23973.1, YP_170920.1, YP_173351.1, YP_192901.1, AAW67094.1, AAW67490.1, NP_008406.1, YP_203312.1, YP_203363.1, ZP_00372586.1, YP_209609.1, YP_209562.1, AAX21833.1, AAX54651.1, AAX54664.1, AAX54665.1, AAX54669.1, YP_214967.1, YP_214915.1, AAX94680.1, AAX94697.1, YP_247277.1, YP_247655.1, ZP_00545315.1, YP_266308.1, YP_271951.1, YP_284182.1, YP_292718.1, YP_295191.1, YP_303063.1, AAZ99324.1, YP_313638.1, YP_314907.1, YP_316606.1, NP_050081.1, NP_051114.1, YP_319818.1, YP_323222.1, YP_337877.1, YP_337794.1, YP_360253.1, YP_364577.1, YP_396660.1, AAF72059.1, YP_398383.1, YP_398392.1, YP_398768.1, YP_398928.1, ABB80074.1, YP_422143.1, YP_426649.1, ZP_00943357.1, YP_448681.1, YP_448604.1, YP_448643.1, YP_458215.1, YP_465782.1, YP_469138.1, ABC98217.1, YP_486195.1, YP_492631.1, ABD36070.1, YP_505292.1, YP_505955.1, YP_514908.1, YP_514630.1, NP_059359.1, AAA70292.1, YP_537107.1, YP_537272.1, YP_539026.1, YP_538985.1, YP_538905.1, YP_538813.1, ZP_01263955.1, YP_546162.1, YP_550061.1, YP_567133.1, NP_085565.3, YP_583089.1, YP_588168.1, YP_588310.1, NP_062493.1, NP_062467.1, NP_037618.1, NP_063901.1, NP_064011.1

**NADH dehydrogenase subunit 2:** YP_635684.1, YP_636480.1, YP_636343.1, BAE97249.1, AAG26132.1, ABG76914.1, ABG76921.1, YP_740247.1, YP_740162.1, Q589A5.2, YP_740521.1, YP_740610.1, YP_740696.1, NP_054547.1, NP_039432.1, NP_043076.1, YP_762305.1, NP_054977.1, YP_778535.1, YP_784096.1, YP_784432.1, YP_784519.1, YP_874780.1, YP_874696.1, YP_913231.1, YP_001001579.1, YP_001004229.1, ZP_01731896.1, YP_001109548.1, BAF49814.1, BAF50068.1, BAF50331.1, BAF50419.1, BAF50594.1, YP_001113378.1, YP_001123418.1, YP_001123159.1, ABQ14817.1, ABQ14825.1, ABQ14833.1, ABQ14842.1, ABQ14851.1, ABQ14893.1, ABQ14900.1, ABQ14910.1, ABQ14919.1, ABQ14927.1, ABQ14934.1, Q67IK8.2, Q67IB2.2, Q67IA6.2, P0C159.1, ABR23085.1, ABR23102.1, ABR23105.1, ABR23108.1, ABR23111.1, ABR23117.1, ABR23123.1, ABR23126.1, ABR23129.1, ABR23132.1, YP_001294143.1, YP_001294398.1, YP_001294315.1, YP_001294229.1, ABU85217.1, ABU85370.1, ABU85463.1, ABU85716.1, YP_001531325.1, NP_440041.1, NP_084739.2, YP_001671727.1, YP_001687181.1, YP_001122990.2, YP_001123244.2, YP_001123331.2, YP_001123507.2, YP_001123595.2, YP_001123682.2, YP_001123770.2, YP_001718481.1, YP_001735779.1, YP_001802592.1, P12125.2, Q33532.2, Q9XQ96.1, P46619.2, YP_001837405.1, YP_002000529.1, ZP_03144342.1, ZP_03155721.1, P06256.3, Q9T3G4.3, ACI28289.1, ACI28290.1, ACI28295.1, BAA14330.1, YP_002373662.1, YP_002377323.1, ACP04336.1, NP_683770.1, ZP_04355166.1, YP_002836135.1, AAQ14196.1, NP_897964.1, AAQ64538.1, AAQ64541.1, AAQ64553.1, AAQ64563.1, AAQ64581.1, AAQ64584.1, BAC84683.1, NP_904144.1, AAN31986.1, AAN32017.1, YP_053200.1, YP_054683.1, YP_087011.1, BAA84430.1, YP_209555.1, Q67IA3.2, Q67IB5.2, Q67IC4.2, Q67ID0.2, Q67ID9.2, Q67IE2.2, Q67IG8.2, Q67IL7.2, YP_247644.1, YP_319808.1, YP_398373.1, YP_477700.1, YP_514897.1, YP_538980.1, YP_567123.1, NP_051103.2, YP_588162.1

**NADH dehydrogenase subunit 3:** AAG22493.1, YP_626447.1, ABG49446.1, YP_665673.1, YP_667824.1, YP_673584.1, YP_684389.1, YP_717146.1, YP_717280.1, YP_747147.1, NP_054411.1, ZP_01449209.1, YP_760446.1, YP_762684.1, YP_781437.1, P15956.2, AAG44883.1, NP_074913.1, YP_001020600.1, NP_075446.1, P18630.1, P27062.1, NP_102973.1, CAM76329.1, YP_001206163.1, YP_001234059.1, YP_001249304.1, P92533.2, YP_001315092.1, NP_150127.1, CAC48165.1, CAA89854.1, CAA89888.1, CAA45193.1, YP_001414489.1, YP_001427405.1, YP_001492837.1, YP_001504346.1, YP_001524583.1, YP_001603272.1, NP_064074.2, YP_001621417.1, YP_001630285.1, YP_001648748.1, YP_001661398.1, NP_570150.1, YP_001876498.1, YP_785565.1, YP_002000566.1, YP_002122394.1, ACH47056.1, YP_002213595.1, AAM21470.1, AAM21471.1, YP_002288908.1, YP_002297465.1, CAA73769.1, YP_002363196.1, YP_002587030.1, YP_002608202.1, XP_002338259.1, YP_002608384.1, ZP_03690147.1, AAN01220.1, NP_689390.1, EEH02523.1, ACO50723.1, YP_002860156.1, YP_002860263.1, ZP_00053338.1, EEQ69177.1, YP_002970814.1, YP_002970842.1, YP_002970870.1, YP_002970786.1, YP_002970898.1, NP_705915.1, Q36664.2, AAB88455.1, NP_771559.1, NP_775398.1, CAA32647.1, Q36518.1, NP_075038.2, NP_879651.1, P60159.1, NP_943679.1, NP_943611.1, NP_943732.1, CAA77188.1, YP_025883.1, P16265.3, BAD66784.1, AAW55688.1, AAW55692.1, AAW55704.1, AAW55705.1, AAW55707.1, YP_203331.1, YP_203297.1, YP_203347.1, AAX21825.1, YP_227552.1, Q96007.1, CAA59491.1, YP_313637.1, YP_318500.1, YP_337887.1, YP_398778.1, , ABB83280.1, ABB83284.1, ABB83292.1, ABB83304.1, ABB83306.1, ABB83312.1, ABB83318.1, ABB83320.1, ABB83324.1, ABB83328.1, ABB83332.1, ABB83334.1, YP_422150.1, YP_426642.1, ZP_01047992.1, YP_514649.1, YP_537106.1, YP_537568.1, YP_539030.1, AAF86497.1, AAF86498.1, YP_588354.1, NP_062492.1

**NADH dehydrogenase subunit 5:** BAA07176.1, YP_626451.1, YP_626514.1, YP_626480.1, AAG23669.1, YP_654393.1, YP_654302.1, YP_654283.1, YP_654354.1, YP_654406.1, YP_654341.1, YP_654315.1, YP_654380.1, YP_654289.1, YP_654328.1, YP_654367.1, ABG49157.1, YP_665648.1, YP_665716.1, YP_667834.1, YP_673595.1, CAK02795.1, YP_684395.1, YP_717097.1, YP_717300.1, YP_740441.1, YP_745112.1, NP_049307.1, NP_066415.1, NP_046147.1, NP_038216.1, NP_038172.1, NP_044800.1, NP_066469.1, NP_066346.1, NP_042566.1, NP_054400.1, NP_043727.1, NP_057975.1, YP_760460.1, NP_042248.1, YP_762345.1, YP_762506.1, YP_762702.1, YP_778638.1, YP_783948.1, P11628.2, P05510.2, YP_867515.1, ZP_01547430.1, YP_989081.1, NP_074943.1, YP_001031202.1, CAA47113.1, BAF46415.1, YP_001090211.1, P10330.2, CAA27181.1, CAA38641.1, NP_085478.1, CAM77661.1, YP_001155820.1, YP_001165399.1, YP_001165356.1, ABQ11836.1, ABQ11850.1, YP_001234070.1, YP_001249314.1, ZP_01903125.1, YP_001315130.1, NP_150132.1, NP_150324.1, NP_150413.1, ABS50632.1, YP_001419499.1, ABU50153.1, YP_001427401.1, YP_001492710.1, YP_001492836.1, YP_001504356.1, YP_001023655.2, ABX45181.1, NP_064039.2, ZP_02187174.1, YP_001633646.1, YP_001630296.1, YP_001609571.1, YP_001648476.1, YP_001648453.1, YP_001648522.1, YP_001648592.1, YP_001648606.1, YP_001648494.1, YP_001648544.1, YP_001648490.1, YP_001648678.1, YP_001648629.1, YP_001648422.1, YP_001648587.1, YP_001648550.1, YP_001648573.1, YP_001648669.1, YP_001648743.1, YP_001661395.1, ABZ79356.1, ABZ79358.1, YP_001684446.1, YP_001687114.1, YP_001742104.1, P50367.1, P50368.1, YP_539016.2, YP_001797650.1, NP_570156.1, YP_001874762.1, YP_001876486.1, YP_001876502.1, ACD45548.1, ACD45549.1, ACD45550.1, ACD45551.1, ACD45552.1, ACD45553.1, YP_001936267.1, YP_001936204.1, YP_001938279.1, ACD85989.1, YP_001994425.1, YP_002000552.1, YP_002149701.1, YP_002149675.1, YP_002213599.1, ACH78247.1, YP_002220656.1, YP_002221371.1, YP_002221398.1, YP_002221531.1, NP_612816.1, CAA73263.1, YP_002274317.1, ZP_03287564.1, NP_659279.1, AAL65275.1, YP_002363207.1, YP_002426997.1, YP_002549243.1, YP_002587020.1, YP_002608190.1, XP_002337651.1, YP_002608337.1, YP_002613449.1, ZP_03690136.1, , YP_002664092.1, YP_002684054.1, NP_689376.1, EEH02520.1, ACO50732.1, AAN04074.1, YP_002860129.1, YP_002860275.1, ZP_00053039.1, NP_696972.1, EEQ69170.1, EEQ83192.1, YP_002970811.1, YP_002970839.1, YP_002970867.1, YP_002970783.1, YP_002970895.1, EER36340.1, NP_705908.1, YP_002971994.1, ZP_04774974.1, ACS71774.1, Q37680.1, NP_775402.1, Q01561.2, AAP33164.1, CAA73986.1, NP_943684.1, NP_943710.1, AAC99648.1, P29388.3, NP_957735.1, YP_025852.1, YP_033688.1, YP_052886.1, CAF28992.1, CAF28993.1, AAT74903.1, AAT74908.1, CAF22013.1, CAF22014.1, CAF22015.1, CAF22016.1, CAF22017.1, CAF22018.1, CAF22019.1, CAF22020.1, CAF22021.1, BAD66755.1, YP_173349.1, AAW47305.1, AAW67487.1, YP_203299.1, YP_203365.1, YP_209597.1, Q8HHD2.1, NP_009258.1, YP_214874.1, YP_214966.1, YP_214914.1, YP_232805.1, YP_232819.1, CAA87754.1, YP_266312.1, YP_271950.1, YP_303111.1, YP_313630.1, , YP_316588.1, NP_050105.1, YP_337890.1, YP_344537.1, AAF72053.1, YP_398408.1, YP_398781.1, YP_422139.1, YP_426653.1, YP_448707.1, YP_448628.1, YP_448667.1, ABC98213.1, YP_492646.1, YP_507366.1, ZP_01128208.1, YP_537102.1, YP_537257.1, ZP_01263951.1, YP_588334.1, NP_062497.1, NP_037615.1

**Chloroplast psbA:** AAD09838.1, YP_635976.1, YP_635726.1, YP_635913.1, YP_636207.1, YP_636279.1, ABH09268.1, ABH88068.1, YP_717233.1, YP_717885.1, YP_720146.1, YP_724190.1, YP_729600.1, YP_731062.1, YP_731060.1, ABI54129.1, NP_054477.1, NP_039291.1, NP_043238.1, ZP_01468085.1, ZP_01471014.1, ZP_01472718.1, CAA30818.1, ZP_01618758.1, ZP_01629901.1, ZP_01630934.1, ZP_01631344.1, ABL86488.1, ABL86514.1, ABL86556.1, ABL86562.1, Q2QDA8.1, YP_001004166.1, YP_001008639.1, YP_001016426.1, YP_001019070.1, YP_001023677.1, ZP_01728312.1, P02956.1, P18290.1, P09752.1, P07063.1, P04999.3, P15191.1, P11848.3, P14660.1, CAL36200.1, CAL36201.1, CAL36216.1, CAL36294.1, NP_084778.1, YP_001123266.1, YP_001123617.1, YP_001123011.1, YP_001123706.1, YP_001122927.1, YP_001224507.1, YP_001227696.1, YP_001227697.1, YP_001293511.1, YP_001294164.1, ABU85627.1, YP_001430089.1, YP_001468288.1, CAO89050.1, YP_001531202.1, YP_001542512.1, YP_001550131.1, AAL14703.1, NP_439906.1, YP_001656036.1, AAL30839.1, AAL30840.1, ACA98171.1, YP_001734665.1, YP_001718638.1, P51765.1, P51764.1, YP_001122788.2, NP_487612.1, NP_488906.1, YP_001865804.1, YP_001868705.1, NP_569608.1, YP_001936497.1, YP_002049188.1, ZP_03144454.1, ZP_03153624.1, ZP_03157178.1, ACH47559.1, ACH47561.1, ACH47562.1, ACH47564.1, ACH47565.1, ACH47566.1, BAG70962.1, ZP_03275474.1, ZP_03276271.1, YP_002375507.1, YP_002376371.1, YP_002481806.1, YP_002482229.1, NP_682267.1, NP_682633.1, NP_682634.1, YP_002598066.1, YP_002597358.1, YP_002597138.1, YP_002586944.1, YP_002600883.1, YP_002600858.1, YP_002601066.1, YP_002619915.1, YP_002710518.1, YP_002715112.1, ZP_03765763.1, YP_002808517.1, YP_002808606.1, NP_683826.1, YP_001733427.2, YP_002836070.1, ACQ91338.1, ACQ91348.1, ACQ91392.1, ACQ91410.1, ACQ91420.1, YP_002970689.1, AAN85782.1, AAN85784.1, AAN85785.1, AAN85786.1, AAN85790.1, AAN85801.1, AAN85803.1, AAN85817.1, NP_777405.1, NP_848104.1, NP_874646.1, NP_892343.1, NP_894252.1, NP_897076.1, NP_897563.1, NP_898010.1, AAQ67339.1, NP_904209.1, NP_925602.1, AAP70595.1, AAR08455.1, AAR08459.1, AAR08460.1, AAR08465.1, AAR08469.1, AAR19417.1, BAA76900.1, AAT02715.1, AAT02717.1, AAT02718.1, AAT02721.1, AAT02739.1, AAT02740.1, CAA78895.1, CAF33061.1, AAT57704.1, AAT57706.1, AAT57707.1, AAT57722.1, AAT57725.1, AAT57730.1, AAT57743.1, AAT57748.1, AAT57751.1, AAT57755.1, AAT57777.1, CAF32255.1, CAF32321.1, AAD40182.1, AAU84560.1, P35860.1, AAV97758.1, YP_170876.1, YP_171357.1, YP_171803.1, BAA82767.1, YP_195211.1, YP_209536.1, AAX46196.1, YP_214516.1, YP_214746.1, YP_214204.1, AAY23671.1, AAY23672.1, AAY23674.1, ZP_00518078.1, YP_247579.1, YP_291752.1, NP_045767.1, NP_051039.1, YP_322115.1, YP_322653.1, ABA02341.2, YP_376953.1, YP_377815.1, YP_380638.1, YP_381355.1, YP_396723.1, ABB82248.1, ZP_01079909.1, ZP_01080376.1, ZP_01080882.1, ZP_01084150.1, ZP_01086100.1, ZP_01122807.1, ZP_01125105.1, ABD97856.1, YP_538745.1

**Chlorophyll alpha apoprotein A2:** YP_635999.1, YP_635638.1, YP_635878.1, YP_636558.1, YP_636557.1, YP_636274.1, YP_717253.1, YP_724109.1, YP_729625.1, CAA29287.1, NP_038393.1, NP_039298.1, NP_043151.1, NP_050884.1, YP_764416.1, ZP_01469429.1, ZP_01472984.1, YP_817481.1, ZP_01620268.1, YP_001019112.1, YP_001023700.1, ABN59036.1, ABN59037.1, ABN59038.1, ABN59040.1, ABN59043.1, ABN59044.1, ABN59045.1, ABN59047.1, ABN59048.1, ABN59050.1, ABN59052.1, ABN59053.1, ABN59054.1, ABN59059.1, ABN59065.1, ABN59070.1, ABN59072.1, ABN59076.1, ABN59077.1, ABN59079.1, ABN59081.1, ABN59087.1, ABN59089.1, ABN59090.1, ABN59091.1, ABN59095.1, ABN59098.1, ABN59100.1, ABN59101.1, ABN59106.1, ABN59107.1, ABN59113.1, ABN59116.1, ABN59118.1, ABN59119.1, ABN59120.1, ABN59122.1, ABN59128.1, ABN59129.1, ABN59130.1, ABN59133.1, ZP_01732147.1, YP_001224115.1, YP_001228413.1, YP_001382198.1, AAA88634.1, ABU88236.1, ABU88302.1, YP_001468307.1, 1JB0, CAO88673.1, NP_440758.1, YP_001659771.1, YP_001735205.1, YP_001802407.1, YP_002000444.1, YP_002049203.1, ZP_03141713.1, ZP_03156342.1, ACI31251.1, ZP_03275302.1, YP_002485337.1, YP_002519584.1, ACM51423.1, NP_681521.1, YP_002598028.1, YP_002586937.1, YP_002600933.1, YP_002600994.1, YP_002616070.1, YP_002714875.1, YP_002808519.1, YP_002808607.1, ACP50937.1, ACP51427.1, ACP51926.1, ACP52236.1, ACP52360.1, ACP52420.1, ACP52549.1, YP_002905117.1, NP_777412.1, NP_817254.1, NP_898214.1, NP_904203.1, NP_958404.1, CAA41630.1, YP_063613.1, YP_086965.1, P36492.1, YP_172755.1, YP_209530.1, ZP_00518087.1, AAZ04112.1, NP_045852.1, YP_377995.1, YP_380663.1, YP_398328.1, ZP_01080852.1, ZP_01083716.1, ZP_01123437.1, YP_514851.1, YP_588116.1

**Chlorophyll a binding protein:** YP_635942.1, YP_635899.1, YP_636224.1, BAB18400.1, BAB18401.1, BAB18402.1, BAB18403.1, BAB18407.1, BAB18411.1, BAB18412.1, BAB18420.1, BAB18421.1, BAB18422.1, BAB18423.1, BAB18424.1, BAB18425.1, BAB18426.1, BAB18427.1, BAB18430.1, BAB18431.1, BAB18433.1, BAB18435.1, BAB18438.1, BAB18446.1, BAB18447.1, BAB18451.1, BAB18453.1, BAB18454.1, BAB18455.1, BAB18456.1, YP_720441.1, YP_721411.1, YP_722165.1, YP_731503.1, YP_740198.1, YP_740561.1, YP_764370.1, YP_784382.1, ZP_01468016.1, AAA85378.1, BAF35220.1, YP_913183.1, ZP_01628976.1, ZP_01628977.1, ZP_01628980.1, ZP_01628981.1, ZP_01631891.1, Q06J66.1, Q0P3Q1.1, ZP_01728530.1, ZP_01728798.1, Q9MUW1.1, CAC42494.1, CAC42495.1, CAC42496.1, CAC42499.1, CAC42500.1, CAC42501.1, CAC42524.1, CAC42518.1, CAC42514.1, YP_001294348.1, CAC42519.2, CAC42497.2, YP_001382154.1, AAA27359.1, ABU85345.1, ABU88309.1, ABW22786.1, YP_001520254.1, YP_001521552.1, CAO86311.1, CAO88879.1, YP_001542527.1, NP_441119.1, NP_441268.1, BAC09183.2, YP_001659129.1, YP_001734545.1, YP_001734806.1, YP_001718433.2, YP_778561.2, YP_001671679.2, YP_001802076.1, YP_001803201.1, Q9BBT1.2, P56778.2, NP_488041.1, NP_488042.1, NP_488043.1, NP_488331.1, YP_001866969.1, P09193.3, NP_043248.3, NP_045800.2, YP_001123369.2, NP_050846.2, NP_038349.2, YP_717204.2, NP_683824.3, YP_636447.2, YP_002000374.1, ZP_03143708.1, ZP_03153756.1, ACI31242.1, ZP_03274431.1, ZP_03275214.1, YP_002375907.1, YP_002377095.1, BAC06404.1, BAC06406.1, BAC06414.1, BAC06440.1, BAC06441.1, YP_002480974.1, YP_002483153.1, Q32RS9.2, Q8M9W5.2, NP_681841.1, NP_682421.1, AAM96540.1, YP_002600929.1, YP_002601065.1, YP_002710337.1, YP_002713447.1, YP_002712977.1, YP_002720108.1, YP_002808605.1, , ACP52363.1, YP_002905184.1, 3A0B, CAA96551.1, P56308.1, BAC77265.1, NP_862750.1, AAQ05885.1, NP_904095.1, NP_925270.1, CAE47090.1, P31157.1, NP_958422.1, AAA27291.1, YP_170712.1, YP_171582.1, Q08684.1, AAX45723.1, ZP_00516495.1, ZP_00519242.1, AAZ04079.1, Q6Q972.1, Q9F487.1, Q9F488.1, P95503.3, AAC64212.1, YP_321762.1, ABA42219.1, YP_358574.1, YP_377013.1, YP_381894.1, YP_399675.1, ABB92216.1, YP_475982.1, YP_478470.1, YP_538759.1
